# Discovery and optimisation of a covalent ligand for TRIM25 and its application to targeted protein ubiquitination†

**DOI:** 10.1039/d5sc01540e

**Published:** 2025-05-12

**Authors:** Katherine A. McPhie, Diego Esposito, Jonathan Pettinger, Daniel Norman, Thilo Werner, Toby Mathieson, Jacob T. Bush, Katrin Rittinger

**Affiliations:** a Molecular Structure of Cell Signalling Laboratory, The Francis Crick Institute 1 Midland Road London NW1 1AT UK katrin.rittinger@crick.ac.uk; b Crick-GSK Biomedical LinkLabs, GSK Gunnels Wood Road, Stevenage Hertfordshire SG1 2NY UK; c Chemical Biology, GSK Gunnels Wood Road Stevenage Hertfordshire SG1 2NY UK; d Cellzome GmbH, a GSK Company Meyerhofstrasse 1 Heidelberg 69117 Germany

## Abstract

The tripartite motif (TRIM) family of RING-type E3 ligases catalyses the formation of many different types of ubiquitin chains, and as such, plays important roles in diverse cellular functions, ranging from immune regulation to cancer signalling pathways. Few ligands have been discovered for TRIM E3 ligases, and these E3s are under-represented in the rapidly expanding field of induced proximity. Here we present the identification of a novel covalent ligand for the PRYSPRY substrate binding domain of TRIM25. We employ covalent fragment screening coupled with high-throughput chemistry direct-to-biology optimisation to efficiently elaborate covalent fragment hits. We demonstrate that our optimised ligand enhances the *in vitro* auto-ubiquitination activity of TRIM25 and engages TRIM25 in live cells. We also present the X-ray crystal structure of TRIM25 PRYSPRY in complex with this covalent ligand. Finally, we incorporate our optimised ligand into heterobifunctional proximity-inducing compounds and demonstrate the *in vitro* targeted ubiquitination of a neosubstrate by TRIM25.

## Abstract 2

Covalent fragment screening coupled with ‘direct-to-biology’ optimisation has identified covalent binders of TRIM25 PRYSPRY, expanding the repertoire of liganded E3 ligases and providing new avenues for targeted protein ubiquitination.

## Graphical abstract

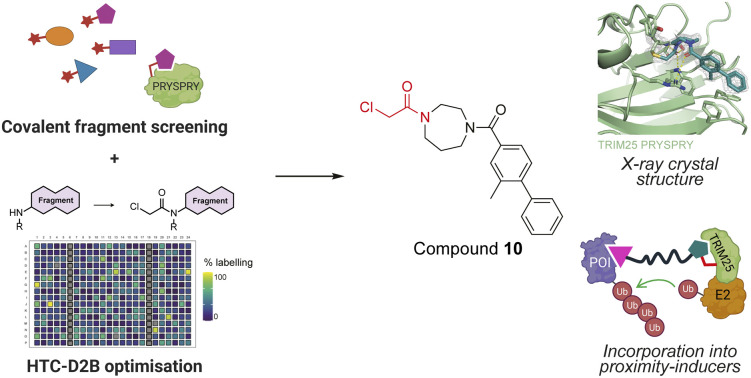

## Introduction

E3 ubiquitin ligases form a diverse family of proteins that mediate ubiquitination, a critical post-translational modification involved in regulating the majority of cellular processes, including protein degradation, cell signalling and DNA damage response.^1,2^ Ubiquitination occurs *via* an ATP-dependent cascade involving three enzymes: an E1-activating enzyme, an E2-conjugating enzyme, and an E3 ligase enzyme. The initial ubiquitin modification can be further extended into structurally diverse polyubiquitin chains, linked *via* one of ubiquitin's seven lysine (Lys) residues, or N-terminal methionine (Met).^3^ The >600 human E3 ligases provide substrate specificity, and often work in synergy with specific E2-conjugating enzymes (of which there are ∼40) to regulate ubiquitin chain architecture and topology.^4^

Induced proximity modalities that exploit the role of ubiquitination in proteasomal degradation have emerged as powerful tools and therapeutic strategies. Heterobifunctional molecules, called proteolysis targeting chimeras (PROTACs), are used to redirect E3 ligases to modify disease-causing proteins with Lys48-linked ubiquitin chains, thus inducing their proteasomal degradation.^5^ Targeted protein degradation (TPD) *via* non-proteasomal pathways, such as lysosomal degradation and autophagy, has also been demonstrated,^6–8^ but never through direct engagement of an E3 ligase that activates these pathways (*via* Lys11- or Lys63-linked ubiquitin chains). Identifying ligands for these Lys11- or Lys63-specific E3 ligases could enable alternative non-proteasomal degradation strategies. In recent years, the field of induced proximity has expanded beyond TPD to hijack alternative enzymes such as deubiquitinating enzymes and phosphatases.^9,10^ Despite the diverse roles of E3 ligases in regulating the majority of cellular processes, only a small proportion of the >600 E3 ligases have been liganded and repurposed for induced proximity modalities.^11,12^ The discovery of new ligands for E3 ligases, particularly those with non-degradative ubiquitination activity, presents a promising strategy to activate alternative cellular outcomes for disease-related proteins.^13^

**Fig. 1:**
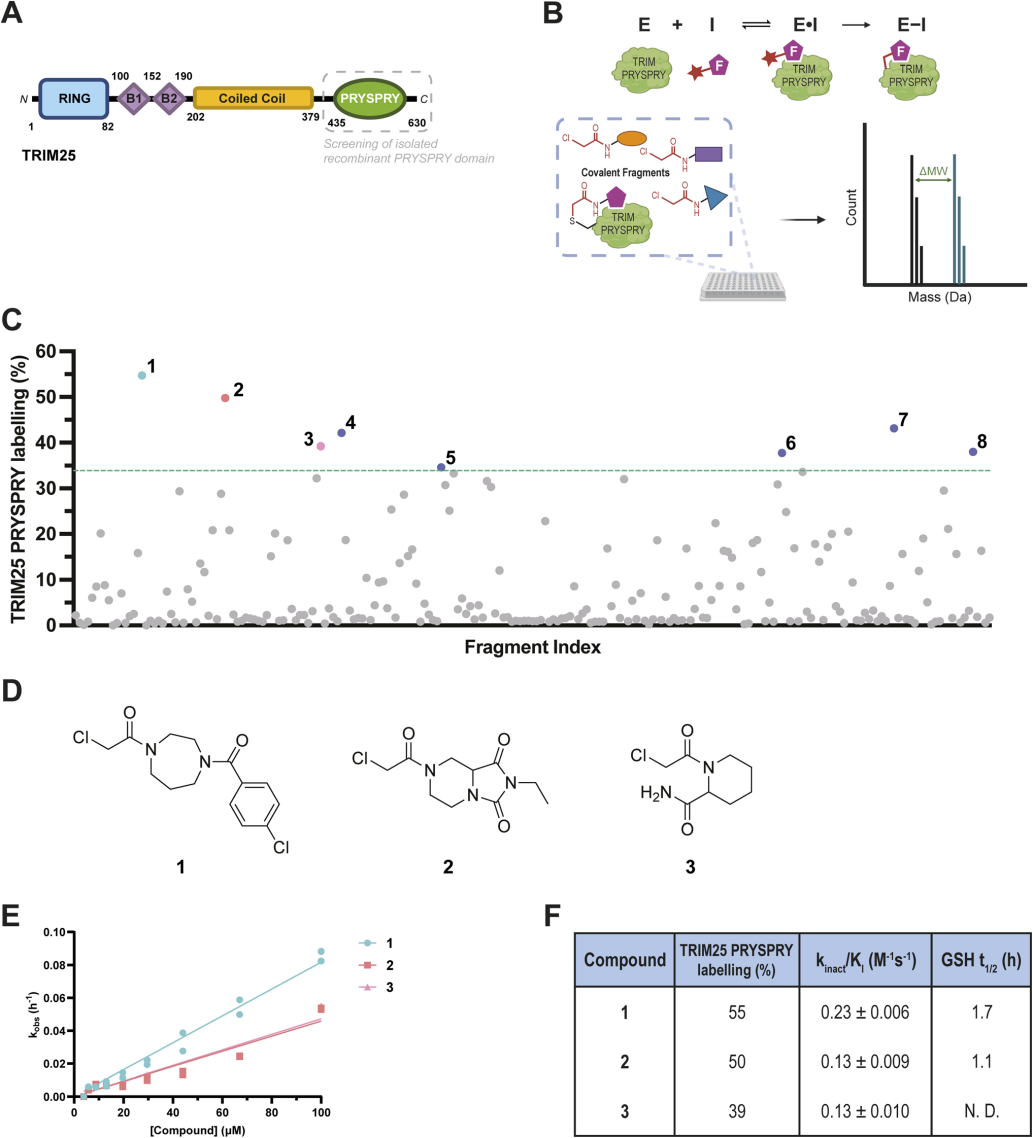
Covalent fragment screening against recombinant TRIM25 PRYSPRY. (A) Cartoon representation of the TRIM25 protein with domain boundaries: RING domain (blue), B-box domain (purple), coiled coil domain (yellow), and PRYSPRY domain (green); (B) schematic of irreversible covalent binding equilibrium kinetics and intact protein LCMS assay. Created in BioRender. McPhie, K. (2025) https://BioRender.com/e02g172; (C) summary of covalent fragment screening by intact protein LCMS. % labelling of 221 chloroacetamides (50 μM) against TRIM25 PRYSPRY (0.25 μM) at 4 °C for 24 h. The green line represents the mean + 2SD; (D) chemical structures of hit fragments 1–3 for TRIM25 PRYSPRY selected for HTC-D2B progression. Chemical structures of hit fragments 4–8 are shown in Fig. S1A, ESI;† (E) *k*_obs_ (h^−1^) plotted against concentration (μM), fitted using a straight line fit. Data are presented as *n* = 2, mean ± SE of fit; (F) table of % labelling, *k*_inact_/*K*_I_ (M^−1^ s^−1^) values, and *t*_1/2_ (h) (in the presence of 4 mM GSH) for fragments 1–3. Kinetic characterisation for hit fragments 4–8 is shown in Fig. S3, ESI.†

We sought to assess whether the E3 ligase TRIM25, reported to catalyse the formation of both Lys48- and Lys63-linked ubiquitin chains,^14^ could be liganded and repurposed for targeted protein ubiquitination. TRIM25 is a member of the TRIM family of RING-type E3 ligases and comprises a canonical N-terminal tripartite motif (TRIM) and a variable C-terminal PRYSPRY substrate binding domain (Fig. 1A).^15^ As the majority of current proximity-inducing small molecules for E3s recruit substrates to the physiological substrate binding component (*e.g.* the substrate adaptor of Cullin RING E3s),^11^ we focused our efforts towards liganding the PRYSPRY substrate binding domain of TRIM25. TRIM25 has been reported to ubiquitinate a number of different substrates, possibly in some cases mediated through RNA binding,^16^ including RIG-I,^15,17,18^ DDX3X^19^ and ZAP,^20–22^ with diverse roles in immune regulation, cancer signalling pathways and antiviral activity.^23–26^ As such, TRIM25 is a promising candidate for redirection to a variety of neosubstrates, and the development of novel chemical tools that target TRIM25 could help to deconvolute its many proposed functions.

Within the TRIM family, only one other protein, TRIM21, has been repurposed for targeted protein ubiquitination and degradation. The TPD strategy called ‘Trim-Away’ harnesses TRIM21's innate ability to cluster around antigens through binding to the Fc receptor of antibodies.^27,28^ More recently, small molecule binders of TRIM21, functioning as both molecular glues and components of heterobifunctional PROTACs, have been demonstrated to selectively degrade multimeric protein complexes.^29^ Additionally, small molecule ligands for the PRYSPRY domains of two other PRYSPRY-containing TRIM E3 ligases, TRIM7 and TRIM58, have been described,^30,31^ providing first evidence for the ligandability of the PRYSPRY substrate binding domain.

To identify ligands for the TRIM25 PRYSPRY domain, we employed a covalent fragment-based discovery approach, using intact protein liquid chromatography mass spectrometry (LCMS) to screen for covalent binders (Fig. 1B). Covalent fragments comprise a reversible molecular recognition motif (typically <300 Da), and an electrophilic ‘warhead’ for covalent modification to proximal nucleophilic residues, such as cysteine (Cys). This covalent functionality overcomes limitations associated with the modest reversible affinity of fragments and improves the ease and sensitivity of hit detection.^32^ Moreover, covalent ligands enable the targeting of traditionally ‘undruggable’ shallow protein surfaces, often found in scaffold proteins or mediators of protein–protein interactions (PPIs), such as the PRYSPRY domain.^33^ With a well-tuned electrophile, covalent ligands offer high potency and selectivity driven by increased occupancy of the irreversible modification. Furthermore, covalent ligands have successfully been applied to induced proximity modalities, as demonstrated for several members of the CRL (Cullin-RING ligase) and RING-type E3 families, including DCAF1, DCAF16, RNF114, and FEM1B.^34–37^ While most heterobifunctional molecules employ reversible ligands to facilitate a catalytic mechanism of action, there is an increasing use of covalent ligands targeting the E3 ligase component.^38–41^ This can be advantageous, enabling potent degradation even at low ligand occupancy, as well as improved physicochemical properties of traditionally large (>500 Da) heterobifunctional molecules, often due to the typically smaller size of covalent ligands.^42,43^

In this study, we coupled covalent fragment screening with our recently reported high-throughput chemistry direct-to-biology (HTC-D2B) chloroacetamide fragment elaboration platform.^44^ HTC-D2B enabled efficient fragment optimisation and facilitated the rapid selection of optimised covalent binders of TRIM25 PRYSPRY for resynthesis and downstream validation. Optimised binders were characterised and incorporated into heterobifunctional molecules capable of repurposing TRIM25 to ubiquitinate a neosubstrate *in vitro*.

## Results and discussion

### Covalent fragment screening identifies binders of the TRIM25 PRYSPRY domain

To explore the ligandability of TRIM25 PRYSPRY, we employed a binding site-agnostic covalent fragment screening approach. Intact protein liquid chromatography mass spectrometry (LCMS) was used to screen a library of 221 cysteine-reactive chloroacetamide fragments^44,45^ (ESI Data S1†) at 50 μM against the recombinantly expressed TRIM25 PRYSPRY domain (0.25 μM), with incubation at 4 °C for 24 hours (Fig. 1C). Percentage labelling by each covalent fragment was assessed, by comparing the relative intensities of apo protein and protein–fragment complexes. Fragments were selected as hits if the percentage labelling was greater than 33.9% (mean of labelling across the whole library + 2SD). We identified eight fragment hits (1–8, Fig. 1D and S1A, ESI†) that surpassed this threshold, representing a 3.6% hit rate. All hits were observed to modify the TRIM25 PRYSPRY domain with a majority single labelling event (Fig. S1B, ESI†). We counter-screened against the PRYSPRY domain of a related TRIM protein, TRIM21, which has received significant attention within the induced proximity field (Fig. S2, ESI†).^27,29^ No significant labelling of TRIM21 PRYSPRY for any fragment within the chloroacetamide fragment library was observed, despite the presence of solvent-accessible Cys residues in both TRIM21 and TRIM25 PRYSPRY domains.

To validate covalent binding of fragment hits 1–8 and to elucidate the rate of covalent labelling, kinetic characterisation was performed. A 10-point dilution series of each fragment hit was incubated with TRIM25 PRYSPRY (0.5 μM) at 4 °C and sampled at eight different timepoints across 24 hours (Fig. S3A, ESI†). The pseudo-first order rate constant (*k*_obs_) was plotted for each concentration (Fig. 1E and S3B, ESI†), and from the gradient of the fitted straight line, the second-order rate constant, *k*_inact_/*K*_I_, for each fragment was determined (Fig. S3C, ESI†). The three fragments with the highest *k*_inact_/*K*_I_ values, fragments 1, 2 and 3 (Fig. 1F), were selected for elaboration using high-throughput chemistry direct-to-biology (HTC-D2B) optimisation approaches.

The intrinsic reactivity of chloroacetamide fragment hits 1 and 2 was also assessed using an LCMS-based glutathione (GSH) assay to measure compound half-life (*t*_1/2_). Fragment 3 does not contain a UV-active chromophore, so it was incompatible with the LCMS-based reactivity assay. In the presence of 4 mM GSH, *t*_1/2_ values of 1.7 h and 1.1 h for 1 and 2, respectively, were calculated and used as a benchmark for further covalent optimisation (Fig. 1F and S3D, ESI†). These half-life values fall within the expected range for covalent inhibitors,^44^ suggesting 1 and 2 are good starting points for further optimisation.

### HTC-D2B optimisation enables efficient discovery of potent covalent ligands that enhance TRIM25 auto-ubiquitination activity

**Fig. 2:**
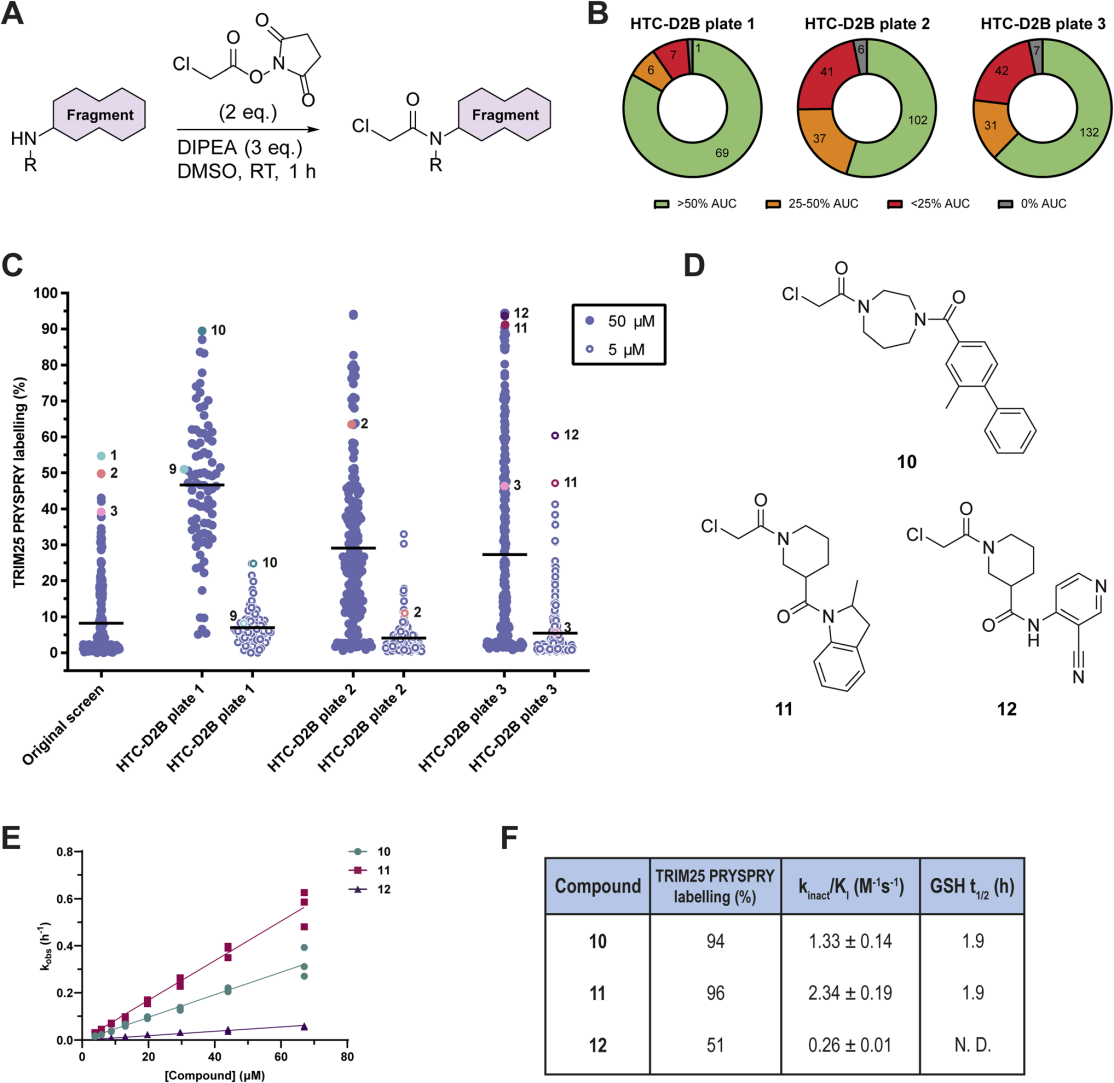
High-throughput chemistry direct-to-biology (HTC-D2B) optimisation of fragments 1–3. (A) HTC reaction scheme;^44^ (B) pie charts of HTC conversion analysed by LCMS across HTC-D2B plate 1 (83 compounds, based on 1), HTC-D2B plate 2 (186 compounds, based on 2), and HTC-D2B plate 3 (212 compounds, based on 3). % AUC (area under the curve) represents AUC for the product-containing peak relative to the starting material-containing peak, selected on identification of the expected *m*/*z*;^47^ (C) comparison of % labelling of TRIM25 PRYSPRY (0.5 μM) by the original fragment library (50 μM) and each HTC-D2B library (50 μM and 5 μM) at 4 °C for 24 h. Black lines represent mean % labelling. Fragment 1 was not included in HTC-D2B plate 1, so a close analogue is highlighted instead (9, structure shown in Fig. S4B, ESI†); (D) chemical structures of optimised chloroacetamides selected for progression; (E) *k*_obs_ (h^−1^) plotted against concentration (μM), fitted using a straight line fit. Data are presented as *n* = 3; (F) table of % labelling, *k*_inact_/*K*_I_ (M^−1^ s^−1^) values (mean ± SD, *n* = 3), and *t*_1/2_ (h) (in the presence of 4 mM GSH) for compounds 10, 11 and 12.

To optimise fragments 1–3, we employed a high-throughput chemistry direct-to-biology (HTC-D2B) strategy (Fig. S4A, ESI†). We have previously described the 384-well plate-based HTC-D2B platform,^44,45^ where a library of elaborated parent amines is designed based on structural similarity to the hit fragment. A single-step amide coupling reaction is performed to install the reactive chloroacetamide electrophile (Fig. 2A), and the resulting elaborated chloroacetamides are screened without purification, by intact protein LCMS. The speed and minimal handling between synthesis and biological screening enable rapid exploration of chemical space to identify improved binders, which can then be purified for confirmation and accurate potency determination.

Three separate HTC-D2B hit expansion libraries were designed based on fragments 1–3. For each fragment, a Tanimoto-based similarity search^46^ around the parent amine (1a–3a, Fig. S4A, ESI†) was performed, filtering for readily available amines with a molecular weight between 110 and 350 Da. This resulted in a curated library of 83 parent amines based on fragment 1 (HTC-D2B plate 1), 212 parent amines based on fragment 2 (HTC-D2B plate 2), and 186 parent amines based on fragment 3 (HTC-D2B plate 3). Parent amine 1a was not available for inclusion in HTC-D2B plate 1, so a matched molecular pair analogue was included instead (9, structure shown in Fig. S4B, ESI†). For all three libraries, installation of the chloroacetamide electrophile by HTC proceeded with satisfactory chemical conversion (Fig. 2B). Following a hydroxylamine quench, the three libraries (50 μM, 481 chloroacetamides total) were incubated with TRIM25 PRYSPRY (0.5 μM) at 4 °C for 24 hours and screened by intact protein LCMS. A significant improvement in labelling was observed across the libraries (Fig. 2C). To triage the best hits, the libraries were also screened at 5 μM (Fig. 2C). Synthetic tractability and percentage labelling were evaluated for selecting optimised HTC-D2B compounds for progression. Additionally, compounds with low chemical conversion but high protein labelling at 5 μM were considered particularly efficient labellers and were prioritised over compounds with high chemical conversion and comparable protein labelling. Consequently, three optimised HTC-D2B compounds 10–12 were selected for resynthesis and purification (Fig. 2D and S4C–E, ESI†).

Optimised hits 10–12 were resynthesised and purified for further characterisation (Scheme S1–S3, ESI†). As before, the rate of labelling (*k*_inact_/*K*_I_) was measured (Fig. S5A, ESI†). The *k*_inact_/*K*_I_ values of compounds 10 and 11 were calculated as 1.33 ± 0.14 M^−1^ s^−1^ and 2.34 ± 0.19 M^−1^ s^−1^, a 6-fold improvement over hit fragment 1 and an 18-fold improvement over hit fragment 3, respectively (Fig. 2E and F). Unfortunately, purified compound 12 did not reproduce the labelling observed in HTC-D2B screening (Fig. S4E, ESI†), and did not show a notable improvement in kinetics. Therefore, 12 was not progressed any further.

Additionally, compound 11, a mixture of diastereomers, was further purified into its component diastereomers (11-1, 11-2, 11-3 and 11-4, Fig. S5B, ESI†). Although we were unable to assign absolute stereochemistry, we observed a clear difference in *k*_inact_/*K*_I_ values between enantiomers 11-1 and 11-2, and enantiomers 11-3 and 11-4, suggesting a stereoselective preference for TRIM25 PRYSPRY (Fig. S5C–E, ESI†).

To ensure that the observed increase in *k*_inact_/*K*_I_ for compounds 10 and 11 was driven by an increase in the potency of reversible molecular recognition, rather than an increase in the intrinsic reactivity of the chloroacetamide electrophile, *t*_1/2_ values for 10 and 11 were also obtained. As before, the rate of covalent modification with GSH was measured using an LCMS-based assay. Compounds 10 and 11 had a calculated half-life of 1.9 h in the presence of 4 mM GSH, confirming that the HTC-D2B campaign had not increased the reactivity of the chloroacetamide electrophile (Fig. 2F and S3D, ESI†). Moreover, the measured *t*_1/2_ values of 10 and 11 were comparable to that of the approved cysteine-reactive covalent cancer therapeutic, osimertinib (*t*_1/2_ = 1.3 h),^44,48^ demonstrating the value of chloroacetamide-based tool molecules.

**Fig. 3:**
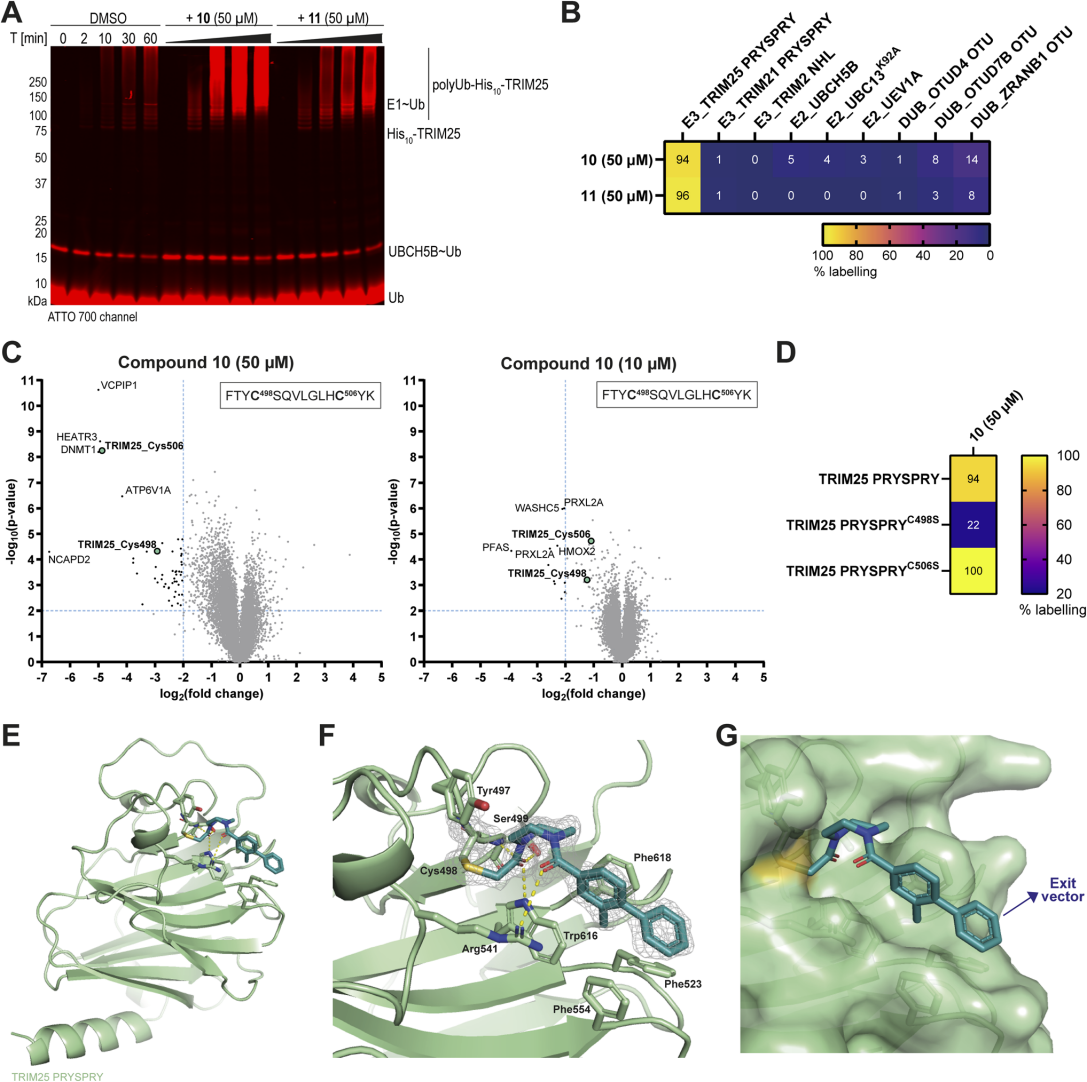
Biochemical characterisation, target engagement and crystal structure of compound 10. (A) Auto-ubiquitination time course assays with TRIM25 pre-treated with either DMSO or compound 10 or 11. TRIM25 (4 μM) was incubated with compounds (50 μM) or DMSO (1%) for 16 h at 4 °C, before addition of E1 (0.2 μM), UBCH5B (2 μM), Ub (50 μM), and Ub^ATTO^ (1 μM). The assay was initiated by the addition of ATP and performed for 60 min at 30 °C. Time point 0 was taken before the addition of ATP. The samples were analysed by SDS-PAGE, with Coomassie staining and scanning at 700 nm wavelength (for ATTO emission). Coomassie gel is shown in Fig. S6B, ESI;† (B) Heatmap of % labelling of purified chloroacetamides 10 and 11 (50 μM) against a panel of recombinant Ub system proteins (10 μM) with incubation at 4 °C for 24 h, and analysis by intact protein LCMS; (C) cellular target identification for 10 (50 μM, left, and 10 μM, right, 4 h incubation at 37 °C in live THP-1 cells) using an iodoacetamide desthiobiotin (IA-DTB) probe-based competitive profiling approach. Target engagement is considered significant if FC ≤ 0.25; *p*-value ≤ 0.01 (top left corner, outlined by dotted lines). Significantly competed sites Cys498 and Cys506 on the TRIM25 peptide, FTYC^498^SQVLGLHC^506^YK, are highlighted in teal. The top five significantly competed off-target Cys sites are highlighted in grey. Volcano plots for compound 11 are shown in Fig. S6E, ESI;† (D) labelling of 10 (50 μM) using recombinant TRIM25 PRYSPRY cysteine mutants (10 μM) by intact protein LCMS, with incubation at 4 °C for 24 h; (E) X-ray crystal structure of 10 (teal) bound to TRIM25 PRYSPRY (pale green) with protein–fragment contacts displayed (dashed, yellow), PDB 9I0T; (F) X-ray crystal structure of 10 (teal) bound to TRIM25 PRYSPRY (pale green) with protein–fragment contacts displayed (dashed, yellow) and 2Fo-Fc density maps displayed at *σ*-level 1.0 (grey mesh); (G) X-ray crystal structure of 10 (teal) bound to TRIM25 PRYSPRY (pale green) with the protein surface displayed and an exit vector for heterobifunctional compound design indicated by a blue arrow.

To test if covalent binding of compounds 10 and 11 affected TRIM25 catalytic activity, *in vitro* auto-ubiquitination assays were performed using recombinant proteins to reconstitute the ubiquitin system (Fig. S6A, ESI†). Full-length recombinant TRIM25 was pre-treated with either DMSO, 10 or 11 for 16 hours at 4 °C, prior to performing the auto-ubiquitination assay. Interestingly, the rate of TRIM25 auto-ubiquitination was significantly increased by compounds 10 and 11 (Fig. 3A, S6B and C, ESI†). We speculate that the compounds may be acting as molecular glues to cluster TRIM25 molecules and stabilise a higher-order oligomeric state of TRIM25, which is known to be required for TRIM E3 activity.^49,50^ Alternatively, the compounds may be inducing the stabilisation of a particular conformation where a greater number of Lys residues are accessible for ubiquitination.

### TRIM25 is engaged by compound 10 in live cells and the protein–ligand complex crystal structure gives insight into binding mode

We next explored the selectivity of 10 and 11, as well as their target engagement in a cellular context. First, compounds 10 and 11 were screened against a panel of recombinant ubiquitin system proteins by intact protein LCMS. The recombinant proteins selected were three E2 enzymes (UBCH5B, UBC13^K92A^, and UEV1A), two other TRIM protein substrate binding domains (TRIM21 PRYSPRY and TRIM2 NHL), and the catalytic OTU domains of three deubiquitinating enzymes containing active site cysteines (OTUD4, OTUD7B and ZRANB1). Pleasingly, 10 and 11 were observed to selectively label TRIM25 PRYSPRY, with minimal to no labelling of other ubiquitin system proteins (Fig. 3B). Unfortunately, the E1 enzyme and full-length TRIM25 could not be assessed in this panel as both proteins were too large for accurate deconvolution of intact protein mass spectra. Instead, an E1∼Ub loading assay was performed under reducing and non-reducing conditions^51^ to assess whether ubiquitin loading was inhibited by compounds 10 and 11. E1∼Ub loading was not inhibited, indicating that 10 and 11 did not covalently modify the E1 active site Cys (Fig. S6D, ESI†).

The selectivity and cellular interaction profiles of 10 and 11 were further characterised by chemoproteomics in live THP-1 cells. We employed an established competitive chemoproteomics workflow, using an iodoacetamide desthiobiotin (IA-DTB) probe,^52,53^ to assess compound selectivity (at 50 μM and 10 μM) and identify the site of modification on TRIM25. Comparison of IA-DTB labelled peptide intensities between compound-treated samples and DMSO controls enables compound engagement to be determined, reported as a fold change (FC) ratio. Compound 10 engaged TRIM25 in cells at the PRYSPRY domain at 50 μM, and we identified the peptide FTYC^498^SQVLGLHC^506^YK as the site of covalent modification. Although we detected two peptides for this sequence modified by the IA-DTB probe, we were unable to determine which of the two cysteine residues (Cys498 or Cys506) was modified from this experiment (Fig. 3C). At 50 μM, 50 other protein targets were also competed by compound 10, including VCPIP (a deubiquitinating enzyme) and DNMT1 (a DNA methyltransferase) among the most significant off-targets. Although 10 was not observed to be highly selective across the proteome and remains an early-stage covalent lead compound, TRIM25 was the only E3 ligase identified to be labelled by 10 in cells. Given the compound's size (370 Da) and minimal molecular recognition features (hydrophobic biphenyl ring system), 10 was considered a good starting point for further tool compound development. In contrast, compound 11 was not observed to engage TRIM25 or many other proteins in cells, despite having a similar *k*_inact_/*K*_I_ and GSH reactivity, suggesting poor cell permeability. Therefore, it was not progressed any further (Fig. S6E, ESI†).

To confirm the exact site of covalent modification, we performed mutagenesis on Cys498 and Cys506 of TRIM25 PRYSPRY, followed by incubation of recombinant mutants (10 μM) with compound 10 (50 μM) at 4 °C for 24 hours. Analysis by intact protein LCMS revealed Cys498 as the site of covalent modification by compound 10 (Fig. 3D). Multiple sequence alignment of all PRYSPRY-containing TRIM proteins showed that Cys498 is not conserved across the family (Fig. S7A, ESI†). Thus, the covalent targeting of Cys498 by compound 10 reveals a unique binding mode for TRIM25.

Next, structural characterisation of the protein–ligand complex provided insight into the binding mode of compound 10 and helped guide further compound elaboration. We solved the X-ray crystal structure of TRIM25 PRYSPRY in complex with compound 10 (PDB 9I0T, Table S1, ESI†), which showed 10 covalently bound to Cys498 within a network of hydrophobic aromatic residues. The biphenyl ring system of 10 forms π-stacking interactions with the aromatic side chains of Phe523, Phe554, Trp616, and Phe618 (Fig. 3E). Furthermore, the carbonyl of the Cys-linked acetamide bond of 10 acts as an H-bond acceptor for both the side chain hydroxyl and main chain –NH of Ser499, and the side chain indole amine of Trp616. The carbonyl of the diazepane amide linker also forms an H-bond interaction with the terminal guanidino group of Arg541 (Fig. 3F). Compared to the apo-TRIM25 PRYSPRY structure (PDB 6FLM),^15^ no significant conformational changes within the binding site occurred (Fig. S7B, ESI†). Additionally, we used ligand-based ^1^H-NMR to further validate the binding pose of compound 10, and confirmed its binding mode in solution (Fig. S7C, ESI†). Interestingly, structural overlay of TRIM25 PRYSPRY-compound 10 with the PRYSPRY domains of TRIM7, TRIM21 and TRIM58, for which small molecule ligands have been reported,^29–31^ showed that the ligands all bind at the same site on the PRYSPRY surface, which has also been suggested to be a substrate binding interface^31^ (Fig. S7D, ESI†). This may present an opportunity to target future PRYSPRY ligand discovery campaigns to this particular privileged ligand- and substrate-binding site.

### Heterobifunctional proximity-inducers enable *in vitro* targeted protein ubiquitination of a neosubstrate by TRIM25

**Fig. 4:**
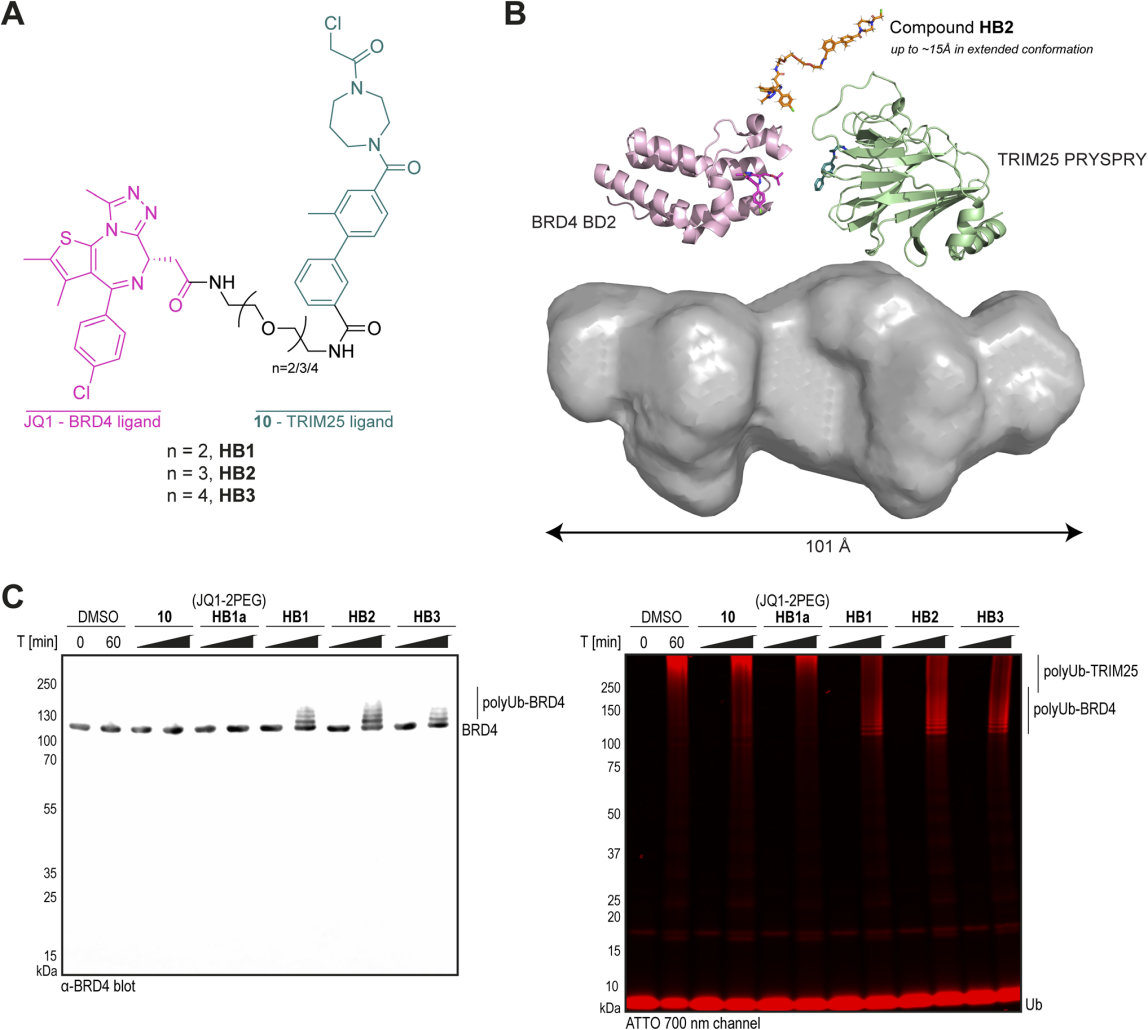
Design, SAXS and biochemical characterisation of heterobifunctional compounds for targeted protein ubiquitination of BRD4. (A) Chemical structures of heterobifunctional BRD4-recruiting compounds HB1, HB2 and HB3; (B) best DAMMIF *ab initio* molecular envelope for the TRIM25 PRYSPRY-HB2-BRD4 BD2 complex, with cartoon representations of BRD4 BD2-JQ1 (pale pink, PDB 3ONI),^54^ TRIM25 PRYSPRY-compound 10 (pale green, PDB 9I0T), and compound HB2 (orange); (C) *In vitro* targeted protein ubiquitination time course assay for BRD4, using His_10_-TRIM25 pre-treated with either DMSO or compounds. His_10_-TRIM25 (4 μM) was incubated with compounds (50 μM) or DMSO (1%) for 20 h at 4 °C. Excess unreacted compound was removed prior to the addition of E1 (0.5 μM), UBCH5B (2 μM), Ub (50 μM), Ub^ATTO^ (1 μM), and BRD4 (4 μM, no tags). Ubiquitination was initiated by the addition of ATP and performed for 60 min at 30 °C. The samples were analysed by SDS-PAGE, western blotting with α-BRD4, and scanning at 700 nm wavelength (for ATTO emission). Coomassie staining is shown in Fig. S8E, ESI.†

Using our TRIM25 PRYSPRY-compound 10 complex structure, we identified potential exit vectors for incorporation of 10 into proximity-inducing heterobifunctional molecules. The meta position on the pendant phenyl ring was determined to be both solvent accessible and synthetically tractable (Fig. 3G). Three heterobifunctional compounds (HB1, HB2, and HB3) were designed and synthesised with varying PEG linker lengths, incorporating 10, and the well-characterised BRD4 ligand, JQ1 (Fig. 4A and Scheme S4, ESI†).^54^ Compounds HB1–HB3 (50 μM) were incubated with the TRIM25 PRYSPRY domain (10 μM) at 4 °C for 20 hours, and labelling was analysed by intact protein LCMS (49%, 75% and 87%, respectively, Fig. S8A, ESI†). Although labelling was reduced compared to compound 10 alone (94%), the labelling observed confirmed that an exit vector at the *meta* position of the pendant phenyl ring was tolerated.

Next, we assessed *in vitro* ternary complex formation by recombinant protein pull-down, using isolated TRIM25 PRYSPRY and BRD4 BD2 domains. Ternary complex formation was observed for all three linker lengths (Fig. S8B, ESI†). However, an intermediate wash step after overnight incubation of the TRIM25 PRYSPRY domain (2 μM) with compounds HB1, HB2 or HB3 (50 μM) was required to remove excess unreacted heterobifunctional compound. This suggests the presence of a large hook effect, where, at the concentrations required for covalent labelling and without the intermediate wash step, binary complex interactions outcompeted ternary complex formation. This ternary complex formation, when carried out with the intermediate wash step, was also reproduced using full length TRIM25 and BRD4 proteins (Fig. S8C, ESI†). These long incubation times and the intermediate wash step indicate that compounds HB1–HB3 would require further optimisation for use in cellular assays. Incorporating a more potent binder of TRIM25 than compound 10 would reduce incubation times and likely remove the necessity for the intermediate wash step.

Additionally, we performed SPR to obtain the affinity of BRD4 BD2 for TRIM25 PRYSPRY pre-labelled with HB1–HB3. In the absence of a heterobifunctional compound, BRD4 BD2 demonstrated no affinity for TRIM25 PRYSPRY. Upon pre-labelling TRIM25 PRYSPRY with HB1–HB3, we observed strong binding of BRD4 BD2, with very little difference between each compound (*K*_D_ = 15 ± 1.9 nM, 20 ± 2.1 nM, and 22.5 ± 3.6 nM, respectively) (Fig. S8D, ESI†). Although ternary complex formation is a critical component of induced proximity pharmacology, incorporation of higher affinity ligands into heterobifunctional molecule design does not correlate directly with improved ubiquitination. To maintain optimal processivity, a balance between longevity of ternary complex association and rate of dissociation must be obtained, as well as optimal ternary complex conformation.^55^ Thus, we turned to structural biology to examine the ternary complex structure.

Unfortunately, our attempts to crystallise the ternary complex between TRIM25 PRYSPRY, BRD4 BD2 and any of the three heterobifunctional molecules were unsuccessful. Instead, we used small-angle X-ray scattering (SAXS) to gain structural information and assess the flexibility of the TRIM25 PRYPSRY-HB2-BRD4 BD2 ternary complex (Table S2, ESI†). SAXS analysis revealed that both TRIM25 PRYSPRY and BRD4 BD2 are monomeric in solution (Fig. S9A–D, ESI†). Upon ternary complex formation, we observed an elongated molecular shape, characterised by an expansion in both maximum particle dimension (*D*_max_) and radius of gyration (*R*_g_), without significant changes in the cross-section radius of gyration (*R*_c_) (Fig. S9E–G, ESI†). Flexibility analysis and DAMMIF *ab initio* modelling of the ternary complex supported this, and revealed a flexible, elongated envelope exceeding individual domain dimensions (Fig. 4B). This suggests that compound HB2 acts as a dynamic linker between TRIM25 PRYSPRY and BRD4 BD2 domains, with PEG groups providing rotational flexibility that can separate domains by up to ∼15 Å, precluding stable direct interdomain interactions in solution. Thus, our SAXS analysis reveals a ternary complex characterised by significant interdomain flexibility.

Finally, the targeted ubiquitination of BRD4 by TRIM25 was investigated, using the *in vitro* ubiquitination assay described earlier. Full-length TRIM25 (4 μM) was pre-labelled with either DMSO or compound HB1, HB2 or HB3 (50 μM) for 20 hours at 4 °C, and excess compound was removed with an intermediate wash step, and the TRIM25-compound complex was used as the E3 component in the *in vitro* ubiquitination assay. We observed heterobifunctional compound-induced ubiquitination of BRD4 (Fig. 4C and S8E, ESI†) with all three linker lengths. To confirm that this ubiquitination was induced by the formation of a ternary complex, we also performed this experiment using compound 10 and HB1a (JQ1-2PEG) alone, and no targeted ubiquitination of BRD4 was observed. Thus, although our biochemical, SPR, and SAXS data suggest a lack of positive cooperativity, we still observe proximity-induced targeted ubiquitination of BRD4 by TRIM25. Taken together, our results demonstrate that TRIM25, a previously unexplored E3 ligase in the induced proximity field, can be repurposed to ubiquitinate a neosubstrate *in vitro*.

## Conclusions

The discovery of small molecules targeting previously unliganded E3 ligases is needed to expand the repertoire of enzymes that can be repurposed for induced proximity modalities. In this work, we identified a novel covalent ligand for the E3 ligase, TRIM25, and demonstrated that it can be incorporated into heterobifunctional molecules capable of recruiting TRIM25 for proximity-induced *in vitro* ubiquitination of a neosubstrate.

Using covalent fragment screening coupled with a high-throughput chemistry direct-to-biology strategy to expedite the process of fragment optimisation, we identified two chloroacetamide ligands (compounds 10 and 11) that bind to the PRYSPRY domain of TRIM25. Further characterisation elucidated that these ligands enhance the auto-ubiquitination of TRIM25 *in vitro*, and selectively label the PRYSPRY domain of TRIM25 (at Cys498) over other ubiquitin system proteins *in vitro* and, for 10, in live THP-1 cells. A crystal structure of the TRIM25 PRYSPRY-compound 10 complex was solved at 1.8 Å resolution, which, to our knowledge, represents the first published structure of TRIM25 in complex with a small molecule. Structure-informed design guided the incorporation of compound 10 into proximity-inducing heterobifunctional molecules. We demonstrated heterobifunctional compound-induced ternary complex formation and targeted *in vitro* ubiquitination of BRD4 by TRIM25.

Further cellular studies to investigate the effects of neosubstrate recruitment and ubiquitination by TRIM25 will first require optimisation of compound 10. The current requirement for long incubation periods and removal of excess compound with HB1–HB3*in vitro* precludes the use of these compounds in cellular assays. Structure-based medicinal chemistry campaigns to improve the potency and selectivity of 10 will likely reduce the large hook effect observed with heterobifunctional compounds HB1–HB3, enabling induced ubiquitination at a range of concentrations, without an intermediate excess compound removal step. Investigating the function of optimised heterobifunctional molecules in a cellular context will help to uncover whether TRIM25 acts as a canonical degrader ligase or mediates a more complex signalling pathway. Orthogonally, optimised ligands could be further studied in a cellular context, either as inhibitors of reported TRIM25 substrates or as molecular glues to identify novel neosubstrates of TRIM25.

Overall, we have developed small molecules for TRIM25 and expanded the repertoire of liganded E3 ligases. Our results highlight the power in using covalent approaches for targeting shallow protein–protein interaction surfaces, and demonstrate that another E3 ligase within the TRIM family can be repurposed to direct ubiquitination towards a neosubstrate. As such, targeted ligand discovery for the recruitment of TRIM family proteins presents an attractive strategy for future induced proximity modalities.

## Author contributions

KAM wrote the manuscript and performed protein biochemistry, intact protein LCMS, HTC-D2B synthesis and screening, X-ray crystallography, synthetic chemistry, and biochemical and biophysical assays. DE performed crystallographic data processing and SAXS analysis. JP performed the initial covalent fragment screening experiment and carried out library management. DN, TW and TM performed chemoproteomics experiments and analysis. JP, JB and KR contributed to data analysis and discussion. JB and KR jointly supervised the study. All authors read and edited the final manuscript.

## Conflicts of interest

The authors declare no competing interests.

## Supplementary Material

SC-016-D5SC01540E-s001

SC-016-D5SC01540E-s002

SC-016-D5SC01540E-s003

## Data Availability

The X-ray crystal structure reported in this study has been deposited in the Protein Data Bank under accession code 9I0T. Additional information is also available in the attached Table S1, ESI.† The mass spectrometry proteomics data have been deposited to the ProteomeXchange Consortium (https://proteomecentral.proteomexchange.org) *via* the PRIDE partner repository^56^ with the dataset identifier PXD061182. Additional information is also available in the attached ESI Data S2.†

## References

[cit1] Damgaard R. B. (2021). The ubiquitin system: from cell signalling to disease biology and new therapeutic opportunities. Cell Death Differ..

[cit2] Swatek K. N., Komander D. (2016). Ubiquitin modifications. Cell Res..

[cit3] Komander D., Rape M. (2012). The ubiquitin code. Annu. Rev. Biochem..

[cit4] Berndsen C. E., Wolberger C. (2014). New insights into ubiquitin E3 ligase mechanism. Nat. Struct. Mol. Biol..

[cit5] Sakamoto K. M., Kim K. B., Kumagai A., Mercurio F., Crews C. M., Deshaies R. J. (2001). Protacs: chimeric molecules that target proteins to the Skp1–Cullin–F box complex for ubiquitination and degradation. Proc. Natl. Acad. Sci. U. S. A..

[cit6] Ahn G., Banik S. M., Miller C. L., Riley N. M., Cochran J. R., Bertozzi C. R. (2021). LYTACs that engage the asialoglycoprotein receptor for targeted protein degradation. Nat. Chem. Biol..

[cit7] Takahashi D., Moriyama J., Nakamura T., Miki E., Takahashi E., Sato A., Akaike T., Itto-Nakama K., Arimoto H. (2019). AUTACs: cargo-specific degraders using selective autophagy. Mol. Cell.

[cit8] Takahashi D., Ora T., Sasaki S., Ishii N., Tanaka T., Matsuda T., Ikeda M., Moriyama J., Cho N., Nara H., Maezaki H., Kamaura M., Shimokawa K., Arimoto H. (2023). Second-generation AUTACs for targeted autophagic degradation. J. Med. Chem..

[cit9] Henning N. J., Boike L., Spradlin J. N., Ward C. C., Liu G., Zhang E., Belcher B. P., Brittain S. M., Hesse M. J., Dovala D., McGregor L. M., Valdez Misiolek R., Plasschaert L. W., Rowlands D. J., Wang F., Frank A. O., Fuller D., Estes A. R., Randal K. L., Panidapu A., McKenna J. M., Tallarico J. A., Schirle M., Nomura D. K. (2022). Deubiquitinase-targeting chimeras for targeted protein stabilization. Nat. Chem. Biol..

[cit10] Chen P. H., Hu Z., An E., Okeke I., Zheng S., Luo X., Gong A., Jaime-Figueroa S., Crews C. M. (2021). Modulation of phosphoprotein activity by phosphorylation targeting chimeras (PhosTACs). ACS Chem. Biol..

[cit11] Belcher B. P., Ward C. C., Nomura D. K. (2023). Ligandability of E3 ligases for targeted protein degradation applications. Biochemistry.

[cit12] Ishida T., Ciulli A. (2021). E3 ligase ligands for PROTACs: how they were found and how to discover new ones. SLAS Discovery.

[cit13] Wegmann S., Meister C., Renz C., Yakoub G., Wollscheid H.-P., Takahashi D. T., Mikicic I., Beli P., Ulrich H. D. (2022). Linkage reprogramming by tailor-made E3s reveals polyubiquitin chain requirements in DNA-damage bypass. Mol. Cell.

[cit14] Yang E., Huang S., Jami-Alahmadi Y., McInerney G. M., Wohlschlegel J. A., Li M. M. H. (2022). Elucidation of TRIM25 ubiquitination targets involved in diverse cellular and antiviral processes. PLoS Pathog..

[cit15] Koliopoulos M. G., Lethier M., Van Der
Veen A. G., Haubrich K., Hennig J., Kowalinski E., Stevens R. V., Martin S. R., Reis e Sousa C., Cusack S., Rittinger K. (2018). Molecular mechanism of influenza A NS1-mediated TRIM25 recognition and inhibition. Nat. Commun..

[cit16] Álvarez L., Haubrich K., Iselin L., Gillioz L., Ruscica V., Lapouge K., Augsten S., Huppertz I., Choudhury N. R., Simon B., Masiewicz P., Lethier M., Cusack S., Rittinger K., Gabel F., Leitner A., Michlewski G., Hentze M. W., Allain F. H. T., Castello A., Hennig J. (2024). The molecular dissection of TRIM25's RNA-binding mechanism provides key insights into its antiviral activity. Nat. Commun..

[cit17] Gack M. U., Shin Y. C., Joo C. H., Urano T., Liang C., Sun L., Takeuchi O., Akira S., Chen Z., Inoue S., Jung J. U. (2007). TRIM25 RING-finger E3 ubiquitin ligase is essential for RIG-I-mediated antiviral activity. Nature.

[cit18] Sanchez J. G., Chiang J. J., Sparrer K. M. J., Alam S. L., Chi M., Roganowicz M. D., Sankaran B., Gack M. U., Pornillos O. (2016). Mechanism of TRIM25 catalytic activation in the antiviral RIG-I pathway. Cell Rep..

[cit19] Atkinson S. C., Heaton S. M., Audsley M. D., Kleifeld O., Borg N. A. (2021). TRIM25 and DEAD-Box RNA helicase DDX3X cooperate to regulate RIG-I-mediated antiviral immunity. Int. J. Mol. Sci..

[cit20] Li M. M. H., Lau Z., Cheung P., Aguilar E. G., Schneider W. M., Bozzacco L., Molina H., Buehler E., Takaoka A., Rice C. M., Felsenfeld D. P., MacDonald M. R. (2017). TRIM25 enhances the antiviral action of zinc-finger antiviral protein (ZAP). PLoS Pathog..

[cit21] Zheng X., Wang X., Tu F., Wang Q., Fan Z., Gao G. (2017). TRIM25 is required for the antiviral activity of zinc finger antiviral protein. J. Virol..

[cit22] Yang E., Nguyen L. A. P., Wisherop C. A., Kan R. L., Li M. M. H. (2022). The role of ZAP and TRIM25 RNA binding in restricting viral translation. Front. Cell. Infect. Microbiol..

[cit23] Choudhury N. R., Heikel G., Michlewski G. (2020). TRIM25 and its emerging RNA-binding roles in antiviral defense. Wiley Interdiscip. Rev.: RNA.

[cit24] Rahimi-Tesiye M., Zaersabet M., Salehiyeh S., Jafari S. Z. (2023). The role of TRIM25 in the occurrence and development of cancers and inflammatory diseases. Biochim. Biophys. Acta, Rev. Cancer.

[cit25] Martín-Vicente M., Medrano L. M., Resino S., García-Sastre A., Martínez I. (2017). TRIM25 in the regulation of the antiviral innate immunity. Front. Immunol..

[cit26] Tecalco-Cruz A. C., Abraham-Juárez M. J., Solleiro-Villavicencio H., Ramírez-Jarquín J. O. (2021). TRIM25: a central factor in breast cancer. World J. Clin. Oncol..

[cit27] Clift D., McEwan W. A., Labzin L. I., Konieczny V., Mogessie B., James L. C., Schuh M. (2017). A method for the acute and rapid degradation of endogenous proteins. Cell.

[cit28] Zeng J., Santos A. F., Mukadam A. S., Osswald M., Jacques D. A., Dickson C. F., McLaughlin S. H., Johnson C. M., Kiss L., Luptak J., Renner N., Vaysburd M., McEwan W. A., Morais-de-Sá E., Clift D., James L. C. (2021). Target-induced clustering activates Trim-Away of pathogens and proteins. Nat. Struct. Mol. Biol..

[cit29] Lu P., Cheng Y., Xue L., Ren X., Xu X., Chen C., Cao L., Li J., Wu Q., Sun S., Hou J., Jia W., Wang W., Ma Y., Jiang Z., Li C., Qi X., Huang N., Han T. (2024). Selective degradation of multimeric proteins by TRIM21-based molecular glue and PROTAC degraders. Cell.

[cit30] Muñoz Sosa C. J., Lenz C., Hamann A., Farges F., Dopfer J., Krämer A., Cherkashyna V., Tarnovskiy A., Moroz Y. S., Proschak E., Němec V., Müller S., Saxena K., Knapp S. (2024). A C-degron structure-based approach for the development of ligands targeting the E3 ligase TRIM7. ACS Chem. Biol..

[cit31] Hoegenauer K., An S., Axford J., Benander C., Bergsdorf C., Botsch J., Chau S., Fernández C., Gleim S., Hassiepen U., Hunziker J., Joly E., Keller A., Lopez Romero S., Maher R., Mangold A.-S., Mickanin C., Mihalic M., Neuner P., Patterson A. W., Perruccio F., Roggo S., Scesa J., Schröder M., Shkoza D., Thai B., Vulpetti A., Renatus M., Reece-Hoyes J. S. (2023). Discovery of ligands for TRIM58, a novel tissue-selective E3 ligase. ACS Med. Chem. Lett..

[cit32] McCarthy W. J., van der Zouwen A. J., Bush J. T., Rittinger K. (2024). Covalent fragment-based drug discovery for target tractability. Curr. Opin. Struct. Biol..

[cit33] Boike L., Henning N. J., Nomura D. K. (2022). Advances in covalent drug discovery. Nat. Rev. Drug Discov..

[cit34] Tao Y., Remillard D., Vinogradova E. V., Yokoyama M., Banchenko S., Schwefel D., Melillo B., Schreiber S. L., Zhang X., Cravatt B. F. (2022). Targeted protein degradation by electrophilic PROTACs that stereoselectively and site-specifically engage DCAF1. J. Am. Chem. Soc..

[cit35] Zhang X., Crowley V. M., Wucherpfennig T. G., Dix M. M., Cravatt B. F. (2019). Electrophilic PROTACs that degrade nuclear proteins by engaging DCAF16. Nat. Chem. Biol..

[cit36] Luo M., Spradlin J. N., Boike L., Tong B., Brittain S. M., McKenna J. M., Tallarico J. A., Schirle M., Maimone T. J., Nomura D. K. (2021). Chemoproteomics-enabled discovery of covalent RNF114-based degraders that mimic natural product function. Cell Chem. Biol..

[cit37] Henning N. J., Manford A. G., Spradlin J. N., Brittain S. M., Zhang E., McKenna J. M., Tallarico J. A., Schirle M., Rape M., Nomura D. K. (2022). Discovery of a covalent FEM1B recruiter for targeted protein degradation applications. J. Am. Chem. Soc..

[cit38] Zhang X., Luukkonen L. M., Eissler C. L., Crowley V. M., Yamashita Y., Schafroth M. A., Kikuchi S., Weinstein D. S., Symons K. T., Nordin B. E., Rodriguez J. L., Wucherpfennig T. G., Bauer L. G., Dix M. M., Stamos D., Kinsella T. M., Simon G. M., Baltgalvis K. A., Cravatt B. F. (2021). DCAF11 supports targeted protein degradation by electrophilic proteolysis-targeting chimeras. J. Am. Chem. Soc..

[cit39] Ward C. C., Kleinman J. I., Brittain S. M., Lee P. S., Chung C. Y. S., Kim K., Petri Y., Thomas J. R., Tallarico J. A., McKenna J. M., Schirle M., Nomura D. K. (2019). Covalent Ligand Screening uncovers a RNF4 E3 ligase recruiter for targeted protein degradation applications. ACS Chem. Biol..

[cit40] Jones L. H. (2024). Synthetic modification of protein surfaces to mediate induced-proximity pharmacology. RSC Med. Chem..

[cit41] Shah R. R., De Vita E., Sathyamurthi P. S., Conole D., Zhang X., Fellows E., Dickinson E. R., Fleites C. M., Queisser M. A., Harling J. D., Tate E. W. (2024). Structure-guided design and optimization of covalent VHL-targeted sulfonyl fluoride PROTACs. J. Med. Chem..

[cit42] Kiely-Collins H., Winter G. E., Bernardes G. J. L. (2021). The role of reversible and irreversible covalent chemistry in targeted protein degradation. Cell Chem. Biol..

[cit43] Grimster N. P. (2021). Covalent PROTACs: The best of both worlds?. R. Soc. Chem..

[cit44] Wilders H., Biggs G., Rowe S. M., Cawood E. E., Rendina A. R., Grant E. K., Riziotis I. G., Pettinger J., Fallon D. J., Skehel M., House D., Tomkinson N. C. O., Bush J. (2024). Expedited SARS-CoV-2 main protease inhibitor discovery through modular ‘direct-to-biology’ screening. Angew. Chem., Int. Ed..

[cit45] Vuorinen A., Kennedy C. R., McPhie K. A., McCarthy W., Pettinger J., Skehel J. M., House D., Bush J. T., Rittinger K. (2025). Enantioselective OTUD7B fragment discovery through chemoproteomics screening and high-throughput optimisation. Commun. Chem..

[cit46] Tanaka N., Ohno K., Niimi T., Moritomo A., Mori K., Orita M. (2009). Small-world phenomena in chemical library networks: application to fragment-based drug discovery. J. Chem. Inf. Model..

[cit47] Mason J., Wilders H., Fallon D. J., Thomas R. P., Bush J. T., Tomkinson N. C. O., Rianjongdee F. (2023). Automated LC-MS analysis and data extraction for high-throughput chemistry. Digital Discovery.

[cit48] Butterworth S., Cross D. A. E., Finlay M. R. V., Ward R. A., Waring M. J. (2017). The structure-guided discovery of osimertinib: the first U.S. FDA approved mutant selective inhibitor of EGFR T790M. Medchemcomm.

[cit49] Esposito D., Dudley-Fraser J., Garza-Garcia A., Rittinger K. (2022). Divergent self-association properties of paralogous proteins TRIM2 and TRIM3 regulate their E3 ligase activity. Nat. Commun..

[cit50] Koliopoulos M. G., Esposito D., Christodoulou E., Taylor I. A., Rittinger K. (2016). Functional role of TRIM E3 ligase oligomerization and regulation of catalytic activity. EMBO J..

[cit51] Stieglitz B., Rana R. R., Koliopoulos M. G., Morris-Davies A. C., Schaeffer V., Christodoulou E., Howell S., Brown N. R., Dikic I., Rittinger K. (2013). Structural basis for ligase-specific conjugation of linear ubiquitin chains by HOIP. Nature.

[cit52] Picco G., Rao Y., Al Saedi A., Lee Y., Vieira S. F., Bhosle S., May K., Herranz-Ors C., Walker S. J., Shenje R., Dincer C., Gibson F., Banerjee R., Hewitson Z., Werner T., Cottom J. E., Peng Y., Deng N., Zhang Y., Nartey E. N., Nickels L., Landis P., Conticelli D., McCarten K., Bush J., Sharma M., Lightfoot H., House D., Milford E., Grant E. K., Glogowski M. P., Wagner C. D., Bantscheff M., Rutkowska-Klute A., Zappacosta F., Pettinger J., Barthorpe S., Eberl H. C., Jones B. T., Schneck J. L., Murphy D. J., Voest E. E., Taygerly J. P., DeMartino M. P., Coelho M. A., Houseley J., Sharma G., Schwartz B., Garnett M. J. (2024). Novel WRN helicase inhibitors selectively target microsatellite-unstable cancer cells. Cancer Discov..

[cit53] Kuljanin M., Mitchell D. C., Schweppe D. K., Gikandi A. S., Nusinow D. P., Bulloch N. J., Vinogradova E. V., Wilson D. L., Kool E. T., Mancias J. D., Cravatt B. F., Gygi S. P. (2021). Reimagining high-throughput profiling of reactive cysteines for cell-based screening of large electrophile libraries. Nat. Biotechnol..

[cit54] Filippakopoulos P., Qi J., Picaud S., Shen Y., Smith W. B., Fedorov O., Morse E. M., Keates T., Hickman T. T., Felletar I., Philpott M., Munro S., McKeown M. R., Wang Y., Christie A. L., West N., Cameron M. J., Schwartz B., Heightman T. D., La Thangue N., French C. A., Wiest O., Kung A. L., Knapp S., Bradner J. E. (2010). Selective inhibition of BET bromodomains. Nature.

[cit55] Crowe C. , Nakasone M. A., Chandler S., Craigon C., Sathe G., Tatham M. H., Makukhin N., Hay R. T. and Ciulli A., Mechanism of Degrader-Targeted Protein Ubiquitinability, 2024, vol. 10 10.1126/sciadv.ado6492 PMC11468923 39392888

[cit56] Perez-Riverol Y., Bai J., Bandla C., García-Seisdedos D., Hewapathirana S., Kamatchinathan S., Kundu D. J., Prakash A., Frericks-Zipper A., Eisenacher M., Walzer M., Wang S., Brazma A., Vizcaíno J. A. (2022). The PRIDE database resources in 2022: a hub for mass spectrometry-based proteomics evidences. Nucleic Acids Res..

