# Supplementary material for: Discovery and optimisation of a covalent ligand for TRIM25 and its application to targeted protein ubiquitination
Source: Chem Sci. 2025 May 12;16(23):10432–43. doi: 10.1039/d5sc01540e (PMC12067093; doi:10.1039/d5sc01540e)
Supplement: SC-016-D5SC01540E-s003 [file SC-016-D5SC01540E-s003.pdf]

Supplementary Information (SI) for Chemical Science.
This journal is © The Royal Society of Chemistry 2025

Discovery and optimisation of a covalent ligand for TRIM25 and its application to targeted protein recruitment modalities

Katherine A. McPhie[1], Diego Esposito[1], Jonathan Pettinger[2], Daniel Norman[3], Thilo Werner[4], Toby Mathieson[4], Jacob T. Bush[2], Katrin Rittinger[1]*

[1] Molecular Structure of Cell Signalling Laboratory, The Francis Crick Institute, 1 Midland Road, London, NW1 1AT, UK.

[2] Crick-GSK Biomedical LinkLabs, GSK, Gunnels Wood Road, Stevenage, Hertfordshire, SG1 2NY, UK.

[3] Chemical Biology, GSK, Gunnels Wood Road, Stevenage, Hertfordshire, SG1 2NY, UK

[4] Cellzome GmbH, a GSK Company, Meyerhofstrasse 1, 69117 Heidelberg, Germany

* Corresponding author

Contents

S1. Supplementary Figures **3**

Supplementary Figure 1 *Fragment screening supplementary data.* 3

Supplementary Figure 2 *Counter fragment screen against TRIM21 PRYSPRY.* 4

Supplementary Figure 3 *Fragment hit kinetic and reactivity characterisation.* 5

Supplementary Figure 4 *HTC-D2B library design and deconvoluted intact protein LCMS spectra.* 6

Supplementary Figure 5 *Purified optimised compound kinetic characterisation.* 8

Supplementary Figure 6 *Further biochemical characterisation of purified compounds,* ***10*** *–* ***12****.* 10

Supplementary Figure 7 *Further structural characterisation of compound* ***10****.* 11

Supplementary Figure 8 *Biochemical characterisation of heterobifunctional compounds,* ***HB1****,* ***HB2*** *and* ***HB3****.* 12

Supplementary Figure 9 *SAXS data for TRIM25 PRYSPRY, BRD4 BD2 and TRIM25 PRYSPRY-****HB2****-BRD4 BD2 complex.* 14

S2. Supplementary Tables **15**

Supplementary Table 1 *X-ray crystallography data collection and refinement statistics for TRIM25 PRYSPRY-compound **10** complex (PDB 9I0T).* 15

Supplementary Table 2 *SAXS parameters and structure statistics for TRIM25 PRYSPRY, BRD4 BD2 and TRIM25 PRYSPRY-**HB2**-BRD4 BD2 complex.* 16

S3. Supplementary Schemes 17

Supplementary Scheme 1 *Synthesis of compound **10*** 17

Supplementary Scheme 2 *Synthesis of compound **11*** 17

Supplementary Scheme 3 *Synthesis of compound **12*** 17

Supplementary Scheme 4 *Synthesis of heterobifunctional compounds, **HB1** – **HB3*** 18

S4. Supplementary Data 19

Supplementary Data 1 *Chloroacetamide fragment library* 19

Supplementary Data 2 *Chemoproteomics processed data for IA-DTB cellular target identification* 19

S4. Experimental 20

Recombinant protein expression and purification 20

Intact protein LCMS 21

High-throughput chemistry direct-to-biology (HTC-D2B) 23

Kinetic characterisation 25

Glutathione reactivity assay 26

Recombinant protein ubiquitination assays 26

Cell treatment and IA-DTB chemoproteomics 27

X-ray crystallography 29

Ligand-based ^{1}H NMR 29

Recombinant protein ternary complex pull-down assays 29

Ternary complex SPR 30

Small-angle X-ray scattering (SAXS) 30

Synthetic Chemistry 31

S1. Supplementary Figures

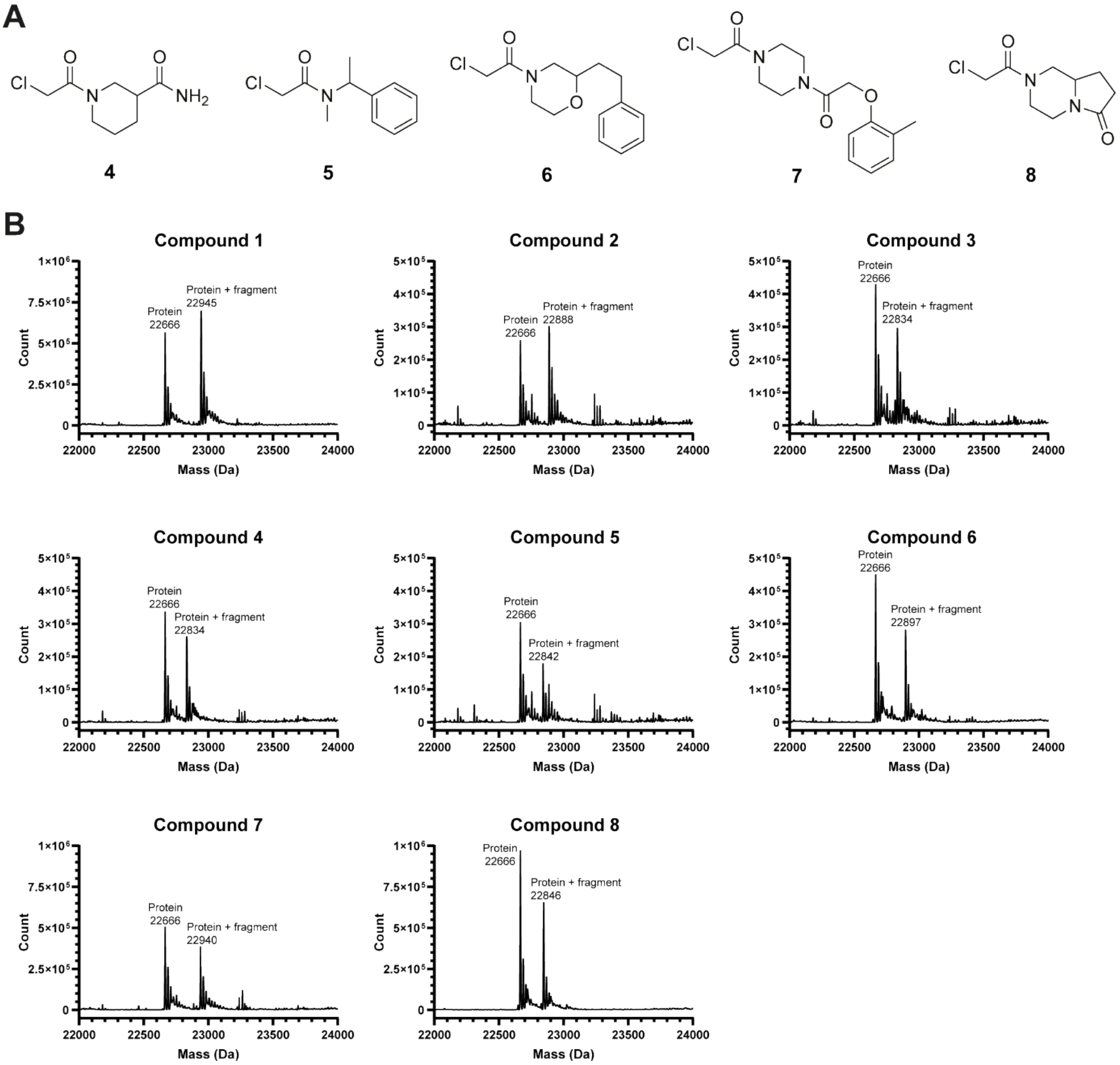

Supplementary Figure 1 Fragment screening supplementary data.

*A) Chemical structures of remaining five fragment hits **4 – 8**; B) Representative examples of deconvoluted intact protein LCMS spectra for fragment hits **1 – 8** (50 μM) in the fragment screen against recombinant TRIM25 PRYSPRY (0.25 μM) .*

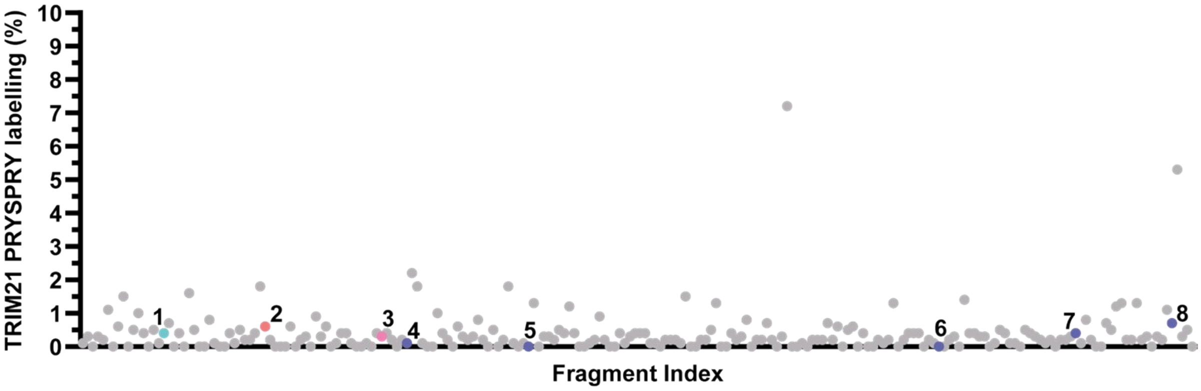

Supplementary Figure 2 Counter fragment screen against TRIM21 PRYSPRY.

Summary of covalent fragment screen by intact protein LCMS. % labelling of 221 chloroacetamides (50 µM) against TRIM21 PRYSPRY (0.5 µM) at 4 °C for 24 h.

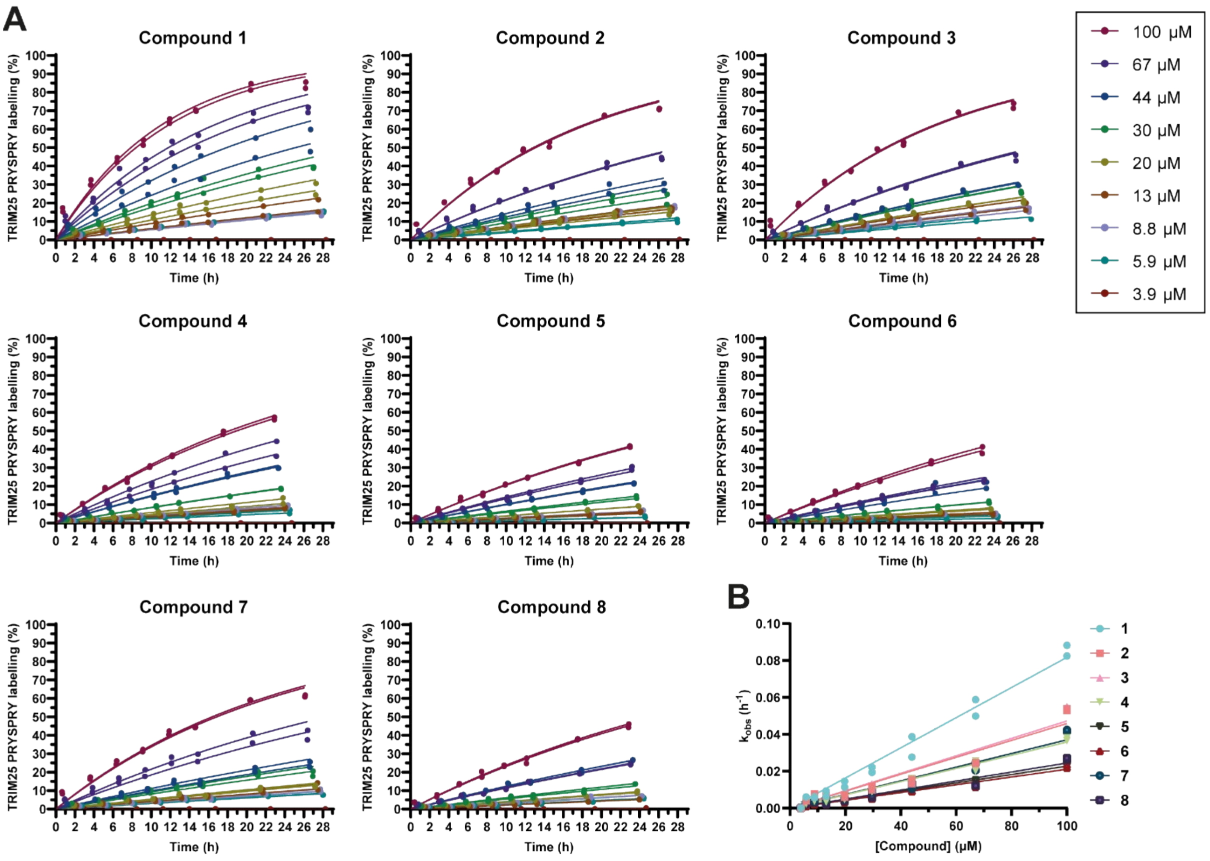

C

Compound	TRIM25 PRYSPRY labelling (%)	k_{inact}/K_I ($M^{-1}s^{-1}$)	GSH $t_{1/2}$ (h)
1	55	0.23 ± 0.006	1.7
2	50	0.13 ± 0.009	1.1
3	39	0.13 ± 0.010	N. D.
4	42	0.099 ± 0.003	N. D.
5	35	0.063 ± 0.001	N. D.
6	38	0.058 ± 0.003	N. D.
7	43	0.10 ± 0.006	N. D.
8	38	0.068 ± 0.004	N. D.

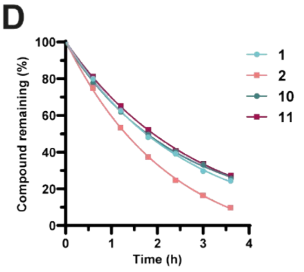

Supplementary Figure 3 Fragment hit kinetic and reactivity characterisation.

A) Time courses of fragment labelling (100 – 3.9 μM) against TRIM25 PRYSPRY (0.5 μM), performed in technical duplicate. % labelling was plotted against time, and curves were fitted separately for each replicate using one-phase association, with constraints Y0 = 0 and plateau = 100; B) Pseudo-first order rate constant values (k_{obs}) from time course labelling graphs were plotted against concentration, in duplicate, and fitted using straight line fit with constraints Yintercept = 0. Data are presented as mean ± SE of fit, n = 2. Slope of fit gives reported k_{inact}/K_I values; C) Table of TRIM25 PRYSPRY % labelling from fragment screen (50 μM), k_{inact}/K_I values ($M^{-1}s^{-1}$), and GSH assay $t_{1/2}$ values (h) (in the presence of 4 mM GSH). GSH $t_{1/2}$ values where only obtained for hit fragments selected for HTC-D2B progression, however ***3*** *did not have a chromophore, so a $t_{1/2}$ value could not be determined; D) GSH assay $t_{1/2}$ decay plots for original fragment hits* ***1*** *and* ***2****, and optimised HTC-D2B purified compounds* ***10*** *and* ***11****.*

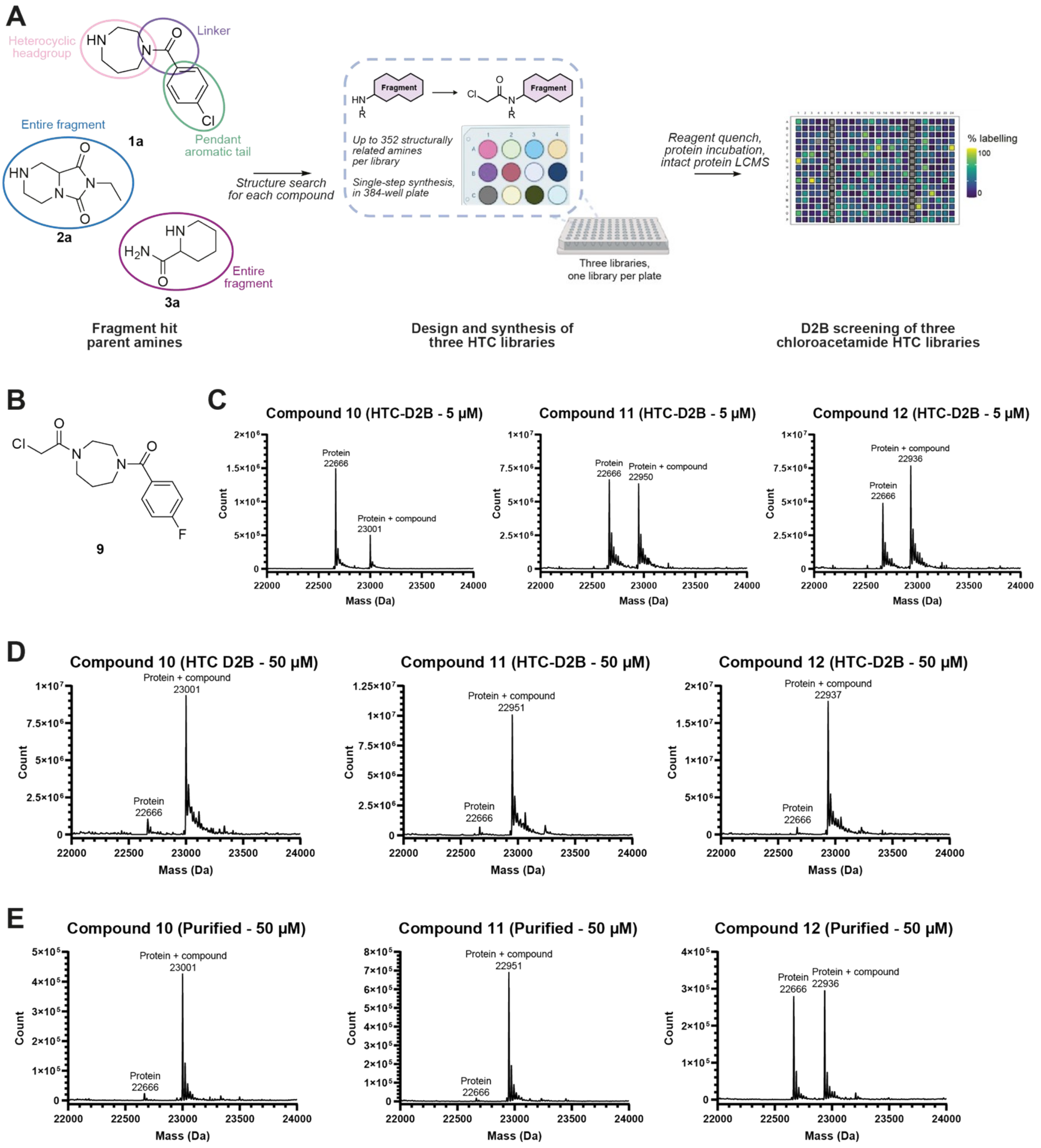

Supplementary Figure 4 HTC-D2B library design and deconvoluted intact protein LCMS spectra.

*A) HTC-D2B workflow for fragment hit parent amines (**1a** – **3a**). Three separate HTC-D2B hit expansion libraries were designed based on **1a**, **2a** and **3a**. For each parent amine, a Tanimoto-based similarity search was performed, filtering for readily available amines with a molecular weight between 110 – 350 Da. This resulted in a curated library of 83 parent amines based on fragment **1** (HTC-D2B plate 1, designed by separating the fragment into three distinct areas), 212 parent amines based on fragment **2** (HTC-D2B plate 2, designed based on structural similarity of the entire fragment), and 186 parent amines based on fragment **3** (HTC-D2B plate 3, designed based on structural similarity of the entire fragment). Installation of the chloroacetamide electrophile was performed in situ for all three libraries, and following a hydroxylamine quench, the three libraries were incubated with TRIM25 PRYSPRY (0.5 µM) at 4 °C for 24 hours, and screened by intact protein LCMS. Created in BioRender. McPhie, K. (2025) https://BioRender.com/e83b448; B) Chemical structure of HTC-D2B compound **9**, an*

*analogue of **1** synthesised in HTC-D2B plate 1 library; C) Representative examples of deconvoluted intact protein LCMS spectra for HTC-D2B hit compounds **10** – **12** at 5 μM and; D) at 50 μM in the HTC-D2B screen against recombinant TRIM25 PRYSPRY (0.5 μM); E) Representative examples of deconvoluted intact protein LCMS spectra for purified hit compounds **10** – **12** at 50 μM against recombinant TRIM25 PRYSPRY (10 μM).*

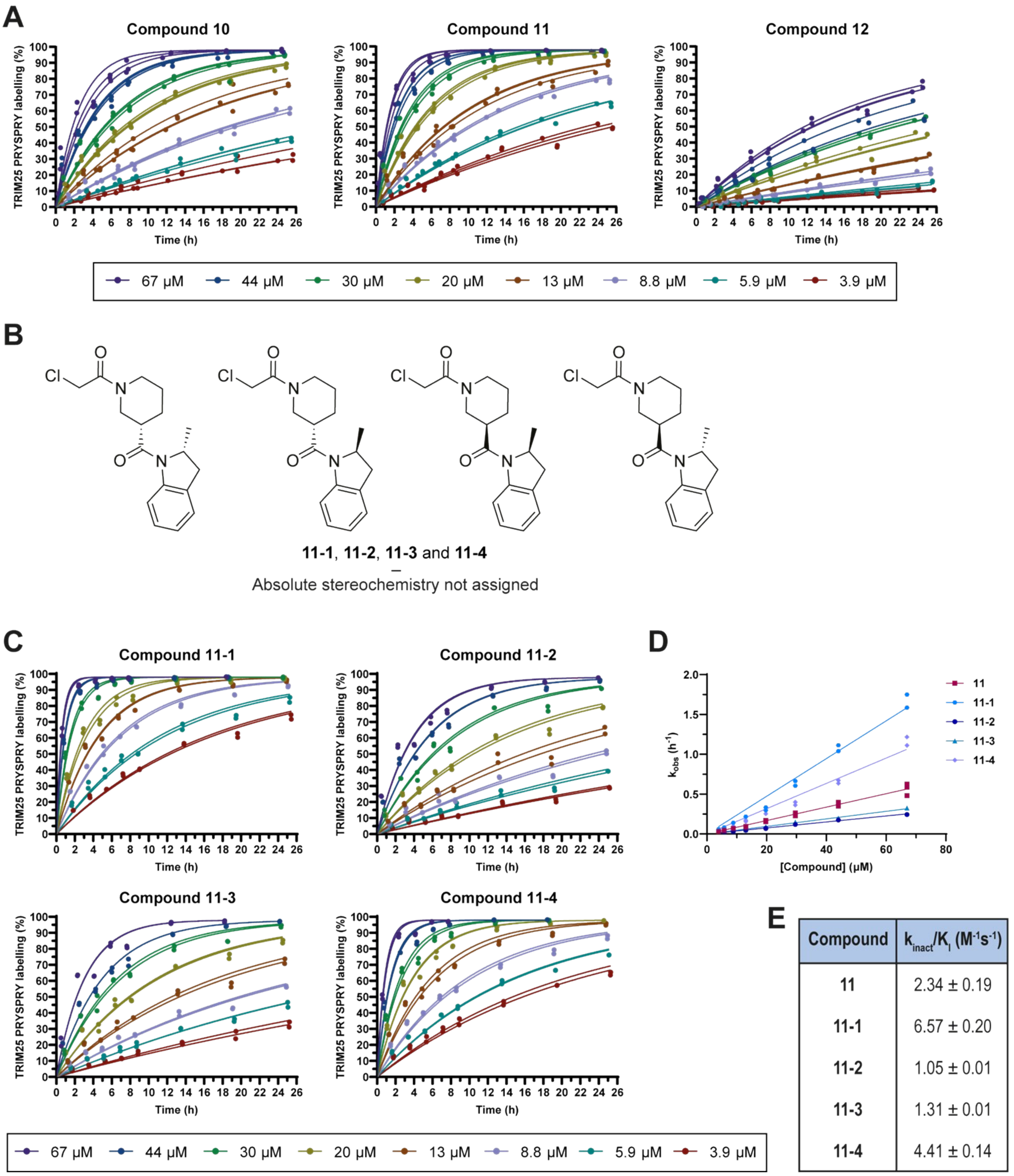

Compound	k_{inact}/K_I ($M^{-1}s^{-1}$)
11	2.34 ± 0.19
11-1	6.57 ± 0.20
11-2	1.05 ± 0.01
11-3	1.31 ± 0.01
11-4	4.41 ± 0.14

Supplementary Figure 5 Purified optimised compound kinetic characterisation.

*A) Time courses of compound labelling (67 – 3.9 μM) against TRIM25 PRYSPRY, performed in technical triplicate. % labelling was plotted against time, and curves were fitted separately for each replicate using one-phase association, with constraints Y0 = 0 and plateau = 100; B) Chemical structures of diastereomers **11-1** to **11-4**. Absolute stereochemistry is not assigned, however **11-1** and **11-2** are enantiomers, and **11-3** and **11-4** are enantiomers, based on LCMS and NMR; C) Time courses of compound labelling (67 – 3.9 μM) against TRIM25 PRYSPRY, performed in technical duplicate. % labelling was plotted against time, and curves were fitted separately for each replicate using one-phase association, with constraints Y0 = 0 and plateau = 100; D) Pseudo-first order rate constant values (k_{obs}) from time course labelling graphs were plotted against concentration, in duplicate (triplicate for **11**), and fitted using straight line fit with constraints Yintercept = 0. Data are presented as mean ± SE of fit, n = 2*

*(mean ± SD, n = 3 for **11**). Slope of fit gives reported k_{inact}/K_I values; E) Table of reported % labelling of purified compounds (50 µM) against TRIM25 PRYSPRY, and k_{inact}/K_I values ($M^{-1}s^{-1}$).*

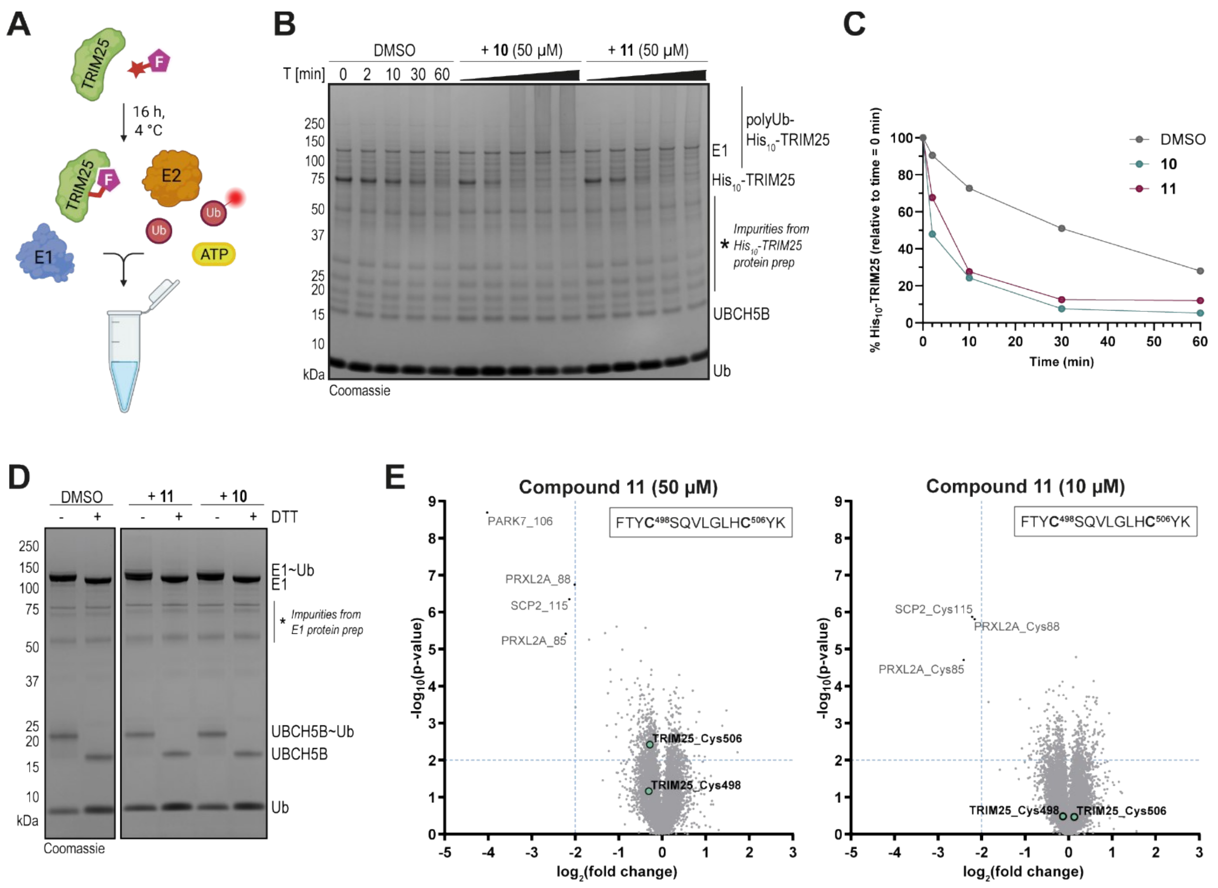

Supplementary Figure 6 Further biochemical characterisation of purified compounds, 10 – 12.

*A) Schematic of auto-ubiquitination assay, with pre-labelled full-length TRIM25. Created in BioRender. McPhie, K. (2025) https://BioRender.com/q86g447; B) Auto-ubiquitination time course assays with TRIM25 pre-treated with either DMSO or compound **10** or **11**. TRIM25 (4 μM) was incubated with compounds (50 μM) or DMSO (1%) for 16 h at 4 °C, before addition of E1 (0.2 μM), UBCH5B (2 μM), Ub (50 μM), Ub^{ATTO} (1 μM). The assay was initiated by the addition of ATP, and performed for 60 min at 30 °C. Time point 0 was taken before the addition of ATP. Samples were analysed by SDS-PAGE, with Coomassie staining and scanning at 700 nm wavelength (for ATTO emission). ATTO emission is shown in Figure 3A; C) Quantification of His_{10}-TRIM25 (which has not been ubiquitinated) where % His_{10}-TRIM25 (relative to time = 0 min) is plotted vs reaction time. Data are presented as n = 1; D) E1~Ub loading assay with E1 pre-treated with either DMSO or compound **10** or **11**. E1 (2 μM) was incubated with compounds (100 μM) or DMSO (1%) for 30 min at RT, before addition of UBCH5B (2 μM) and Ub (10 μM). The assay was initiated by the addition of ATP (1 mM), and performed for 5 min at 30 °C. After 5 min, samples were treated with either loading dye, or loading dye with DTT, and analysed by SDS-PAGE, with Coomassie staining; E) Cellular target identification for **11** (50 μM, left, and 10 μM, right, 4 h incubation at 37 °C in live THP-1 cells) using an iodoacetamide desthiobiotin (IA-DTB) probe-based competitive profiling approach. Significantly competed sites Cys498 and Cys506 on TRIM25 peptide, FTYC^{498}SQVLGLHC^{506}YK, are highlighted in teal. Top five significantly competed off-target Cys sites are highlighted in grey.*

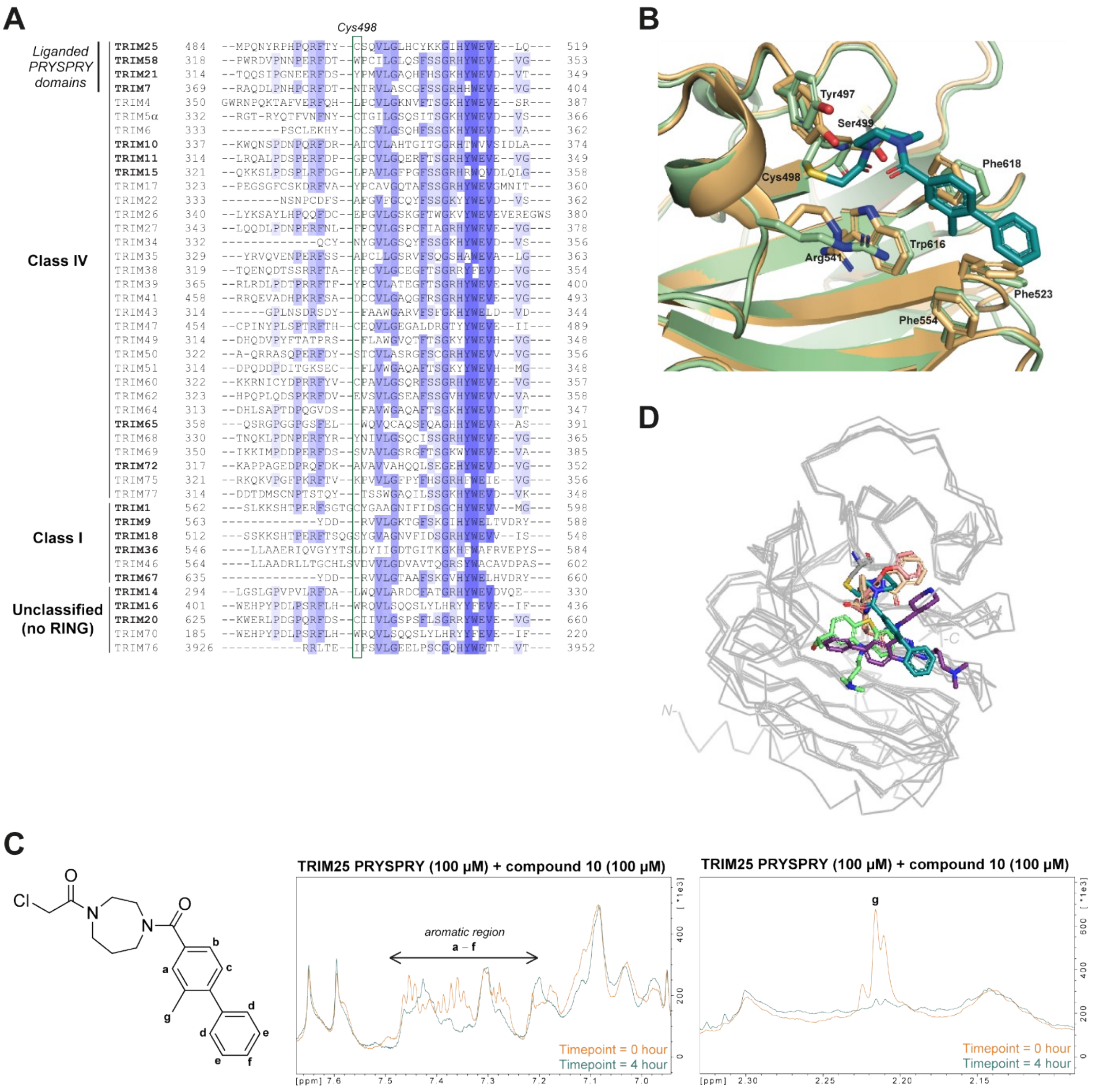

Supplementary Figure 7 Further structural characterisation of compound 10.

*A) Multiple sequence alignment of 44 PRYSPRY domain-containing TRIM proteins, across the Cys498-containing region of the PRYSPRY domain. Sequence conservation highlighted in blue, and TRIM25 Cys498 alignment highlighted in green box. Alignment was carried out using Clustal in Jalview v2.11.4.1[1]; B) Structural alignment of apo-TRIM25 PRYSPRY (orange, PDB 6FLM) with TRIM25 PRYSPRY-compound **10** complex (pale green protein, teal ligand, PDB 9I0T); C) Ligand-based ^{1}H-NMR of compound **10.** TRIM25 PRYSPRY (100 µM) was incubated with compound **10** (100 µM) and ^{1}H-NMR was recorded upon addition at 0 h (orange) and after 4 h incubation at RT (teal); D) Structural alignment of PRYSPRY-ligand crystal structures (all proteins grey) for: TRIM25 PRYSPRY-compound **10** complex (PDB 9I0T, teal ligand), TRIM58 PRYSPRY-**TRIM-473** complex (PDB 8PD6, purple ligand)[2], TRIM21 PRYSPRY-**(S)-ACE-OH** complex (PDB 8Y59, green ligand)[3] and TRIM7 PRYSPRY-ligand complexes (PDB 8R5B and 8R5C, pink and beige ligands).[4]*

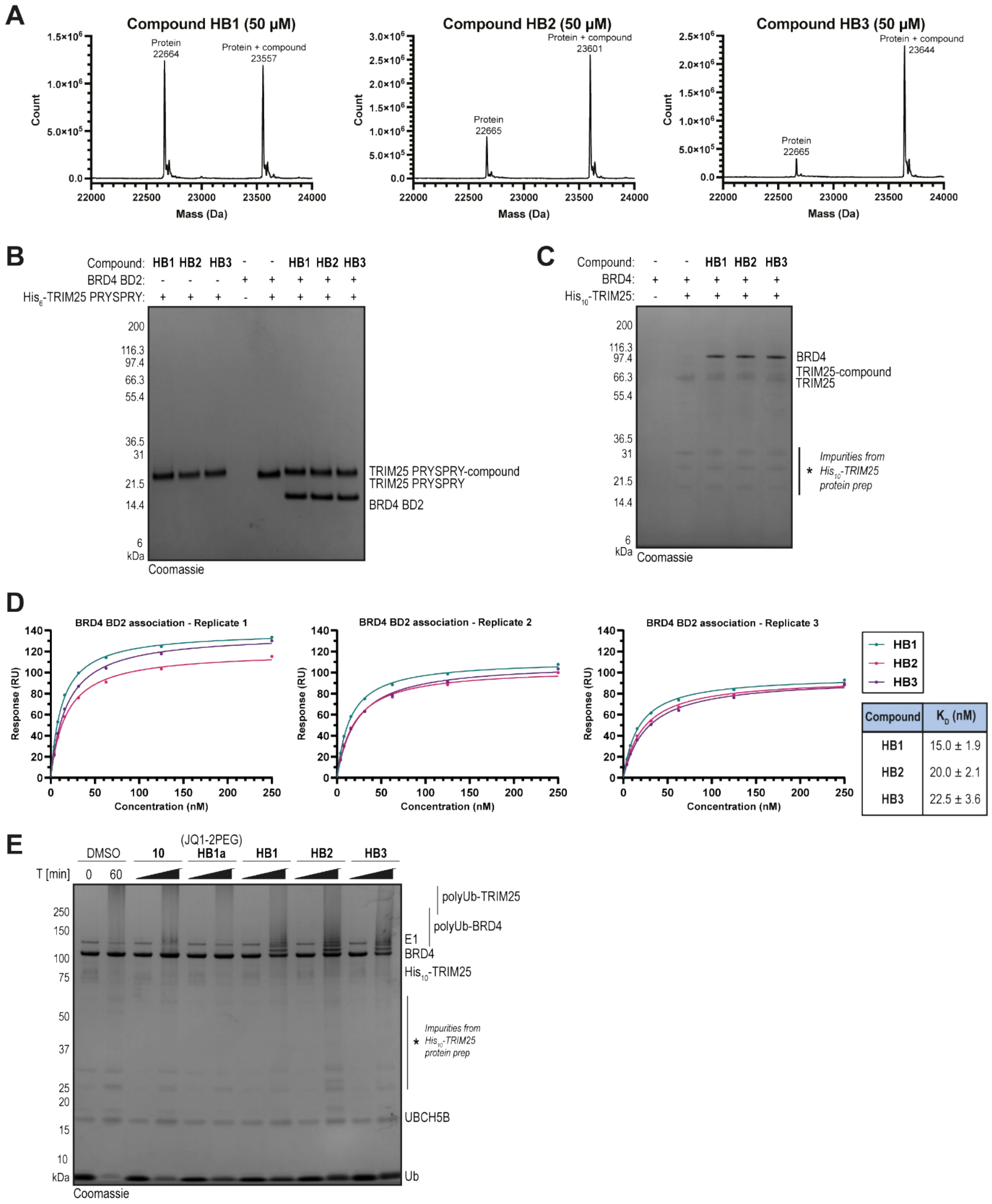

Supplementary Figure 8 Biochemical characterisation of heterobifunctional compounds, HB1, HB2 and HB3.

*A) Deconvoluted intact protein LCMS spectra for compounds **HB1** – **HB3** at 50 µM against recombinant TRIM25 PRYSPRY (10 µM); B) Recombinant protein pull-down. His$_6$-TRIM25 PRYSPRY (4 µM) pre-labelled with either DMSO, **HB1**, **HB2** or **HB3** (50 µM, incubation at 4 °C for 20 h), with excess unreacted compound removed prior to incubation with BRD4 BD2 (3 µM, no tags). MW shifts are visible for TRIM25-compound complex samples for different MWs of **HB1**, **HB2** or **HB3**; C) Recombinant protein pull-down. His$_{10}$-TRIM25 (4 µM) pre-labelled with either DMSO, **HB1**, **HB2** or **HB3** (50 µM, incubation at 4 °C for 20 h), with excess unreacted compound removed prior to incubation with BRD4 (3 µM, no tags); D) SPR data.*

*Pre-incubated TRIM25 PRYSPRY-AviTag (10 µM) with compounds **HB1**, **HB2** or **HB3** (100 µM, 2% DMSO), with incubation at 4 °C for 18 h, immobilised on streptavidin SPR chip. Binding response (RU) of analyte BRD4 BD2 shown with either TRIM25 PRYSPRY-compound **HB1** (2-PEG, teal), TRIM25 PRYSPRY-compound **HB2** (3-PEG, pink), and TRIM25 PRYSPRY-compound **HB3** (4-PEG, purple). K_D values are reported as mean ± SD, n = 3; E) In vitro targeted protein ubiquitination time course assay for BRD4, using His_{10}-TRIM25 pre-treated with either DMSO or compounds. His_{10}-TRIM25 (4 µM) was incubated with compounds (50 µM) or DMSO (1%) for 20 h at 4 °C. Excess unreacted compound was removed prior to addition of E1 (0.5 µM), UBCH5B (2 µM), Ub (50 µM), Ub^{ATTO} (1 µM), and BRD4 (4 µM, no tags). The assay was initiated by the addition of ATP, and performed for 60 min at 30 °C. Samples were analysed by SDS-PAGE, with Coomassie staining. Scanning at 700 nm wavelength (for ATTO emission), and western blot with α-BRD4 shown in Figure 4C.*

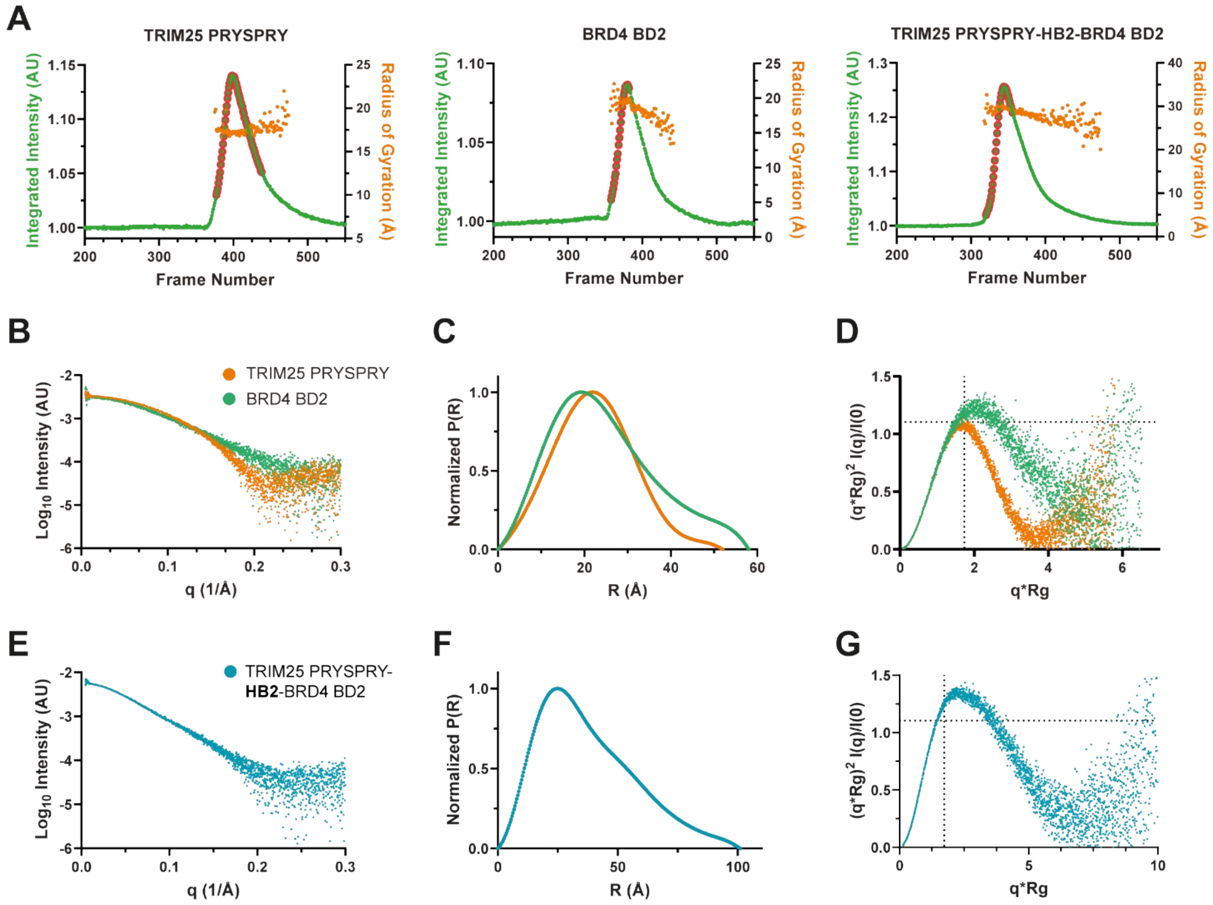

Supplementary Figure 9 SAXS data for TRIM25 PRYSPRY, BRD4 BD2 and TRIM25 PRYSPRY-HB2-BRD4 BD2 complex.

*A) Integrated intensity (green) of recorded small angle X-ray scattering (SAXS) as a function of frames recorded off a Superdex 75 3.2 x 300 column at 0.075 ml/min flow rate. Plotted as orange dots are the values of the derived radii of gyration for the background subtracted recorded profiles. Highlighted are the frames used for the analysis; B) X-ray scattering profiles for TRIM25 PRYSPRY (orange) and BRD4 BD2 (green); C) Normalised pair-distribution functions P(R) for TRIM25 PRYSPRY (orange) and BRD4 BD2 (green); D) Kratky plots for TRIM25 PRYSPRY (orange) and BRD4 BD2 (green). TRIM25 PRYSPRY experimental data matched theoretical predictions ($\chi 2 = 1.4$) suggesting domain is largely globular, however, BRD4 showed deviations consistent with conformational flexibility; E) X-ray scattering profile for TRIM25 PRYSPRY-**HB2**-BRD4 BD2 complex; F) Normalised pair-distribution function P(R) for TRIM25 PRYSPRY-**HB2**-BRD4 BD2 complex; G) Kratky plot for TRIM25 PRYSPRY-**HB2**-BRD4 BD2 complex, indicating flexibility exhibited by the upward trend at high q*Rg values.*

S2. Supplementary Tables

Supplementary Table 1 X-ray crystallography data collection and refinement statistics for TRIM25 PRYSPRY-compound 10 complex (PDB 9I0T).

Data for highest resolution shell given in parentheses.

Crystal	TRIM25 PRYSPRY-compound 10
Wavelength (Å)	0.9537
Resolution (Å)	49.00 – 1.80 (1.90 – 1.80)
Space group	P 21 21 21
Cell dimensions	
a, b, c (Å)	44.36, 68.78, 69.83
α, β, γ (°)	90.0, 90.0, 90.0
Total reflections	239762 (19829)
Unique reflections	20050 (2585)
Multiplicity	12.0 (7.7)
Completeness (%)	97.64 (89.86)
Mean I/σ(I)	20.98 (2.24)
Wilson B-factor	20.21
R-meas	0.0623 (0.3213)
R-pim	0.0175 (0.109)
$CC_{1/2}$	1.0 (0.974)
CC*	1.0 (0.993)
**Refinement**	
Reflections used in refinement	19922 (2569)
Reflections used for R_{free}	971 (129)
R_{work}	0.2215 (0.2329)
R_{free}	0.2449 (0.3059)
Number of non-hydrogen atoms	1692
Macromolecules	1583
Ligands	25
Solvent	84
Protein residues	197
RMS (bonds) (Å)	0.012
RMS (angles) (°)	0.91
Ramachandran favoured (%)	98.46
Ramachandran allowed (%)	1.54
Ramachandran outliers (%)	0.0
Rotamer outliers (%)	0.58
Clashscore	3.47
Average B-factor (Å^2)	24.01
Macromolecules (Å^2)	23.90
Ligands (Å^2)	26.02
Solvent (Å^2)	25.47

Supplementary Table 2 SAXS parameters and structure statistics for TRIM25 PRYSPRY, BRD4 BD2 and TRIM25 PRYSPRY-HB2-BRD4 BD2 complex.

**Data collection**			
Beamline	B21 at Diamond		
Wavelength	0.9464 Å		
q range (Å^{-1})	0.0045 – 0.34		
Detector	EigerX 4M (Dectris)		
Beamsize	< 75 µm		
Energy	13.1 keV		
Column	Superdex 75 3.2 x 300 (total volume = 2.4 mL)		
Flow rate (mL/min)	0.075		
Temperature (°C)	15		
**Sample details**	TRIM25 PRYSPRY	BRD4 BD2	TRIM25 PRYSPRY-**HB2**-BRD4 BD2
Sample volume (µL)	60	60	60
Sample concentration (mg/mL)	3.6	2.4	6.1
**Structural parameters**			
**Reciprocal Space**			
Rg (Å) Guinier	17.1	19.2	30.0
I(0) (cm^{-1})	0.003356	0.003178	0.005639
qRg limit	1.30	1.30	1.30
**Real Space**			
Rg (Å) P(R)	17.1 ± 0.02	19.2 ± 0.04	30.0 ± 0.05
I(0) (cm^{-1})	0.003356 ± 0.000002	0.003178 ± 0.000004	0.005639 ± 0.000006
Rc (Å)	12.3	9.6	15.7
Dmax (Å)	52	58	101
Porod volume (Å^3)	33275	22728	59807
**Molecular mass determination**			
Theoretical MW (kDa)	22.7	14.8	38.4
DATPOROD MW (kDa) (Vp/1.6)	20.8	14.2	37.4
**TRIM25 PRYSPRY-HB2-BRD4 BD2 ab-initio structural modelling**			
**Dammif**			
Number of calculated envelopes		25	
Number of final accepted envelopes		24	
Normalized Spatial Discrepancy		0.93 ± 0.09	
χ^2 (all)		1.519 ± 0.002	
χ^2 (best)		1.515	
**Data analysis software**			
Primary Data Reduction & Processing		Primus & Scatter	
Ab-initio modelling		Dammif	
Computation of model intensities		Crysol	
3D graphics representation		PyMol	

S3. Supplementary Schemes

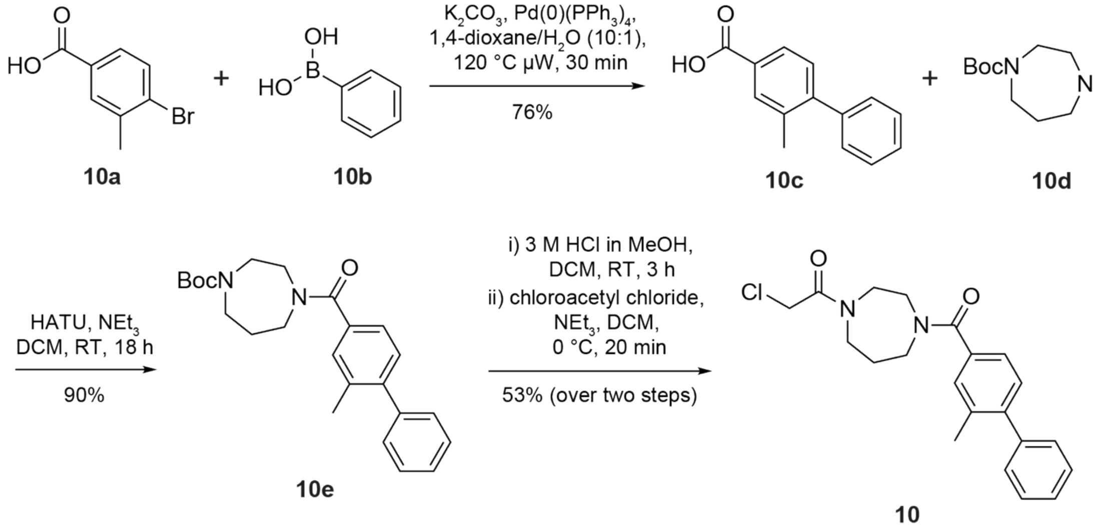

Supplementary Scheme 1 Synthesis of compound 10

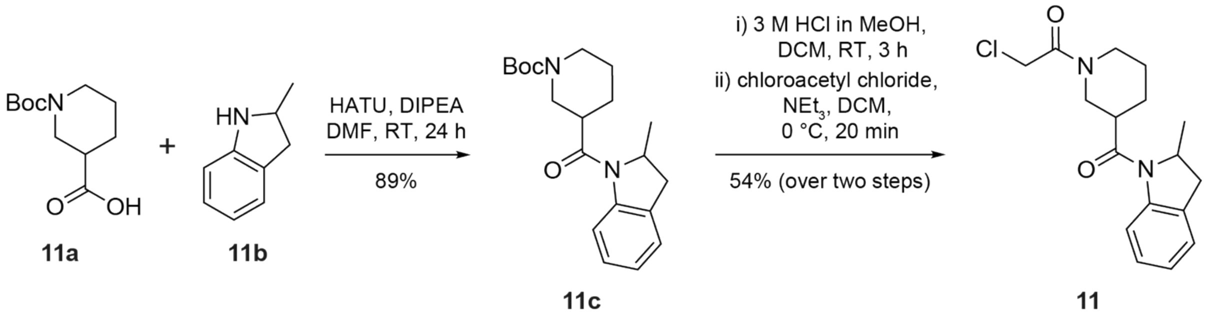

Supplementary Scheme 2 Synthesis of compound 11

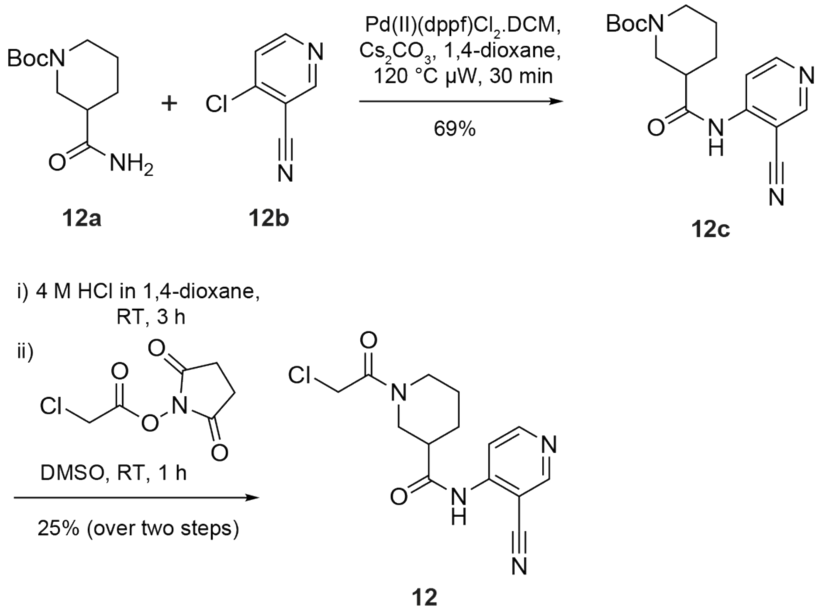

Supplementary Scheme 3 Synthesis of compound 12

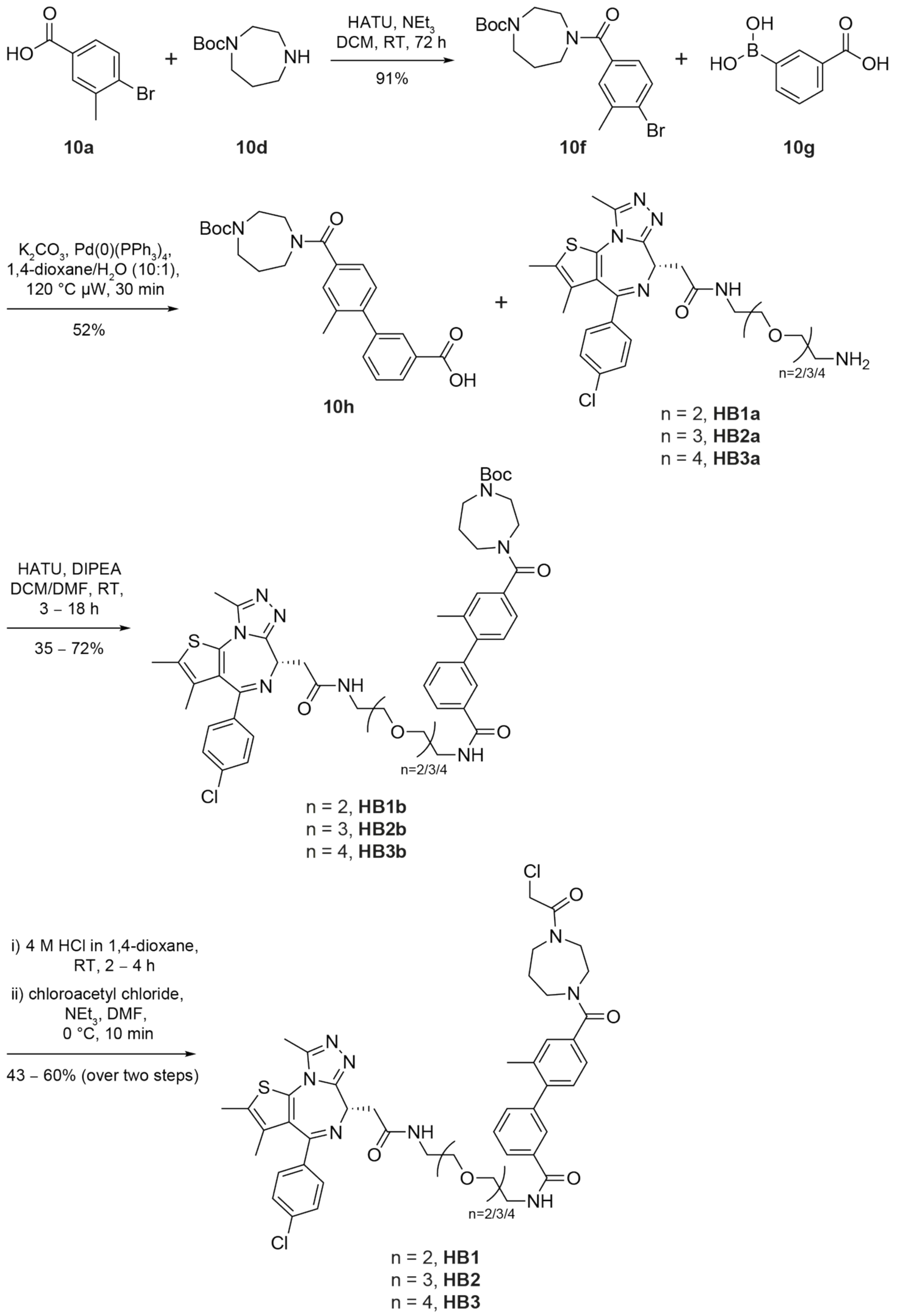

Supplementary Scheme 4 Synthesis of heterobifunctional compounds, HB1 – HB3

S4. Supplementary Data

Supplementary Data 1 Chloroacetamide fragment library

Chloroacetamide fragment library compounds are listed in attached supplementary information .xlsx file (Supplementary Data 1).

Supplementary Data 2 Chemoproteomics processed data for IA-DTB cellular target identification

Mass spectrometry proteomics processed data are attached in .xlsx file (Supplementary Data 2).

S4. Experimental

Recombinant protein expression and purification

Cloning, expression and purification of His_6-UBA1, UBCH5B,[5–7] TRIM2 NHL,[8] and OTU proteins[9] have previously been described. Ubiquitin was purchased commercially (Sigma-Aldrich, U6253). Ubiquitin labelled with ATTO 647N fluorophore (Ub^{ATTO}) was generated as previously described.[7,10] Recombinant full-length BRD4 (short isoform) and BRD4 BD2 (residues 333-462) were kindly received as a gift from Roger George and Karen Chau (The Francis Crick Institute).

TRIM25 PRYSPRY (residues 435-630, purchased as a synthetic gene), TRIM25 PRYSPRY mutants (C498S, C506S), and TRIM25 PRYSPRY-AviTag were cloned into a pACEBac1 vector with a His_6-TEV (Tobacco Etch Virus) tag and expressed in Sf9 insect cells using a baculovirus transposition-based system. Bacmid DNA was transfected into Sf9 cells using Cellfectin II (ThermoFisher Scientific, 10362100), incubated at 28 °C for 72 h, and monitored by GFP expression. Viral titre was subsequently amplified twice before use in a large-scale expression. In general, 2 – 24 L of Sf9 cells (grown in 2 L flasks) in Gibco Sf-900 III serum-free media (ThermoFisher Scientific, 12658027), at 1.2 x 106 cells/mL, were infected with 4 – 6 mL of amplified virus per 500 mL culture, and incubated at 28 °C for 72 h. Cells were then harvested by centrifugation at 2500 rpm in a Beckman J6-M1 rotor for 30 min at 4 °C, the supernatant was discarded and cell pellets were either immediately lysed, or stored at -80°C. The proteins were purified via His_6-capture on HisPur™ Ni-NTA resin (ThermoFisher Scientific, 88222) in buffer 50 mM HEPES pH 7.5, 300 mM NaCl, 0.5 mM TCEP, 20 mM imidazole, washed extensively with both high salt buffer (50 mM HEPES pH 7.5, 1 M NaCl, 0.5 mM TCEP, 20 mM imidazole) and wash buffer (50 mM HEPES pH 7.5, 300 mM NaCl, 0.5 mM TCEP, 20 mM imidazole) before elution in buffer 50 mM HEPES pH 7.5, 300 mM NaCl, 0.5 mM TCEP, 500 mM imidazole, and tag cleavage with TEV protease. The proteins were further purified by size exclusion chromatography into 20 mM HEPES pH 7.5, 150 mM NaCl, 0.5 mM TCEP, and stored at -80 °C. Full-length TRIM25 was cloned, and expressed in Sf9 insect cells, as previously described.[10] The protein was purified via His_{10}-capture on HisPur™ cobalt

resin (ThermoFisher Scientific, 89964), eluted in 100 mM HEPES pH 7, 500 mM NaCl, 300 mM Imidazole, 5% glycerol, 1 mM TCEP, and stored at -80 °C.

TRIM21 PRYSPRY (residues 287-465, purchased as a synthetic gene) was cloned into a pET49 vector with a His_6-HRV-3C (Human Rhinovirus) tag and expressed in BL21 *E.coli* cells (Agilent Technologies; 230132), with induction at 18 °C overnight by addition of isopropyl β-D-1-thiogalactopyranoside (0.5 mM). Cells were harvested by centrifugation at 4000 rpm in a Beckman J6-M1 rotor for 30 min at 4 °C, the supernatant was discarded and cell pellets were either immediately lysed, or stored at -80°C. The protein was purified via His_6-capture on HisPur™ Ni-NTA resin in buffer 50 mM HEPES pH 7.5, 300 mM NaCl, 0.5 mM TCEP, 20 mM imidazole, washed extensively with both high salt buffer (50 mM HEPES pH 7.5, 1 M NaCl, 0.5 mM TCEP, 20 mM imidazole) and wash buffer (50 mM HEPES pH 7.5, 300 mM NaCl, 0.5 mM TCEP, 20 mM imidazole) before elution in buffer 50 mM HEPES pH 7.5, 300 mM NaCl, 0.5 mM TCEP, 500 mM imidazole, and tag cleavage with HRV-3C protease. Cleaved protein was further purified by ion exchange chromatography and size exclusion chromatography into 20 mM HEPES pH 7.5, 150 mM NaCl, 0.5 mM TCEP, and stored at -80 °C.

Intact protein LCMS

For the original chloroacetamide fragment screen, 0.25 µM TRIM25 PRYSPRY or 0.5 µM TRIM21 PRYSPRY were incubated with 50 µM fragments for 24 h at 4 °C, in 25 mM HEPES pH 7.5, 50 mM NaCl buffer. For intact protein liquid chromatography mass spectrometry (LCMS) kinetic characterisation, 0.5 µM TRIM25 PRYSPRY was used. For selectivity testing with recombinant ubiquitin system protein panel, 10 µM protein was incubated with 50 µM compounds for 24 h at 4 °C, in 25 mM HEPES pH 7.5, 50 mM NaCl buffer. Samples were then diluted to 0.5 µM for TRIM25 PRYSPRY, TRIM21 PRYSPRY, OTUD7B OTU, ZRANB1 OTU or 1 µM for TRIM2 NHL, UBCH5B, $UBC13^{K92A}$, UEV1A, OTUD4 OTU, before injection into the LCMS. For TRIM25 PRYSPRY mutants, and for labelling of heterobifunctional compounds, 10 µM protein was incubated with 50 µM compounds for 24 h at 4 °C, in 25 mM HEPES pH 7.5, 50 mM NaCl buffer, and samples were diluted to 1 µM protein before injection into the LCMS.

Intact protein LCMS was performed on an Agilent G6320 ToF Accurate Mass Series mass spectrometer (Model no. G6230B), interfaced with an Agilent 1290 series column oven (Model no. G7116B) and liquid chromatography high speed binary pump (Model no. G7120A). The protein sample was injected using an Agilent 1290 series multisampler with dual needles (Model no. G7167B) with a 5 µL injection volume and maintained at a temperature of 4 °C. Chromatography was carried out on an Agilent Bio-HPLC polymeric reverse-phase (PLRP-S) column (1000 Å, 5 µm × 50 mm × 1.0 mm, PL1312-1502) at 70 °C. The sample was eluted at 0.5 mL/min using a gradient system from Solvent A (water, 0.2% (v/v) formic acid) to Solvent B (acetonitrile, 0.2% (v/v) formic acid), as follows:

Time (min)	Solvent A %	Solvent B %
0.00	80	20
0.60	80	20
0.61	50	50
1.00	0	100
1.40	0	100
1.41	80	20

The eluent was injected directly into an Agilent ToF mass spectrometer (Model no. G6230B) using a dual AJS ESI source and scanning between 600 – 3200 Da with a scan rate of 1.20 s in positive mode. The following MS parameters were used: 4000 V capillary voltage limit, 350 °C desolvation temperature, 10 L/min drying gas flow. Data acquisition was carried out in 2 GHz Extended Dynamic range mode. Spectra were processed using Agilent MassHunter BioConfirm Software 10.0 with the Maximum Entropy method employed. The total ion chromatograms (TIC) were extracted (region containing protein) and the summed scans were deconvoluted (using a maximum entropy algorithm) over an *m/z* range with an expected mass range dependent on the recombinant protein. The following deconvolution conditions were used:

Protein construct	Expected mass range	*m/z* range
TRIM25 PRYSPRY aa435-630	20000 – 26000	350 – 2000

(inc. C498S and C506S mutants)		
TRIM21 PRYSPRY aa287-465	17500 – 25000	350 – 2000
TRIM2 NHL aa465-744	27000 – 33000	300 – 8000
UBCH5B aa1-147	13000 – 20000	300 – 8000
UBC13^{K92A} aa1-152	14000 – 20000	300 – 8000
UEV1A aa1-158	14000 – 21000	300 – 8000
OTUD4 OTU aa1-156	16000 – 20000	350 – 2000
OTUD7B OTU aa129-438	33000 – 38000	350 – 2000
ZRANB1 OTU aa343-692	39000 – 43000	350 – 2000

The deconvoluted mass spectra were exported as csv files and analysed using R Studio (version 1.1.463) to generate .pdf and .csv files of the spectra. The peak height for unlabelled and labelled protein were recorded and used to calculate percentage labelling for each sample using the equation:

$$\%\ \text{labelling} = \frac{intensity\ of\ labelled\ protein}{intensity\ of\ unlabelled\ protein\ +\ intensity\ of\ labelled\ protein} \times 100$$

For cases where the reported *m/z* value for labelled protein did not match the expected value, spectra were visually inspected, and either reprocessed to calculate peak height for the correct *m/z*, or excluded if no peak for correct *m/z* was present.

High-throughput chemistry direct-to-biology (HTC-D2B)

HTC-D2B was performed as previously described.[9,11] Briefly, hit fragments **1** – **3** were used as inputs for high-throughput chemistry (HTC) library design. One library per fragment was designed, using the parent amine SMILES string as an input for a structural similarity search based on the small-world algorithm.[12] Structurally similar amines were searched within GSK solution and solid stocks, using criteria 110<MW<350, primary and/or secondary aromatic amines excluded, and phenols and tricyclic compounds excluded. Anilinic amines, tricyclic motifs and phenol-containing compounds are incompatible with the HTC reaction. For fragment **1**, an HTC library (plate 1) of 83 parent amines was designed by separating the fragment into three distinct areas, left-side heterocyclic ring, central linker, and right-side pendant aromatic

ring, and making variations on each of these areas. For compound **2**, an HTC library (plate 2) of 212 parent amines was designed based on structural similarity of the entire fragment. For compound **3**, an HTC library (plate 3) of 186 parent amines was designed based on structural similarity of the entire fragment.

For each library, the resulting amines (481 total) were plated as 10 mM stock solutions in DMSO (50 µL, 1 eq.) in three separate 384-well plates (one library per plate, Greiner, 781280). To each well containing amine, a solution of *N*-(Chloroacetoxy)succinimide (2 eq.) and *N*,*N*-Diisopropylethylamine (DIPEA) (3 eq.) in DMSO (50 µL) was added, mixed by pipetting and left to incubate for 1 h at RT. A column of DMSO-only controls, and reagent-only controls was also dispensed on the 384-well plate. Following reaction, an aliquot of each reaction mixture (diluted to 2.22 mM) was analysed by LCMS on a Waters® Acquity UPLC instrument equipped with a BEH Acquity UPLC C18 column (50 mm × 2.1 mm, packing diameter: 1.7 µm) at 40 °C with a 0.5 µL injection volume. UV detection was summed from 210 – 350 nm, and mass detection was performed with alternate-scan positive and negative electrospray on a Waters SQD2 instrument, with a scan range of 100–1000 Da and a scan frequency of 5 Hz. The sample was eluted with a flow rate of 1.0 mL/min, using a gradient system from Solvent A (0.1% (v/v) 10 mM ammonium bicarbonate in water adjusted to pH 10 with ammonia solution) to Solvent B (acetonitrile), as follows:

Time (min)	Solvent A %	Solvent B %
0.00	97	3
0.05	97	3
1.50	5	95
1.90	5	95
2.00	97	5

The LCMS data were processed as previously described,[11,13] and chemical conversions for each reaction were recorded as a % purity based on product area under curve (AUC), relative to starting material AUC.

Immediately prior to incubation with TRIM25 PRYSPRY, each reaction mixture was quenched with hydroxylamine (100 µM). 0.5 µM TRIM25 PRYSPRY was incubated with 50 µM and 5 µM HTC-D2B library for 24 h at 4 °C, in 25 mM HEPES pH 7.5, 50 mM NaCl buffer. Intact protein LCMS was performed as described above.

Kinetic characterisation

Eight fragment hits **1** – **8,** and purified optimised compounds **10** – **12** and **11-1** – **11-4** were characterised by kinetic reaction monitoring by intact protein LCMS. 0.5 µM TRIM25 PRYSPRY was incubated with each compound separately, using a 1.5-fold dilution series (final compound concentrations 100 µM (only for fragments **1** – **8**), 67 µM, 44 µM, 29.6 µM, 19.8 µM, 13.2 µM, 8.8 µM, 5.9 µM and 3.9 µM (1% DMSO, and 1% DMSO-only control also used). Assays were performed in technical duplicates for fragments **1** – **8** and compounds **11-1** – **11-4**, and in technical triplicates for compounds **10** – **12**. Protein and compounds were incubated at 4 °C, in 25 mM HEPES pH 7.5, 50 mM NaCl buffer, with each timepoint and replicate in a separate well. Incubation mixtures were sampled at 8 timepoints over 24 h (approximate timepoints 0 h, 2.5 h, 5 h, 7.5 h, 10 h, 12.5 h, 18 h and 24 h). The exact times of each measurement were saved with each reading and used for kinetic calculations. Intact protein masses were recorded, deconvoluted and percentage labelling values extracted in the same way as described above. Percentage labelling values were plotted against time in Prism 10 (GraphPad), and curves fitted separately for each replicate to a one-phase association model with constraints Y0 = 0, and plateau = 100. Observed rate constants (k_{obs}, h^{-1}, generated in Prism 10 as K values) were then plotted against concentration in duplicate (for fragments **1** – **8**, and compounds **11-1** – **11-4**) or in triplicate (for compounds **10** – **12**), and the data was fitted for the mean of two or three replicates to a straight line model with constraint Y-intercept = 0. The gradient of these plots (converted from $\mu M^{-1}h^{-1}$ to $M^{-1}s^{-1}$) gives the pseudo-first order rate parameter, k_{inact}/K_I ($M^{-1}s^{-1}$), for each compound. k_{inact}/K_I values are reported as mean ± SD of the slope based on a linear fit model, n = 2 or 3. For n = 2 data, errors are reported as standard errors of fit, as calculated in Prism 10. For n = 3 data, errors are reported as standard deviation of three individual k_{inact}/K_I values.

Glutathione reactivity assay

Glutathione consumption assay was outsourced to Cyprotex, where the experimental procedure was optimised and performed. 10 mM DMSO stocks of fragments **1** and **2**, and compounds **10** and **11** were diluted 20-fold with acetonitrile, and then further 5-fold diluted with 5 mM glutathione in PBS. The reaction was shaken before incubation at 40 °C. The reaction mixture was analysed via UPLC-UV-MS up to eight times across 24 hours, compared to known reference compounds and samples of each compound in distilled water. UPLC conditions: flow rate 800 µL/min on an Acquity UPLC BEH C18 1.7 µm 2.1 x 50 mm column, with column temperature 37°C, monitored at 210 to 350 nm. Samples were run using a 2 min gradient elution from 97% mobile phase A to 0% mobile phase A, where mobile phase A was 0.1% formic acid in H_2O, and mobile phase B was 0.1% formic acid in 100% MeCN. MS conditions: single quad, ESI+, with scan range of 50 – 1000 Da. For each time point, the UV peak area of the parent peak was extracted at a single wavelength (e.g. 254 nm). A pseudo-first order rate constant (*k*) for each compound was determined from the slope of a linear regression fit for a plot of the logarithm base-10 peak area of the parent compound versus the time differential for the eight time points. $t_{1/2}$ values were calculated as follows: $t_{1/2} = 0.693/k$.

Recombinant protein ubiquitination assays

Ubiquitination assays were all performed using His_6-UBA1 as the E1 enzyme (referred to as 'E1'throughout), and TRIM25 as the E3 ligase. Ubiquitination assays were also spiked with Ub^{ATTO} (ubiquitin labelled with ATTO 647N fluorophore) to visualise ubiquitination activity.[7,10]

For auto-ubiquitination assays, His_{10}-TRIM25 (4 µM) was pre-labelled with either DMSO (1%) or compound **10** or **11** (50 µM, 1% DMSO) in reaction buffer 50 mM HEPES pH 7.5, 150 mM NaCl and 20 mM $MgCl_2$ for 16 h at 4 °C. Ubiquitination assay reaction mixture comprised pre-labelled TRIM25-compound complex (4 µM protein), 0.5 µM UBA1 (E1), 2 µM UBCH5B, 50 µM Ub (Sigma-Aldrich, U6253), 1 µM Ub^{ATTO}, and 3 mM ATP. The reaction buffer contained 50 mM HEPES pH 7.5, 150 mM NaCl and 20 mM $MgCl_2$. All components were mixed together and incubated at 30 °C, with 1000 rpm shaking. Samples of 10 µL were taken at set time intervals (0, 2, 10, 30, 60 min), and mixed 1:1 with NuPAGE LDS sample buffer (2x, Invitrogen, NP0007)

containing 500 mM DTT (ThermoFisher Scientific, R0861). Timepoint at 0 min indicates the sample taken prior to the addition of ATP. Samples were analysed by SDS-PAGE and imaged by Coomassie staining, and using the Odyssey CLx Infrared Imaging System (Li-Cor).

For targeted protein ubiquitination assays with compounds **HB1** – **HB3**, His_{10}-TRIM25 (4 µM) was pre-labelled with either DMSO (1%), control compound **10** (50 µM, 1% DMSO) or compound **HB1**, **HB2** or **HB3** (50 µM, 1% DMSO) in buffer 50 mM HEPES pH 7.5, 150 mM NaCl, 0.5 mM TCEP for 20 h at 4 °C. Protein-compound complexes were captured on His Mag Sepharose Ni magnetic beads (Cytiva, 28967388) in capture buffer 50 mM Tris pH 7.5, 150 mM NaCl, 5 mM $MgCl_2$, 20 mM imidazole, 0.5 mM TCEP, 0.5% IGEPAL for 2 h at 4 °C, with rotation. Beads were washed once with capture buffer to remove excess unreacted compound. Ubiquitination assay reaction mixture comprised components as described above, with the addition of 4 µM BRD4 substrate. For reaction with control compound **HB1a** (JQ1-2PEG), 4 µM **HB1a** was added directly to the ubiquitination assay reaction mixture. Samples were analysed as described above, and by western blotting against BRD4 (Abcam, ab128874, 1:1000), which was detected by anti-rabbit-HRP secondary antibody (Dako, P0399, 1:2000). Blots were developed in Amersham ECL Western Blotting Detection Reagent (Cytiva, RPN2106), imaged on a ChemiDoc MP Imaging System (Bio-Rad), and bands were analysed in ImageLab (Bio-Rad).

Cell treatment and IA-DTB chemoproteomics

Compounds **10** and **11** (50 µM and 10 µM), and DMSO-only controls were dispensed into a 96-deepwell plate. Samples were run in technical triplicate. Live THP-1 cells (2 x 10^6 cells/mL) in Gibco RPMI 1640 medium (ThermoFisher Scientific, 11875085) supplemented with 10% Gibco fetal bovine serum (FBS) (ThermoFisher Scientific, A5670401) were dispensed on top of the compounds or DMSO using an Assist Plus dispenser (Integra) (1 mL cells per well). The plate was incubated for 4 h at 37 °C, 5% CO_2 with 1000 rpm shaking. Cells were pelleted by centrifugation for 5 min at 400 rcf. Media was aspirated and cell pellets were washed with PBS (3 x 2 mL). After final wash, cell pellets were kept in PBS (20 µL), snap frozen in liquid N_2 and stored at -80 °C.

Cysteine profiling was carried out as previously described.[14] Briefly, cells were lysed in 4% SDS, lysates were diluted to 1% SDS and DNA was digested using benzonase. Lysates were diluted to a protein concentration of 2.5 mg/mL. Lysate (20 µL) was incubated with IA-DTB (0.5 mM) for 2 h at RT. Residual IA-DTB was quenched with DTT (5 mM) for 30 min at RT. Remaining free cysteines were alkylated with iodoacetamide (20 mM) for 30 min at RT in the dark. Proteins were digested, labelled with tandem mass tags (TMT) and pooled. IA-DTB-labelled peptides were enriched by incubation with neutravidin beads (25 µL) for 1 h at 4 °C. Beads were washed with 100 mM HEPES (3 x 2 mL), followed by distilled H_2O (3 x 2 mL). IA-DTB-labelled peptides were eluted by incubation with 50% acetonitrile with 0.1% TFA (250 µL) for 20 min with gentle shaking. Samples were lyophilised using a Speedvac and pre-fractionated into three fractions using AssayMAP 5 µL Reversed Phase (RP-S) cartridges on a BRAVO liquid handling station according to manufacturer's instructions.

LCMS/MS analysis and data analysis was performed as previously described.[14,15] Samples were analysed on an Orbitrap Eclipse mass spectrometer (ThermoFisher Scientific). Mascot 2.5 (Matrix Science) was used for peptide and protein identification. Carbamidomethylation of cysteine residues was set as a fixed modification, and methionine oxidation, N-terminal acetylation of proteins, TMT or TMTpro modification of peptide N-termini and lysines, and the addition of IA-DTB on cysteine were set as variable modifications. Analysis was done on peptides filtered as follows: peptide false discovery rate ≤ 1%, signal-to-background of the precursor ion intensity > 4, and signal-to-interference > 0.5. Fold changes were corrected for isotope purity and adjusted for interference caused by co-eluting nearly isobaric peptides as estimated by the signal-to-interference measure.[16] Peptides were further filtered for presence of the IA-DTB mass tag on cysteine. A two-sided t-test was used to determine significance. Peptides with a p-value ≤ 0.01 and a fold change ≤ 0.25 were considered significantly affected. Processed data are attached in .xlsx file (Supplementary Data 2).

X-ray crystallography

TRIM25 PRYSPRY (10 μM) was pre-labelled with compound **10** (50 μM) in buffer 25 mM HEPES, 50 mM NaCl for 20 h at 4 °C, before purification by gel filtration into buffer 20 mM Bis-Tris pH 7.5, 150 mM NaCl, 0.5 mM TCEP. Commercially available sitting drop crystallization screens were dispensed at 20 °C using an automated Mosquito machine (TTP Labtech). Crystals grew from a pre-labelled protein-ligand complex solution (at 13.8 mg/mL) in 0.1 M CHES pH 9.5, 20% w/v PEG 8000, with drops containing 100 nL protein-ligand complex and 100 nL reservoir solution. For X-ray data acquisition, crystals were cryoprotected with perfluoropolyether cryo oil (Hampton Research, HR2-814). Diffraction data were collected on beamline I04 (λ = 0.9537 Å) at Diamond Light Source (Oxford, UK), processed using DIALS[17] and merged and scaled using AIMLESS.[18] The structure of the complex was solved by molecular replacement using the available TRIM25 PRYSPRY structure (6FLM)[10] as a template in Phenix Phaser.[19] Models were iteratively improved by manual building in Coot[20] and refined using Refmac[21] and Phenix.[19] Coordinates and structure factors are deposited in the Protein Data Bank under accession code 9I0T. Further details on data collection and refinement statistics are summarised in Supplementary Table 1.

Ligand-based ^{1}H NMR

Recombinant TRIM25 PRYSPRY (100 μM) in 25 mM HEPES pH 7.5, 50 mM NaCl, 5% D_2O and 1% DMSO-d6 was mixed with compound **10** (100 μM). ^{1}H NMR spectra were recorded at 298 K on a Bruker Avance 700 MHz spectrometer, and data were acquired with Topspin (Bruker) at timepoint 0 (immediately after mixing protein with compound), and after 4 h incubation.

Recombinant protein ternary complex pull-down assays

His_{10}-TRIM25 or His_6-TRIM25 PRYSPRY (4 μM) was pre-labelled with either DMSO (1%) or heterobifunctional compounds (**HB1**, **HB2** or **HB3**, 50 μM, 1% DMSO) and captured on His Mag Sepharose Ni magnetic beads, and washed once to remove excess unreacted compound, as described above.

Substrate (BRD4 or BRD4 BD2) (4 μM, assuming 1:1 with on-bead captured TRIM25-compound complex) was added to the on-bead captured TRIM25-compound complex,

and incubated in capture buffer 50 mM Tris pH 7.5, 150 mM NaCl, 5 mM $MgCl_2$, 20 mM imidazole, 0.5 mM TCEP, 0.5% IGEPAL for 2 h at 4 °C, with rotation. Beads were washed three times with 500 µL capture buffer, and mixed with 12 µL NuPAGE LDS sample buffer (1x, Invitrogen, NP0007). Samples were analysed by SDS-PAGE and imaged by Coomassie staining.

Ternary complex SPR

TRIM25 PRYSPRY-AviTag (10 µM) was pre-labelled with either DMSO (2%) or heterobifunctional compound **HB1**, **HB2** or **HB3** (100 µM, 2% DMSO) in buffer 50 mM HEPES, 150 mM NaCl, 0.5 mM TCEP, for 18 h at 4 °C.

SPR experiments were carried out on a Biacore S200 instrument (Cytiva), using a Series S streptavidin sensor chip (Cytiva, 29699621). Protein dilutions and SPR experiments were performed in filtered buffer 20 mM HEPES pH 7.5, 150 mM NaCl, 0.5 mM TCEP, and 0.05% TWEEN 20. Each pre-labelled TRIM25 PRYSPRY-AviTag-**HB** compound reaction mixture was loaded onto sensor chip at 350 RU. BRD4 BD2 dilution series (250 nM, 125 nM, 62.5 nM, 31.3 nM, 15.6 nM, 7.8 nM, 3.9 nM) was flowed across the sensor chip, in triplicate, at 30 µL/min, contact time 40 s, dissociation time 600 s at 25 °C. Note, BRD4 BD2 sample compartment was kept at 20 °C. Biacore S200 Control software and Biacore S200 Evaluation software (Cytiva) were used for SPR set-up and analysis. Steady state analysis was performed using the 1:1 binding model in the Biacore S200 Evaluation software. Equilibrium binding constants (K_D) were estimated by plotting the instrument response against analyte concentration in Prism 10 (GraphPad), where curves were fitted separately for each replicate to a one site – specific binding model. Data are presented as individual replicates, n = 3, and K_D values are reported as mean ± SD, n = 3.

Small-angle X-ray scattering (SAXS)

SEC-SAXS data were collected at the B21 beamline at Diamond Light Source (Oxford, UK). TRIM25 PRYSPRY, BRD4 BD2 and TRIM25 PRYSPRY-**HB2**-BRD4 BD2 complex samples were prepared in 20 mM HEPES pH 7.5, 150 mM NaCl, 0.5 mM TCEP. Samples were injected onto a Superdex 75 3.2 x 300 column equilibrated with buffer 20 mM HEPES pH 7.5, 150 mM NaCl, 0.5 mM TCEP and eluted at a flow rate

of 0.075 mL/min at 15 °C with 3 s exposures. Frames were collected continuously during the fractionation of the proteins. Frames collected before the void volume were averaged and subtracted from the signal of the elution profile to account for background scattering. Data reduction, subtraction and averaging within the SEC peak with constant radius of gyration were performed using the software ScÅtterIV (www.bioisis.net). The scattering curves were analyzed using the package ATSAS[22] and reported as function of the angular momentum transfer $q = 4\pi/\lambda \sin\theta$, where 2θ is the scattering angle and λ the wavelength of the incident beam. Values of the cross-sectional radius of gyration (R_c) were calculated in ScÅtterIV.[23] Low-resolution ab initio models for the ternary complex was generated by the program DAMMIF. The SAXS-derived dummy model was rendered by Pymol. The SAXS data and the dummy-atom models statistics are reported in Supplementary Table 2.

Synthetic Chemistry

General techniques

Solvents used in synthetic reactions were anhydrous, and all reagents purchased from commercial suppliers were used without further purification. Compounds **HB1a**, **HB2a** and **HB3a** were synthesised as previously described.[24] Room temperature reactions were carried out at ~20 °C. Microwave-assisted reactions were performed using an Initiator+ microwave reactor (Biotage). Where reactions were performed under an inert atmosphere (N_2), conventional glassware was purged with N_2 before use, where purging refers to a vacuum/nitrogen-refilling procedure. Deionised water (Milli-Q or dH_2O) was used where stated in reactions, extractions, and to make all buffers. Purification by flash chromatography was carried out on an Isolera One Flash Chromatography System (Biotage), using Sfär Silica Duo 60 μm cartridges (normal-phase, Biotage) or Sfär C18 Duo 30 μm cartridges (reverse-phase, Biotage). Solvents were removed by rotary evaporation (BÜCHI) or by lyophilization (freeze-drying) using a BenchTop Pro with Omnitronics (SP Scientific).

Characterisation

^1H and ^{13}C NMR spectra were obtained on a Bruker Advance 400 spectrometer at 400 and 101 MHz, respectively. Chemical shifts (δ) are reported in parts per million (ppm),

referenced to residual solvent signals: DMSO-d6 = 2.50 (^{1}H) and 39.52 (^{13}C) ppm, $CDCl_3$ = 7.26 (^{1}H) and 77.16 (^{13}C) ppm, and MeOD-d4 = 3.31 (^{1}H) and 49.00 (^{13}C) ppm. Coupling constants (J) are reported in Hz to 1 decimal place, and multiplicities are denoted as follows: s = singlet, d = doublet, t = triplet, q = quartet, quint = quintet, m = multiplet, and br s = broad singlet.

LCMS spectra were obtained using a UPLC-MS system (Waters) equipped with an Acquity UPLC BEH C18 column, 130 Å, 1.7 µm, 2.1 mm x 50 mm (Waters), monitored over 210 – 400 nm. Samples were run using a gradient elution from 97 – 5% over 4 min, 0.25 mL/min flow, solvent A: 0.1% formic acid in H_2O, solvent B: 0.1% formic acid in MeCN, with a 1 µL injection. LCMS retention times (R_t) are reported in minutes.

High-resolution mass spectrometry (HRMS) spectra were obtained using a Xevo G2-XS QTOF instrument (Waters) equipped with an Acquity Premier LC, BEH C18 column with VanGuard FIT, 130 Å, 1.7 µm, 2.1 mm x 50 mm (Waters), monitored over 210 – 400 nm. Samples were run using a gradient elution from 97 – 5% over 4 min, 0.25 mL/min flow, solvent A: 0.1% formic acid in H_2O, solvent B: 0.1% formic acid in MeCN, with a 1 µL injection. m/z values are reported in Daltons.

Synthesis of compound 10 (see Scheme 1)

2-methyl-[1,1'-biphenyl]-4-carboxylic acid (10c)

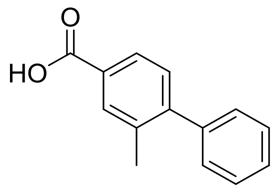

10c

To a mixture of 4-bromo-3-methylbenzoic acid **10a** (201 mg, 0.934 mmol), phenylboronic acid **10b** (103 mg, 0.845 mmol), K_2CO_3 (228 mg, 1.65 mmol) and Pd(0)$(PPh_3)_4$ (3 mol%, 27.3 mg, 0.0236 mmol), under a nitrogen atmosphere, was added 1,4-dioxane/H_2O (10:1, 2.2 mL). The reaction mixture was heated to 120 °C with stirring in a microwave reactor for 30 min. The reaction mixture was filtered through celite and the pad was washed with DCM (5 mL). The filtrate was concentrated

under reduced pressure, and the residue was taken up in water (20 mL). The aqueous was acidified to pH 4 with an aqueous solution of 1 M HCl, and extracted with EtOAc (2 x 20 mL). The combined organics were dried over Na_2SO_4 and concentrated under reduced pressure. The residue was purified by flash column chromatography (normal phase, eluted with 0 – 50% EtOAc in cyclohexane) to yield 2-methyl-[1,1'-biphenyl]-4-carboxylic acid **10c**, as a white solid (136 mg, 0.642 mmol, 76%). **^{1}H NMR** (400 MHz, $CDCl_3$) δ 8.03 (dt, *J* = 1.9, 0.6 Hz, 1H), 7.98 (ddd, *J* = 8.0, 1.9, 0.7 Hz, 1H), 7.48 – 7.42 (m, 2H), 7.41 – 7.37 (m, 1H), 7.35 – 7.32 (m, 3H), 2.34 (s, 3H). **^{13}C NMR** (101 MHz, $CDCl_3$) δ 170.9, 147.6, 141.0, 136.0, 132.2, 130.2, 129.0, 128.4, 128.0, 127.8, 127.6, 20.6. **LCMS** ($C_{14}H_{12}O_2$) $[M+H]^+$ required 213.1, $[M+H]^+$ found 213.1. (Formic) R_t = 2.78 min. Analytical data consistent with that reported in literature.[25]

tert-butyl 4-(2-methyl-[1,1'-biphenyl]-4-carbonyl)-1,4-diazepane-1-carboxylate (10e)

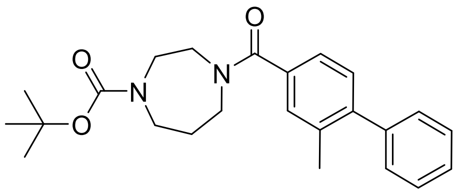

10e

To a solution of 2-methyl-[1,1'-biphenyl]-4-carboxylic acid **10c** (51.5 mg, 0.243 mmol) in DCM (2 mL) was added NEt_3 (200 µL, 1.435 mmol), and the solution was stirred for 5 min. HATU (226 mg, 0.594 mmol) was added and the reaction mixture was stirred for 10 min. *tert*-butyl 1,4-diazepane-1-carboxylate **10d** (100 µL, 0.508 mmol) was added to the reaction mixture, and stirred at room temperature for 18 h. The reaction mixture was separated with DCM (3 x 10 mL) and a saturated aqueous solution of NH_4Cl (10 mL). The combined organics were dried through a phase separator, and concentrated under reduced pressure. The residue was purified by flash column chromatography (normal phase, eluted with 0 – 80% EtOAc in cyclohexane) to yield *tert*-butyl 4-(2-methyl-[1,1'-biphenyl]-4-carbonyl)-1,4-diazepane-1-carboxylate **10e**, as a clear gum (85.8 mg, 0.218 mmol, 90%). **^{1}H NMR** (400 MHz, $CDCl_3$) δ 7.42 (tt, *J* = 6.7, 1.1 Hz, 2H), 7.37 – 7.34 (m, 1H), 7.31 – 7.26 (m, 3H), 7.25 – 7.20 (m, 2H), 3.82 (br s, 1H), 3.72 (t, *J* = 5.9 Hz, 1H), 3.64 (br s, 1H), 3.57 – 3.53 (m, 2H), 3.46 (br t, *J* =

5.9 Hz, 3H), 2.27 (s, 3H), 2.03 – 1.99 (m, 1H), 1.80 – 1.70 (m, 1H), 1.48 (s, 9H). ^{13}**C NMR (**101 MHz, $CDCl_3$) δ 172.0, 155.3, 143.3, 141.3, 136.1, 135.6, 130.0, 128.7, 128.3, 127.3, 124.0, 80.0, 50.3, 47.5, 45.4, 28.6, 27.1, 20.6. **LCMS** ($C_{24}H_{30}N_2O_3$) $[M+H]^+$ required 395.2, $[M+H]^+$ not found, poor ionisation. (Formic) R_t = 3.12 min.

2-chloro-1-(4-(2-methyl-[1,1'-biphenyl]-4-carbonyl)-1,4-diazepan-1-yl)ethan-1-one (10)

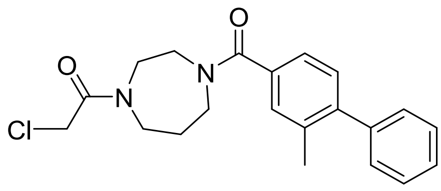

10

To a solution of *tert*-butyl 4-(2-methyl-[1,1'-biphenyl]-4-carbonyl)-1,4-diazepane-1-carboxylate **10e** (37.1 mg, 0.094 mmol) in DCM (1 mL), under a nitrogen atmosphere, was added HCl in MeOH (3 M, 0.6 mL, 1.8 mmol) and stirred for 3 h. The reaction mixture was concentrated under reduced pressure, co-evaporating with DCM (3 x 10 mL). The residue was resuspended in DCM (1 mL), under a nitrogen atmosphere at 0 °C, NEt_3 (0.1 mL, 0.725 mmol) was added, and the reaction mixture was stirred for 5 min. Chloroacetyl chloride (20 µL, 0.25 mmol) was added dropwise at 0 °C, and the reaction mixture was stirred for 10 min. The reaction mixture was diluted with DCM (10 mL) and quenched with H_2O (10 mL). The layers were separated and the aqueous layer was extracted with DCM (2 x 10 mL). The combined organics were washed with a saturated aqueous solution of NH_4Cl (2 x 30 mL). The organic layer was dried through a phase separator, and concentrated under reduced pressure. The residue was purified by flash column chromatography (normal phase, eluted with 0 – 5% MeOH in EtOAc) to yield 2-chloro-1-(4-(2-methyl-[1,1'-biphenyl]-4-carbonyl)-1,4-diazepan-1-yl)ethan-1-one (**10**), as a clear gum (18.5 mg, 0.050 mmol, 53%). ^{1}**H NMR** (400 MHz, $CDCl_3$) δ 7.45 – 7.40 (m, 2H), 7.38 – 7.33 (m, 1H), 7.31 (t, *J* = 1.9 Hz, 1H), 7.29 (dd, *J* = 2.2, 1.2 Hz, 1H), 7.24 – 7.18 (m, 3H), 4.18 (s, 1H), 4.11 (s, 1H), 3.93 (t, *J* = 5.7 Hz, 1H), 3.81 (t, *J* = 6.1 Hz, 2H), 3.69 – 3.64 (m, 3H), 3.53 (t, *J* = 6.2 Hz, 2H), 2.28 (s, 3H), 2.13 (br s, 1H), 1.79 – 1.73 (m, 1H). ^{13}**C NMR** (101 MHz, $CDCl_3$) δ 166.9, 150.6, 143.6, 136.2, 130.1, 129.2, 128.4, 127.4, 123.8, 50.5, 47.2, 44.3, 27.7, 20.6.

LCMS ($C_{21}H_{23}ClN_2O_2$) $[M+H]^+$ required 371.2, $[M+H]^+$ found 371.3. (Formic) R_t = 2.63 min. **HRMS** ($C_{21}H_{23}ClN_2O_2$) $[M+H]^+$ required 371.1526, $[M+H]^+$ found 371.1521.

Synthesis of compound 11 (see Scheme 2)

***tert*-butyl 3-(2-methylindoline-1-carbonyl)piperidine-1-carboxylate (11c)**

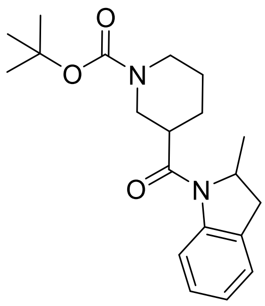

11c

To a solution of 1-(*tert*-butoxycarbonyl)piperidine-3-carboxylic acid **11a** (517 mg, 2.26 mmol) in DMF (6 mL) was added DIPEA (500 µL, 2.87 mmol), and the solution was stirred for 5 min. HATU (1132 mg, 2.98 mmol) was added and the reaction mixture was stirred for 10 min. 2-methylindoline **11b** (350 µL, 2.68 mmol) was added to the reaction mixture, and stirred at room temperature for 24 h. The reaction mixture was separated with DCM (3 x 10 mL) and a saturated aqueous solution of NH_4Cl (10 mL). The combined organics were washed with an aqueous solution of 5% LiCl (3 x 10 mL), dried through a phase separator, and concentrated under reduced pressure. The residue was purified by flash column chromatography (normal phase, eluted with 20 – 100% EtOAc in cyclohexane) to yield *tert*-butyl 3-(2-methylindoline-1-carbonyl)piperidine-1-carboxylate **11c**, as a brown gum (693 mg, 2.01 mmol, 89%). **^{1}H NMR** (400 MHz, $CDCl_3$) δ 8.19 (d, *J* = 8.0 Hz, 1H), 7.21 (t, *J* = 8.0 Hz, 2H), 7.04 (td, *J* = 7.4, 1.1 Hz, 1H), 4.53 (quint, *J* = 7.5 Hz, 1H), 4.21 (br s, 2H), 3.42 (dd, *J* = 15.7, 8.8 Hz, 1H), 3.09 (br s, 1H), 2.78 – 2.57 (m, 2H), 2.03 – 1.95 (m, 1H), 1.85 – 1.69 (m, 2H), 1.60 (s, 2H), 1.46 (s, 9H), 1.38 (d, *J* = 6.2 Hz, 3H). **^{13}C NMR** (101 MHz, $CDCl_3$) δ 171.3, 154.8, 130.6, 127.7, 125.1, 124.3, 118.3, 79.9, 55.5, 36.5, 28.6, 27.7, 27.1, 22.7. **LCMS** ($C_{20}H_{28}N_2O_3$) $[M+H-Boc]^+$ required 245.2, $[M+H-Boc]^+$ found 245.2. (Formic) R_t = 3.09 min.

2-chloro-1-(3-(2-methylindoline-1-carbonyl)piperidin-1-yl)ethan-1-one (11)

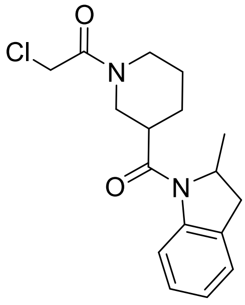

11

To a solution of *tert*-butyl 3-(2-methylindoline-1-carbonyl)piperidine-1-carboxylate **11c** (112 mg, 0.325 mmol) in DCM (2 mL), under a nitrogen atmosphere, was added HCl in MeOH (3 M, 2 mL, 6.0 mmol) and stirred for 3 h. The reaction mixture was concentrated under reduced pressure, co-evaporating with DCM (3 x 10 mL). The residue was resuspended in DCM (2 mL), under a nitrogen atmosphere at 0 °C, NEt_3 (170 µL, 1.22 mmol) was added, and the reaction mixture was stirred for 5 min. Chloroacetyl chloride (30 µL, 0.377 mmol) was added dropwise at 0 °C, and the reaction mixture was stirred for 10 min. The reaction mixture was diluted with DCM (10 mL) and quenched with H_2O (10 mL). The layers were separated and the aqueous layer was extracted with DCM (2 x 10 mL). The combined organics were washed with a saturated aqueous solution of NH_4Cl (2 x 30 mL). The organic layer was dried through a phase separator, and concentrated under reduced pressure. The residue was purified by flash column chromatography (normal phase, eluted with 0 – 10% MeOH in DCM) to yield 2-chloro-1-(3-(2-methylindoline-1-carbonyl)piperidin-1-yl)ethan-1-one (**11**) as a brown gum (56.2 mg, 0.176 mmol, 54%). **^{1}H NMR** (400 MHz, $CDCl_3$) δ 8.17 (br d, *J* = 8.0 Hz, 1H), 7.21 (t, *J* = 7.4 Hz, 2H), 7.06 (q, *J* = 7.4 Hz, 1H), 4.78 – 4.41 (m, 2H), 4.20 – 3.88 (m, 3H), 3.50 – 3.38 (m, 1H), 3.19 (td, *J* = 13.2, 2.9 Hz, 1H), 2.82 – 2.66 (m, 2H), 2.14 – 1.81 (m, 3H), 1.70 – 1.60 (m, 1H), 1.37 (dd, *J* = 16.5, 6.4 Hz, 1H), 1.32 – 1.22 (m, 3H). **^{13}C NMR** (101 MHz, $CDCl_3$) δ 170.7, 165.8, 141.2, 131.0, 127.8, 125.2, 124.6, 118.4, 55.8, 47.1, 46.0, 42.9, 41.9, 41.0, 36.5, 27.5, 25.4, 22.7. **LCMS** ($C_{17}H_{21}ClN_2O_2$) $[M+H]^+$ required 321.1, $[M+H]^+$ found 321.2. (Formic) R_t = 2.51 min. **HRMS** ($C_{17}H_{21}ClN_2O_2$) $[M+H]^+$ required 321.1370, $[M+H]^+$ found 321.1371.

2-chloro-1-((*S* or *R*)-3-((*R* or *S*)-2-methylindoline-1-carbonyl)piperidin-1-yl)ethan-1-one (11-1, 11-2, 11-3 and 11-4)

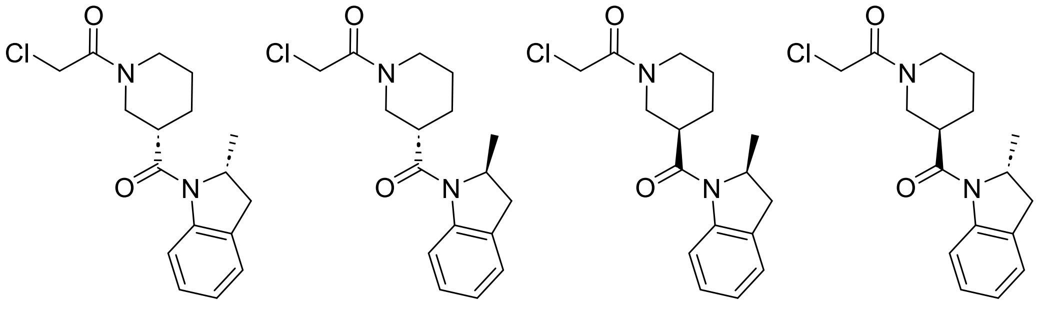

11-1, **11-2**, **11-3** and **11-4**
–
Definitive stereochemistry cannot be assigned

Racemic compound **11** (182 mg, 0.569 mmol) was separated into the corresponding single enantiomers (**11-1**, **11-2**, **11-3** and **11-4**) by preparative HPLC using a Chiralpak IC chiral column (5 µm, 250 x 30 mm), with isocratic elution in EtOAc/heptane (80/20, v/v) at a flow rate of 30 mL/min, and a detection wavelength of 250 nm. The combined fractions for each isomer were concentrated under reduced pressure, and analysed using the same method as for prep HPLC, but using a smaller Chiralpak IC chiral column (5 µm, 250 x 4.6 mm), with a flow rate of 1 mL/min, to obtain diastereomeric purity values by UV, **11-1** = 100% purity, **11-2** = 98.1% purity, **11-3** = 97% purity, and **11-4** = 100% purity. Isomers were redissolved in DCM/heptane (1:1, 5 mL) and blown down under nitrogen to obtain a solid, **11-1** (27 mg, 0.0843 mmol, 15%), **11-2** (32 mg, 0.100 mmol, 18%), **11-3** (25 mg, 0.0781 mmol, 14%), and **11-4** (23 mg, 0.0718 mmol, 13%).

<u>11-1</u>: ^{1}H NMR (400 MHz, $CDCl_3$) δ 8.17 (d, *J* = 8.3 Hz, 1H), 7.24 – 7.18 (m, 2H), 7.06 (q, *J* = 6.7 Hz, 1H), 4.76 – 4.58 (m, 2H), 4.20 – 4.04 (m, 2H), 4.03 – 3.85 (m, 1H), 3.52 – 3.35 (m, 1H), 3.19 (td, *J* = 13.2, 2.9 Hz, 1H), 2.84 – 2.62 (m, 2H), 2.16 – 1.99 (m, 3H), 1.97 – 1.82 (m, 1H), 1.72 – 1.64 (m, 1H), 1.25 – 1.19 (m, 3H). **LCMS** ($C_{17}H_{21}ClN_2O_2$) $[M+H]^+$ required 321.1, $[M+H]^+$ found 321.2. (Formic) R_t = 2.52 min. **HRMS** ($C_{17}H_{21}ClN_2O_2$) $[M+H]^+$ required 321.1370, $[M+H]^+$ found 321.1365.

<u>11-2</u>: ^{1}H NMR (400 MHz, $CDCl_3$) δ 8.17 (d, *J* = 8.3 Hz, 1H), 7.24 – 7.18 (m, 2H), 7.06 (q, *J* = 6.7 Hz, 1H), 4.76 – 4.57 (m, 2H), 4.20 – 4.05 (m, 2H), 4.04 – 3.84 (m, 1H), 3.52

– 3.34 (m, 1H), 3.19 (td, *J* = 13.2, 2.9 Hz, 1H), 2.84 – 2.62 (m, 2H), 2.16 – 1.98 (m, 3H), 1.97 – 1.82 (m, 1H), 1.72 – 1.63 (m, 1H), 1.25 – 1.18 (m, 3H). **LCMS** ($C_{17}H_{21}ClN_2O_2$) $[M+H]^+$ required 321.1, $[M+H]^+$ found 321.2. (Formic) R_t = 2.52 min. **HRMS** ($C_{17}H_{21}ClN_2O_2$) $[M+H]^+$ required 321.1370, $[M+H]^+$ found 321.1371.

11-3: ^{1}H NMR (400 MHz, $CDCl_3$) δ 8.17 (t, *J* = 6.8 Hz, 1H), 7.21 (t, *J* = 7.6 Hz, 2H), 7.06 (q, *J* = 7.7 Hz, 1H), 4.73 (d, *J* = 13.4 Hz, 1H), 4.60 (d, *J* = 13.4 Hz, 1H), 4.53 – 4.42 (m, 1H), 4.21 – 3.86 (m, 2H), 3.59 (br t, *J* = 13.6 Hz, 1H), 3.45 – 3 36 (m, 1H), 3.20 (br t, *J* = 12.7 Hz, 1H), 3.01 (br t, *J* = 12.1 Hz, 1H), 2.75 – 2.63 (m, 2H), 2.14 – 2.01 (m, 1H), 1.98 – 1.76 (m, 2H), 1.37 (dd, *J* = 16.3, 6.3 Hz, 3H). **LCMS** ($C_{17}H_{21}ClN_2O_2$) $[M+H]^+$ required 321.1, $[M+H]^+$ found 321.2. (Formic) R_t = 2.49 min. **HRMS** ($C_{17}H_{21}ClN_2O_2$) $[M+H]^+$ required 321.1370, $[M+H]^+$ found 321.1380.

11-4: ^{1}H NMR (400 MHz, $CDCl_3$) δ 8.17 (t, *J* = 6.8 Hz, 1H), 7.21 (t, *J* = 7.6 Hz, 2H), 7.06 (q, *J* = 7.7 Hz, 1H), 4.73 (d, *J* = 13.2 Hz, 1H), 4.60 (d, *J* = 13.2 Hz, 1H), 4.54 – 4.42 (m, 1H), 4.22 – 3.85 (m, 2H), 3.59 (br t, *J* = 13.6 Hz, 1H), 3.46 – 3.36 (m, 1H), 3.20 (br t, *J* = 12.7 Hz, 1H), 3.01 (t, *J* = 12.1 Hz, 1H), 2.74 – 2.63 (m, 2H), 2.14 – 2.01 (m, 1H), 1.99 – 1.79 (m, 2H), 1.37 (dd, *J* = 16.1, 6.4 Hz, 3H). **LCMS** ($C_{17}H_{21}ClN_2O_2$) $[M+H]^+$ required 321.1, $[M+H]^+$ found 321.2. (Formic) R_t = 2.49 min. **HRMS** ($C_{17}H_{21}ClN_2O_2$) $[M+H]^+$ required 321.1370, $[M+H]^+$ found 321.1372.

Synthesis of compound 12 (see Scheme 3)

tert-butyl 3-((3-cyanopyridin-4-yl)carbamoyl)piperidine-1-carboxylate (12c)

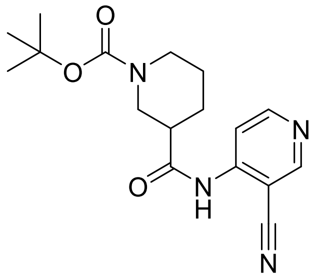

12c

To a mixture of *tert*-butyl 3-carbamoylpiperidine-1-carboxylate **12a** (225 mg, 0.986 mmol), 4-chloronicotinonitrile **12b** (102 mg, 0.736 mmol), Cs_2CO_3 (482 mg, 1.48 mmol) and Pd(II)(dppf)Cl_2.DCM (4.5 mol%, 27.1 mg, 0.0332 mmol), under a nitrogen atmosphere, was added 1,4-dioxane (2.5 mL). The reaction mixture was heated to 120 °C with stirring in a microwave reactor for 30 min. The reaction mixture was filtered through celite and the pad was washed with DCM (5 mL). The filtrate was concentrated under reduced pressure, and the residue was separated with EtOAc (3 x 20 mL) and a saturated aqueous solution of NH_4Cl (20 mL). The combined organics were dried over Na_2SO_4 and concentrated under reduced pressure. The residue was purified by flash column chromatography (normal phase, eluted with 0 – 80% EtOAc in cyclohexane) to yield *tert*-butyl 3-((3-cyanopyridin-4-yl)carbamoyl)piperidine-1-carboxylate **12c**, as a clear gum (167 mg, 0.505 mmol, 69%). **^{1}H NMR** (400 MHz, $CDCl_3$) δ 8.76 (d, *J* = 0.6 Hz, 1H), 8.67 (d, *J* = 5.9 Hz, 1H), 8.42 (d, *J* = 5.9 Hz, 1H), 8.01 (br s, 1H), 4.20 – 4.13 (m, 1H), 3.94 (br s, 1H), 3.15 (dd, *J* = 13.5, 9.8 Hz, 1H), 2.98 – 2.86 (m, 1H), 2.59 – 2.51 (m, 1 H), 2.10 – 2.04 (m, 1H), 1.89 – 1.75 (m, 2H), 1.60 – 1.51 (m, 1H), 1.47 (s, 9H). **^{13}C NMR** (101 MHz, $CDCl_3$) δ 172.5, 164.2, 154.5, 153.2, 146.7, 114.5, 114.0, 80.4, 45.9, 44.4, 28.5, 28.0, 24.1. **LCMS** ($C_{17}H_{22}N_4O_3$) $[M+H]^+$ required 331.2, $[M+H]^+$ found 331.2. (Formic) R_t = 2.44 min.

1-(2-chloroacetyl)-*N*-(3-cyanopyridin-4-yl)piperidine-3-carboxamide (12)

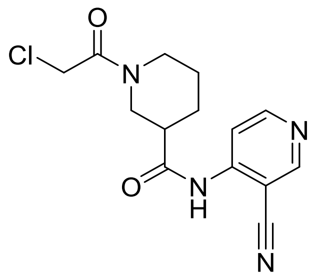

12

To a solution of *tert*-butyl 3-((3-cyanopyridin-4-yl)carbamoyl)piperidine-1-carboxylate **12c** (74.2 mg, 0.225 mmol) in 1,4-dioxane (1 mL) was added HCl in 1,4-dioxane (4 M, 1.2 mL, 4.5 mmol), and the reaction mixture was stirred for 3 h. The reaction mixture was concentrated under reduced pressure, co-evaporating with DCM (3 x 10 mL). To the residue was added a solution of N-(chloroacetoxy)succinimide (85.9 mg, 0.448 mmol) in DMSO (1 mL), followed by DIPEA (200 µL, 1.15 mmol), and the reaction mixture was stirred for 1 h. The reaction mixture was purified by flash column chromatography (reverse-phase, eluted with 0 – 50% MeCN in water). Freeze-drying afforded 1-(2-chloroacetyl)-*N*-(3-cyanopyridin-4-yl)piperidine-3-carboxamide (**12**) as an off-white solid (17.5 mg, 0.057 mmol, 25%). **^{1}H NMR** (400 MHz, DMSO) δ 10.57 (br s, 1H), 8.91 (d, *J* = 2.9 Hz, 1H), 8.71 (d, *J* = 5.8 Hz, 1H), 7.83 (dd, *J* = 16.4, 5.8 Hz, 1H), 4.52 – 4.32 (m, 2H), 4.14 (d, 1H), 3.93 – 3.76 (m, 1H), 3.14 – 3.05 (m, 1H), 2.88 – 2.75 (m, 1H), 2.71 – 2.59 (m, 1H), 2.09 – 1.98 (m, 1H), 1.83 – 1.63 (m, 2H), 1.57 – 1.33 (m, 1H). **^{13}C NMR** (101 MHz, DMSO) δ 172.9, 163.1, 154.1, 153.5, 147.1, 135.0, 116.8, 115.1, 48.6, 42.3, 41.8, 33.0, 27.5, 23.7. **LCMS** ($C_{14}H_{15}ClN_4O_2$) $[M+H]^+$ required 307.1, $[M+H]^+$ found 307.1. (Formic) R_t = 1.82 min. **HRMS** ($C_{14}H_{15}ClN_4O_2$) $[M+H]^+$ required 307.0962, $[M+H]^+$ found 307.0953.

Synthesis of heterobifunctional compounds HB1, HB2 and HB3 (see Scheme 4)

tert-butyl 4-(4-bromo-3-methylbenzoyl)-1,4-diazepane-1-carboxylate (10f)

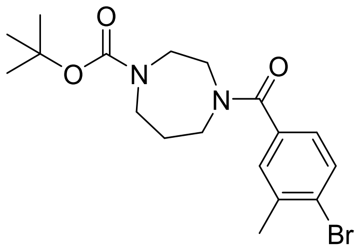

10f

To a solution of 4-bromo-3-methylbenzoic acid **10a** (500 mg, 2.33 mmol) in DCM (10 mL) was added NEt_3 (680 µL, 4.88 mmol), and the solution was stirred for 5 min. HATU (1138 mg, 2.99 mmol) was added and the reaction mixture was stirred for 10 min. *tert*-butyl 1,4-diazepane-1-carboxylate **10d** (550 µL, 2.79 mmol) was added to the reaction mixture, and stirred at room temperature for 72 h. The reaction mixture was separated with DCM (3 x 10 mL) and a saturated aqueous solution of NH_4Cl (10 mL). The combined organics were dried through a phase separator, and concentrated under reduced pressure. The residue was purified by flash chromatography (normal phase, eluted with 20 – 100% EtOAc in cyclohexane) to yield *tert*-butyl 4-(4-bromo-3-methylbenzoyl)-1,4-diazepane-1-carboxylate **10f**, as a clear gum (840 mg, 2.11 mmol, 91%). **^{1}H NMR** (400 MHz, $CDCl_3$) δ 7.54 (d, *J* = 8.1 Hz, 1H), 7.24 (s, 1H), 7.03 (d, *J* = 8.1 Hz, 1H), 3.77 (br t, *J* = 5.6 Hz, 1H), 3.64 (br dt, *J* = 26.9, 5.7 Hz, 2H), 3.50 – 3.36 (m, 5H), 2.40 (s, 3H), 1.99 – 1.92 (m, 1H), 1.71 – 1.62 (m, 1H), 1.47 (s, 9H). **^{13}C NMR** (100 MHz, $CDCl_3$) δ 171.0, 155.2, 138.7, 132.6, 129.2, 126.3, 125.5, 125.2, 80.1, 50.1, 48.3, 47.5, 45.5, 28.5, 27.0, 23.1. **LCMS** ($C_{18}H_{25}BrN_2O_3$) $[M+H]^+$ required 397.1, $[M+H]^+$ not found, poor ionisation. (Formic) R_t = 2.97 min.

4'-(4-(_tert_-butoxycarbonyl)-1,4-diazepane-1-carbonyl)-2'-methyl-[1,1'-biphenyl]-3-carboxylic acid (10h)

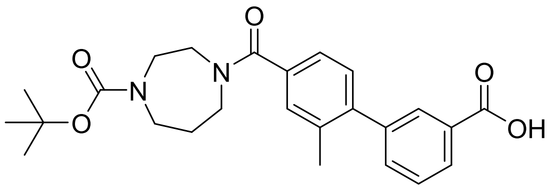

10h

To a mixture of *tert*-butyl 4-(4-bromo-3-methylbenzoyl)-1,4-diazepane-1-carboxylate **10f** (193 mg, 0.486 mmol), 3-boronobenzoic acid **10g** (81 mg, 0.488 mmol), K_2CO_3 (181 mg, 1.31 mmol) and $Pd(0)(PPh_3)_4$ (4 mol%, 22.4 mg, 0.0194 mmol), under a nitrogen atmosphere, was added 1,4-dioxane/H_2O (10:1, 2.75 mL). The reaction mixture was heated to 120 °C with stirring in a microwave reactor for 30 min. The reaction mixture was filtered through celite and the pad was washed with DCM (5 mL). The filtrate was concentrated under reduced pressure, and the residue was taken up in water (20 mL). The aqueous was acidified to pH 4 with an aqueous solution of 1 M HCl, and extracted with EtOAc (2 x 20 mL). The combined organics were dried through a phase separator and concentrated under reduced pressure. The residue was purified by flash column chromatography (normal phase, eluted with 10 – 90% EtOAc in cyclohexane) to yield 4'-(4-(*tert*-butoxycarbonyl)-1,4-diazepane-1-carbonyl)-2'-methyl-[1,1'-biphenyl]-3-carboxylic acid **10h**, as a gum (112 mg, 0.255 mmol, 52%). **^{1}H NMR** (400 MHz, CDCl3) δ 8.12 – 8.08 (m, 1H), 8.05 (s, 1H), 7.56 – 7.52 (m, 2H), 7.30 (s, 1H), 7.25 (s, 1H), 3.86 – 3.80 (m, 1H), 3.73 (t, *J* = 6.1 Hz, 1H), 3.67 – 3.62 (m, 1H), 3.59 – 3.51 (m, 2H), 3.47 (t, *J* = 6.3 Hz, 3H), 2.27 (s, 3H), 2.03 – 1.97 (m, 1H), 1.81 – 1.69 (m, 1H), 1.49 (s, 9H). **^{13}C NMR** (101 MHz, $CDCl_3$) δ 194.3, 171.9, 154.9, 142.0, 141.6, 136.1, 134.3, 132.3, 130.8, 130.0, 129.7, 129.1, 128.6, 124.2, 72.7, 68.7, 60.6, 49.3, 47.5, 28.6, 20.5. **LCMS** ($C_{25}H_{30}N_2O_5$) $[M-H]^-$ required 437.2, $[M-H]^-$ found 437.2. (Formic) R_t = 2.66 min.

tert-butyl (*l*)-4-(3'-((2-(2-(2-(2-(4-(4-chlorophenyl)-2,3,9-trimethyl-6H-thieno[3,2-f][1,2,4]triazolo[4,3-a][1,4]diazepin-6-yl)acetamido)ethoxy)ethoxy)ethyl)carbamoyl)-2-methyl-[1,1'-biphenyl]-4-carbonyl)-1,4-diazepane-1-carboxylate (HB1b)

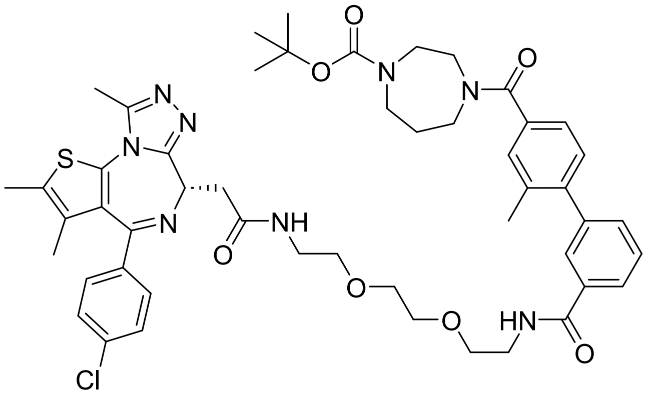

HB1b

To a solution of 4'-(4-(*tert*-butoxycarbonyl)-1,4-diazepane-1-carbonyl)-2'-methyl-[1,1'-biphenyl]-3-carboxylic acid **10h** (23.0 mg, 0.0525 mmol) in DCM/DMF (3:1, 1 mL) was added DIPEA (50 µL, 0.287 mmol), and the solution was stirred for 5 min. HATU (31.5 mg, 0.0828 mmol) was added and the reaction mixture was stirred for 10 min. (*S*)-*N*-(2-(2-(2-aminoethoxy)ethoxy)ethyl)-2-(4-(4-chlorophenyl)-2,3,9-trimethyl-6H-thieno[3,2-f][1,2,4]triazolo[4,3-a][1,4]diazepin-6-yl)acetamide hydrogen chloride **HB1a** (30.8 mg, 0.0543 mmol) was added to the reaction mixture, and stirred at room temperature for 18 h. The reaction mixture was separated with DCM (3 x 10 mL) and a saturated aqueous solution of NH_4Cl (10 mL). The combined organics were dried through a phase separator, and concentrated under reduced pressure. The residue was purified by flash chromatography (reverse-phase, eluted with 0 – 80% MeCN (with 0.1% formic acid) in water (with 0.1% formic acid)) to yield *tert*-butyl (*S*)-4-(3'-((2-(2-(2-(2-(4-(4-chlorophenyl)-2,3,9-trimethyl-6H-thieno[3,2-f][1,2,4]triazolo[4,3-a][1,4]diazepin-6-yl)acetamido)ethoxy)ethoxy)ethyl)carbamoyl)-2-methyl-[1,1'-biphenyl]-4-carbonyl)-1,4-diazepane-1-carboxylate **HB1b**, as a clear gum (17.6 mg, 0.0185 mmol, 35%). **^{1}H NMR** (400 MHz, DMSO-d6) δ 8.60 (t, *J* = 5.6 Hz, 1H), 8.26 (t, *J* = 5.7 Hz, 1H), 7.89 – 7.85 (m, 1H), 7.84 (s, 1H), 7.53 (d, *J* = 6.1 Hz, 1H), 7.47 (d, *J* = 8.7 Hz, 2H), 7.41 (d, *J* = 8.7 Hz, 2H), 7.32 – 7.20 (m, 4H), 4.50 (dd, *J* = 7.9, 6.2 Hz, 1H), 3.70 (br s, 1H), 3.61 (br s, 1H), 3.57 – 3.51 (m, 6H) 3.49 – 3.36 (m, 9H), 3.28 – 3.16 (m, 5H), 2.58 (s, 3H), 2.40 (s, 3H), 2.24 (s, 3H), 1.77 (br s, 1H), 1.61 (s, 3H), 1.58 (br s, 1H), 1.42 (d, *J* = 2.8 Hz, 7H), 1.28 (s, 2H). **^{13}C NMR** (101 MHz, DMSO-d6) δ 194.0, 170.2, 169.7, 166.0, 163.2, 163.0, 155.1, 149.8, 140.5, 136.8, 135.2, 134.4, 132.3, 131.6, 130.7, 130.2, 129.8, 129.6, 128.5, 78.7, 69.6, 69.2, 68.9, 53.8, 38.6,

37.5, 28.1, 20.0, 14.1, 12.7, 11.3. **LCMS** ($C_{50}H_{59}ClN_8O_7S$) $[M+H]^+$ required 951.4, $[M+H]^+$ found 951.5. (Formic) R_t = 2.99 min.

(*S*)-4'-(4-(2-chloroacetyl)-1,4-diazepane-1-carbonyl)-*N*-(2-(2-(2-(2-(4-(4-chlorophenyl)-2,3,9-trimethyl-6H-thieno[3,2-f][1,2,4]triazolo[4,3-a][1,4]diazepin-6-yl)acetamido)ethoxy)ethoxy)ethyl)-2'-methyl-[1,1'-biphenyl]-3-carboxamide (HB1)

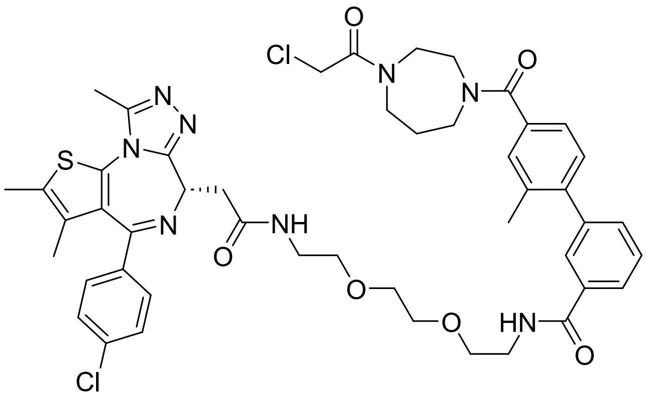

HB1

To a solution of Boc-protected amine **HB1b** (11.8 mg, 0.0139 mmol) in 1,4-dioxane (1 mL) was added HCl in 1,4-dioxane (4 M, 200 µL, 0.796 mmol), and the reaction mixture was stirred for 4 h. The reaction mixture was concentrated under reduced pressure, co-evaporating with DCM (3 x 10 mL). The residue was resuspended in DMF (0.5 mL), under a nitrogen atmosphere at 0 °C, NEt_3 (50 µL, 0.367 mmol) was added, and the reaction mixture was stirred for 5 min. Chloroacetyl chloride (5 µL, 0.0628 mmol) was added dropwise at 0 °C, and the reaction mixture was stirred for 10 min. The reaction mixture was purified by flash column chromatography (reverse-phase, eluted with 0 – 60% MeCN (with 0.1% formic acid) in water (with 0.1% formic acid)). Freeze-drying afforded (*S*)-4'-(4-(2-chloroacetyl)-1,4-diazepane-1-carbonyl)-*N*-(2-(2-(2-(2-(4-(4-chlorophenyl)-2,3,9-trimethyl-6H-thieno[3,2-f][1,2,4]triazolo[4,3-a][1,4]diazepin-6-yl)acetamido)ethoxy)ethoxy)ethyl)-2'-methyl-[1,1'-biphenyl]-3-carboxamide (**HB1**) as a clear gum (7.7 mg, 0.0083 mmol, 60%). **^{1}H NMR** (400 MHz, DMSO-d6) δ 8.59 (t, *J* = 5.5 Hz, 1H), 8.26 (t, *J* = 5.7 Hz, 1H), 7.89 – 7.85 (m, 1H), 7.83 (s, 1H), 7.53 (d, *J* = 6.1 Hz, 1H), 7.48 (d, *J* = 8.7 Hz, 2H), 7.41 (d, *J* = 8.6 Hz, 2H), 7.34 – 7.20 (m, 4H), 4.49 (dd, *J* = 7.9, 6.2 Hz, 1H), 4.40 (s, 2H), 3.83 – 3.77 (m, 1H), 3.67

(br s, 2H), 3.62 – 3.51 (m, 8H), 3.49 – 3.38 (m, 5H), 3.28 – 3.15 (m, 6H), 2.58 (s, 3H), 2.40 (s, 3H), 2.24 (s, 3H), 1.87 (br s, 1H), 1.61 (s, 3H), 1.57 (br s, 1H). **^{13}C NMR** (101 MHz, DMSO-d6) δ 169.7, 166.0, 163.0, 155.1, 149.8, 141.4, 140.5, 136.8, 135.2, 134.5, 132.3, 131.6, 130.7, 130.2, 129.9, 129.6, 128.5, 128.4, 127.5, 126.3, 117.1, 110.5, 84.6, 77.7, 69.6, 69.2, 68.9, 53.9, 46.6, 38.6, 37.5, 20.1, 14.1, 12.7, 11.3. **LCMS** ($C_{47}H_{52}Cl_2N_8O_6S$) $[M+H]^+$ required 927.3, $[M+H]^+$ found 927.5. (Formic) R_t = 2.66 min. **HRMS** ($C_{47}H_{52}Cl_2N_8O_6S$) $[M+H]^+$ required 927.3186, $[M+H]^+$ found 927.3174.

tert-butyl (_S_)-4-(3'-((1-(4-(4-chlorophenyl)-2,3,9-trimethyl-6H-thieno[3,2-f][1,2,4]triazolo[4,3-a][1,4]diazepin-6-yl)-2-oxo-6,9,12-trioxa-3-azatetradecan-14-yl)carbamoyl)-2-methyl-[1,1'-biphenyl]-4-carbonyl)-1,4-diazepane-1-carboxylate (HB2b)

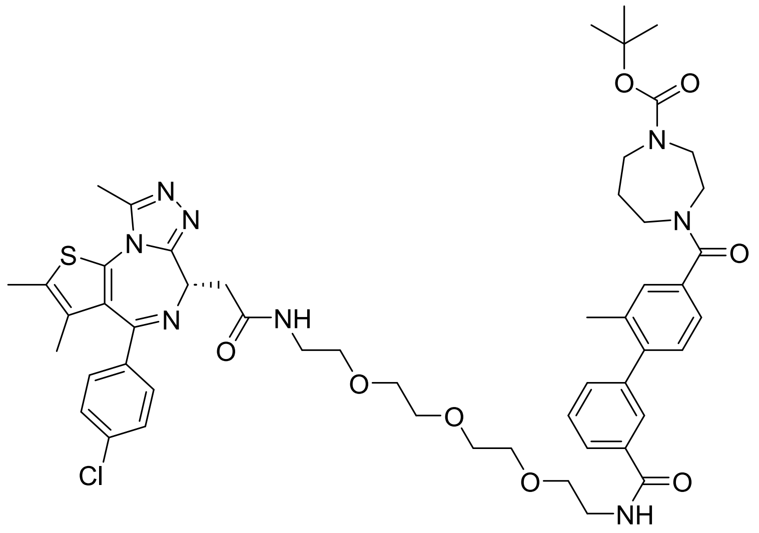

HB2b

To a solution of 4'-(4-(*tert*-butoxycarbonyl)-1,4-diazepane-1-carbonyl)-2'-methyl-[1,1'-biphenyl]-3-carboxylic acid **10h** (23.0 mg, 0.0525 mmol) in DCM/DMF (2:1, 1 mL) was added DIPEA (50 µL, 0.287 mmol), and the solution was stirred for 5 min. HATU (24.9 mg, 0.0655 mmol) was added and the reaction mixture was stirred for 10 min. (*S*)-*N*-(2-(2-(2-(2-aminoethoxy)ethoxy)ethoxy)ethyl)-2-(4-(4-chlorophenyl)-2,3,9-trimethyl-6H-thieno[3,2-f][1,2,4]triazolo[4,3-a][1,4]diazepin-6-yl)acetamide **HB2a** (33.2 mg, 0.0577 mmol) was added to the reaction mixture, and stirred at room temperature for 3 h. The reaction mixture was separated with DCM (3 x 10 mL) and a saturated aqueous solution of NH_4Cl (10 mL). The combined organics were dried through a

phase separator, and concentrated under reduced pressure. The residue was purified by flash column chromatography (reverse-phase, eluted with 0 – 80% MeCN (with 0.1% formic acid) in water (with 0.1% formic acid)) to yield *tert*-butyl (*S*)-4-(3'-((1-(4-(4-chlorophenyl)-2,3,9-trimethyl-6H-thieno[3,2-f][1,2,4]triazolo[4,3-a][1,4]diazepin-6-yl)-2-oxo-6,9,12-trioxa-3-azatetradecan-14-yl)carbamoyl)-2-methyl-[1,1'-biphenyl]-4-carbonyl)-1,4-diazepane-1-carboxylate **HB2b**, as a clear gum (37.8 mg, 0.0380 mmol, 72%). **^{1}H NMR** (400 MHz, DMSO-d6) δ 8.59 (t, *J* = 5.7 Hz, 1H), 8.26 (t, *J* = 5.6 Hz, 1H), 7.89 – 7.85 (m, 1H), 7.83 (s, 1H), 7.53 (d, *J* = 6.1 Hz, 2H), 7.47 (d, *J* = 8.8 Hz, 2H), 7.41 (d, *J* = 8.6 Hz, 2H), 7.33 – 7.19 (m, 3H), 4.50 (dd, *J* = 8.0, 6.1 Hz, 1H), 3.70 (br s, 1H), 3.63 – 3.59 (m, 1H), 3.54 – 3.48 (m, 11H), 3.45 – 3.63 (m, 8H), 3.29 – 3.16 (m, 5H), 2.58 (s, 3H), 2.40 (s, 3H), 2.24 (s, 3H), 1.77 (br s, 1H), 1.61 (s, 3H), 1.57 (br s, 1H), 1.41 (d, *J* = 2.8 Hz, 7H), 1.28 (s, 2H). **^{13}C NMR** (101 MHz, DMSO-d6) δ 169.7, 163.0, 155.1, 149.9, 136.8, 135.2, 132.4, 131.6, 130.7, 130.2, 129.8, 129.6, 128.5, 127.6, 78.8, 69.8, 69.6, 69.3, 68.9, 53.8, 38.6, 37.5, 28.1, 20.0, 14.1, 12.7, 11.3. **LCMS** ($C_{52}H_{63}ClN_8O_8S$) $[M+H]^+$ required 995.4, $[M+H]^+$ found 995.5. (Formic) R_t = 3.00 min.

(*S*)-4'-(4-(2-chloroacetyl)-1,4-diazepane-1-carbonyl)-*N*-(1-(4-(4-chlorophenyl)-2,3,9-trimethyl-6H-thieno[3,2-f][1,2,4]triazolo[4,3-a][1,4]diazepin-6-yl)-2-oxo-6,9,12-trioxa-3-azatetradecan-14-yl)-2'-methyl-[1,1'-biphenyl]-3-carboxamide (HB2)

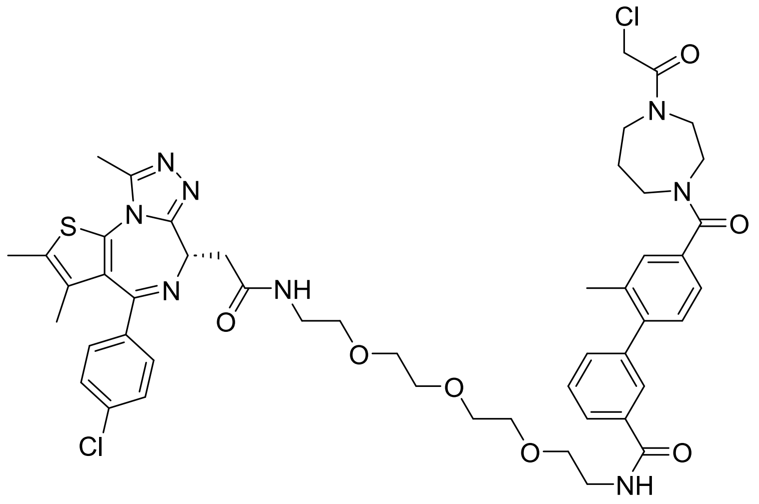

HB2

To a solution of Boc-protected amine **HB2b** (33.8 mg, 0.0339 mmol) in 1,4-dioxane (1 mL) was added HCl in 1,4-dioxane (4 M, 350 µL, 1.39 mmol), and the reaction mixture was stirred for 4 h. The reaction mixture was concentrated under reduced pressure, co-evaporating with DCM (3 x 10 mL). The residue was resuspended in DMF (0.5 mL), under a nitrogen atmosphere at 0 °C, NEt_3 (20 µL, 0.147 mmol) was added, and the reaction mixture was stirred for 5 min. Chloroacetyl chloride (10 µL, 0.126 mmol) was added dropwise at 0 °C, and the reaction mixture was stirred for 10 min. The reaction mixture was purified by flash column chromatography (reverse-phase, eluted with 0 – 50% MeCN (with 0.1% formic acid) in water (with 0.1% formic acid)). Freeze-drying afforded (*S*)-4'-(4-(2-chloroacetyl)-1,4-diazepane-1-carbonyl)-*N*-(1-(4-(4-chlorophenyl)-2,3,9-trimethyl-6H-thieno[3,2-f][1,2,4]triazolo[4,3-a][1,4]diazepin-6-yl)-2-oxo-6,9,12-trioxa-3-azatetradecan-14-yl)-2'-methyl-[1,1'-biphenyl]-3-carboxamide (**HB2**) as a fluffy off-white solid (19.2 mg, 0.0198 mmol, 58%). **^{1}H NMR** (400 MHz, DMSO-d6) δ 8.59 (t, *J* = 5.6 Hz, 1H), 8.26 (t, *J* = 5.6 Hz, 1H), 7.89 – 7.85 (m, 1H), 7.83 (s, 1H), 7.55 – 7.52 (m, 2H), 7.49 – 7.45 (m, 2H), 7.43 – 7.39 (m, 2H), 7.35 – 7.20 (m, 3H), 4.50 (dd, *J* = 8.0, 6.1 Hz, 1H), 4.40 (s, 2H), 3.67 (br s, 2H), 3.62 – 3.55 (m, 4H), 3.52 – 3.49 (m, 8H), 3.45 – 3.39 (m, 5H), 3.30 – 3.16 (m, 7H), 2.58 (s, 3H), 2.40 (s, 3H), 2.24 (s, 3H), 1.88 (br s, 1H), 1.61 (s, 3H), 1.56 (br s, 1H). **^{13}C NMR** (101 MHz, DMSO-d6) δ 169.7, 166.0, 165.7, 163.0, 155.1, 149.8, 141.4, 140.5, 136.8, 135.2, 134.4, 132.3, 131.6, 130.7, 130.2, 129.8, 129.6, 128.5, 128.4, 127.5, 126.3, 69.8, 69.6, 69.2, 68.9, 53.8, 46.6, 41.6, 38.6, 37.5, 20.1, 14.1, 12.7, 11.3. **LCMS** ($C_{49}H_{56}Cl_2N_8O_7S$) $[M+H]^+$ required 971.3, $[M+H]^+$ found 971.5. (Formic) R_t = 2.67 min. **HRMS** ($C_{49}H_{56}Cl_2N_8O_7S$) $[M+H]^+$ required 971.3448, $[M+H]^+$ found 971.3470.

tert-butyl (_S_)-4-(3'-((1-(4-(4-chlorophenyl)-2,3,9-trimethyl-6H-thieno[3,2-f][1,2,4]triazolo[4,3-a][1,4]diazepin-6-yl)-2-oxo-6,9,12,15-tetraoxa-3-azaheptadecan-17-yl)carbamoyl)-2-methyl-[1,1'-biphenyl]-4-carbonyl)-1,4-diazepane-1-carboxylate (HB3b)

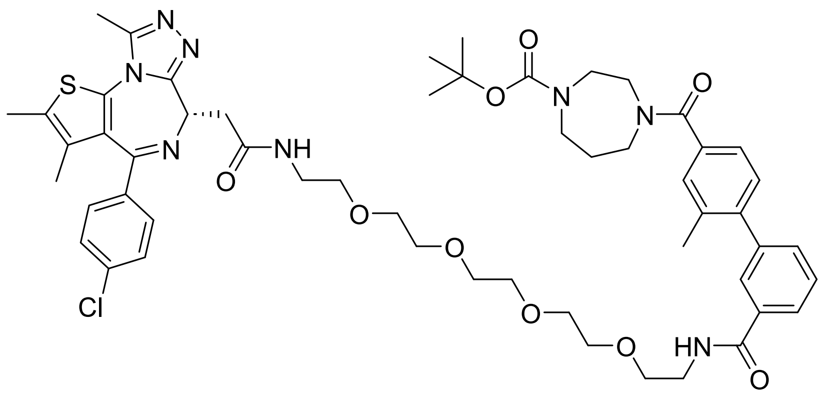

HB3b

To a solution of 4'-(4-(*tert*-butoxycarbonyl)-1,4-diazepane-1-carbonyl)-2'-methyl-[1,1'-biphenyl]-3-carboxylic acid **10h** (24.7 mg, 0.0563 mmol) in DCM/DMF (2:1, 1 mL) was added DIPEA (50 µL, 0.287 mmol), and the solution was stirred for 5 min. HATU (30.5 mg, 0.0802 mmol) was added and the reaction mixture was stirred for 10 min. (*S*)-*N*-(14-amino-3,6,9,12-tetraoxatetradecyl)-2-(4-(4-chlorophenyl)-2,3,9-trimethyl-6H-thieno[3,2-f][1,2,4]triazolo[4,3-a][1,4]diazepin-6-yl)acetamide **HB3a** (41.7 mg, 0.0673 mmol) was added to the reaction mixture, and stirred at room temperature for 3 h. The reaction mixture was separated with DCM (3 x 10 mL) and a saturated aqueous solution of NH_4Cl (10 mL). The combined organics were dried through a phase separator, and concentrated under reduced pressure. The residue was purified by flash column chromatography (reverse-phase, eluted with 0 – 80% MeCN (with 0.1% formic acid) in water (with 0.1% formic acid)). Freeze-drying afforded *tert*-butyl (*S*)-4-(3'-((1-(4-(4-chlorophenyl)-2,3,9-trimethyl-6H-thieno[3,2-f][1,2,4]triazolo[4,3-a][1,4]diazepin-6-yl)-2-oxo-6,9,12,15-tetraoxa-3-azaheptadecan-17-yl)carbamoyl)-2-methyl-[1,1'-biphenyl]-4-carbonyl)-1,4-diazepane-1-carboxylate **HB3b**, as a fluffy off-white solid (28.2 mg, 0.0271 mmol, 48%). **^{1}H NMR** (400 MHz, DMSO-d6) δ 8.58 (t, *J* = 5.5 Hz, 1H), 8.27 (t, *J* = 5.6 Hz, 1H), 7.89 – 7.85 (m, 1H), 7.83 (s, 1H), 7.53 (d, *J* = 5.8 Hz, 2H), 7.48 (d, *J* = 8.8 Hz, 2H), 7.41 (d, *J* = 8.6 Hz, 2H), 7.33 – 7.19 (m, 3H), 4.50 (dd, *J* = 8.1, 6.0 Hz, 1H), 3.70 (br s, 1H), 3.63 – 3.58 (m, 2H), 3.56 – 3.47 (m, 13H), 3.46 – 3.36 (m, 8H), 3.30 – 3.16 (m, 6H), 2.59 (s, 3H), 2.40 (s, 3H), 2.24 (s, 3H), 1.77 (br s, 1H), 1.61 (s, 3H), 1.58 (br s, 1H), 1.41 (d, *J* = 2.6 Hz, 7H), 1.28 (s, 2H). **^{13}C NMR** (101 MHz, DMSO-d6) δ 169.7, 166.0, 163.0, 155.1, 149.8, 143.4, 140.5, 136.8, 135.2, 132.3, 131.6, 130.2, 129.9, 129.6, 128.5, 69.8, 69.6, 69.2, 68.9, 65.5, 53.9,

38.6, 37.5, 28.1, 27.9, 20.0, 14.1, 12.7, 11.3. **LCMS** ($C_{54}H_{67}ClN_8O_9S$) $[M+H]^+$ required 1039.4, $[M+H]^+$ found 1039.6. (Formic) R_t = 3.00 min.

(*S*)-4'-(4-(2-chloroacetyl)-1,4-diazepane-1-carbonyl)-*N*-(1-(4-(4-chlorophenyl)-2,3,9-trimethyl-6H-thieno[3,2-f][1,2,4]triazolo[4,3-a][1,4]diazepin-6-yl)-2-oxo-6,9,12,15-tetraoxa-3-azaheptadecan-17-yl)-2'-methyl-[1,1'-biphenyl]-3-carboxamide (HB3)

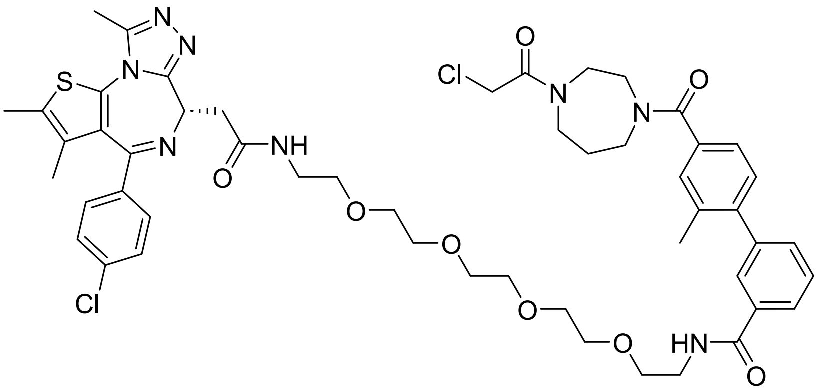

HB3

To a solution of Boc-protected amine **HB3b** (25.0 mg, 0.0240 mmol) in 1,4-dioxane (1 mL) was added HCl in 1,4-dioxane (4 M, 200 µL, 0.796 mmol), and the reaction mixture was stirred for 2 h. The reaction mixture was concentrated under reduced pressure, co-evaporating with DCM (3 x 10 mL). The residue was resuspended in DMF (0.5 mL), under a nitrogen atmosphere at 0 °C, NEt_3 (20 µL, 0.147 mmol) was added, and the reaction mixture was stirred for 5 min. Chloroacetyl chloride (10 µL, 0.126 mmol) was added dropwise at 0 °C, and the reaction mixture was stirred for 10 min. The reaction mixture was purified by flash column chromatography (reverse-phase, eluted with 0 – 60% MeCN (with 0.1% formic acid) in water (with 0.1% formic acid)). Freeze-drying afforded (*S*)-4'-(4-(2-chloroacetyl)-1,4-diazepane-1-carbonyl)-*N*-(1-(4-(4-chlorophenyl)-2,3,9-trimethyl-6H-thieno[3,2-f][1,2,4]triazolo[4,3-a][1,4]diazepin-6-yl)-2-oxo-6,9,12,15-tetraoxa-3-azaheptadecan-17-yl)-2'-methyl-[1,1'-biphenyl]-3-carboxamide (**HB3**) as a fluffy off-white solid (10.4 mg, 0.0102 mmol, 43%). **^{1}H NMR** (400 MHz, DMSO-d6) δ 8.58 (t, *J* = 5.5 Hz, 1H), 8.27 (t, *J* = 5.6 Hz, 1H), 7.89 – 7.85 (m, 1H), 7.83 (s, 1H), 7.55 – 7.52 (m, 2H), 7.50 – 7.45 (m, 2H), 7.44 – 7.39 (m, 2H), 7.35 – 7.21 (m, 3H), 4.50 (dd, *J* = 8.1, 6.0 Hz, 1H), 4.41 (s, 2H), 3.80 (br s, 1H), 3.67

(br s, 3H), 3.62 – 3.47 (m, 16H), 3.46 – 3.39 (m, 6H), 3.30 – 3.16 (m, 4H), 2.59 (s, 3H), 2.40 (s, 3H), 2.24 (s, 3H), 1.88 (br s, 1H), 1.61 (s, 3H), 1.56 (br s, 1H). **^{13}C NMR** (101 MHz, DMSO-d6) δ 169.7, 166.0, 165.7, 163.1, 155.1, 149.9, 141.4, 140.5, 136.7, 135.3, 134.5, 132.2, 131.6, 130.8, 130.2, 129.9, 129.6, 128.5, 128.4, 127.5, 126.3, 123.8, 69.8, 69.8, 69.6, 69.2, 68.9, 53.8, 42.3, 38.7, 37.5, 20.1, 14.1, 12.7, 11.3. **LCMS** ($C_{51}H_{60}Cl_2N_8O_8S$) $[M+H]^+$ required 1015.4, $[M+H]^+$ found 1015.4. (Formic) R_t = 2.68 min. **HRMS** ($C_{51}H_{60}Cl_2N_8O_8S$) $[M+H]^+$ required 1015.3710, $[M+H]^+$ found 1015.3747.

References

1 A. M. Waterhouse, J. B. Procter, D. M. A. Martin, M. Clamp and G. J. Barton, Jalview Version 2 - a multiple sequence alignment editor and analysis workbench, *Bioinformatics*, 2009, **25**, 1189–1191.

2 K. Hoegenauer, S. An, J. Axford, C. Benander, C. Bergsdorf, J. Botsch, S. Chau, C. Fernández, S. Gleim, U. Hassiepen, J. Hunziker, E. Joly, A. Keller, S. Lopez Romero, R. Maher, A.-S. Mangold, C. Mickanin, M. Mihalic, P. Neuner, A. W. Patterson, F. Perruccio, S. Roggo, J. Scesa, M. Schröder, D. Shkoza, B. Thai, A. Vulpetti, M. Renatus and J. S. Reece-Hoyes, Discovery of Ligands for TRIM58, a Novel Tissue-Selective E3 Ligase, *ACS Med Chem Lett*, 2023, **14**, 1631–1639.

3 P. Lu, Y. Cheng, L. Xue, X. Ren, X. Xu, C. Chen, L. Cao, J. Li, Q. Wu, S. Sun, J. Hou, W. Jia, W. Wang, Y. Ma, Z. Jiang, C. Li, X. Qi, N. Huang and T. Han, Selective degradation of multimeric proteins by TRIM21-based molecular glue and PROTAC degraders., *Cell*, 2024, **187**, 1–17.

4 C. J. Muñoz Sosa, C. Lenz, A. Hamann, F. Farges, J. Dopfer, A. Krämer, V. Cherkashyna, A. Tarnovskiy, Y. S. Moroz, E. Proschak, V. Němec, S. Müller, K. Saxena and S. Knapp, A C-Degron Structure-Based Approach for the Development of Ligands Targeting the E3 Ligase TRIM7, *ACS Chem Biol*, 2024, **19**, 1638–1647.

5 M. G. Koliopoulos, D. Esposito, E. Christodoulou, I. A. Taylor and K. Rittinger, Functional role of TRIM E3 ligase oligomerization and regulation of catalytic activity , *EMBO J*, 2016, **35**, 1204–1218.

6 A. F. Carvalho, M. P. Pinto, C. P. Grou, R. Vitorino, P. Domingues, F. Yamao, C. Sá-Miranda and J. E. Azevedo, High-yield expression in Escherichia coli and purification of mouse ubiquitin-activating enzyme E1, *Mol Biotechnol*, 2012, **51**, 254–261.

7 B. Stieglitz, A. C. Morris-Davies, M. G. Koliopoulos, E. Christodoulou and K. Rittinger, LUBAC synthesizes linear ubiquitin chains via a thioester intermediate, *EMBO Rep*, 2012, **13**, 840–846.

8 A. Chaikuad, R. Zhubi, C. Tredup and S. Knapp, Comparative structural analyses of the NHL domains from the human E3 ligase TRIM–NHL family, *IUCrJ*, 2022, **9**, 720–727.

9 A. Vuorinen, C. R. Kennedy, K. A. McPhie, W. McCarthy, J. Pettinger, J. M. Skehel, D. House, J. T. Bush and K. Rittinger, Enantioselective OTUD7B fragment discovery through chemoproteomics screening and high-throughput optimisation, *Commun Chem*, 2025, **8**, 12.

10 M. G. Koliopoulos, M. Lethier, A. G. Van Der Veen, K. Haubrich, J. Hennig, E. Kowalinski, R. V. Stevens, S. R. Martin, C. Reis E Sousa, S. Cusack and K. Rittinger, Molecular mechanism of influenza A NS1-mediated TRIM25 recognition and inhibition, *Nat Commun*, 2018, **9**, 1820.

11 H. Wilders, G. Biggs, S. M. Rowe, E. E. Cawood, A. R. Rendina, E. K. Grant, I. G. Riziotis, J. Pettinger, D. J. Fallon, M. Skehel, D. House, N. C. O. Tomkinson and J. Bush, Expedited SARS-CoV-2 Main Protease Inhibitor Discovery Through Modular 'Direct-To-Biology' Screening, *Angewandte Chemie International Edition*, 2024, e202418314.

12 N. Tanaka, K. Ohno, T. Niimi, A. Moritomo, K. Mori and M. Orita, Small-world phenomena in chemical library networks: Application to fragment-based drug discovery, *J Chem Inf Model*, 2009, **49**, 2677–2686.

13 J. Mason, H. Wilders, D. J. Fallon, R. P. Thomas, J. T. Bush, N. C. O. Tomkinson and F. Rianjongdee, Automated LC-MS analysis and data extraction for high-throughput chemistry, *Digital Discovery*, 2023, **2**, 1894–1899.

14 G. Picco, Y. Rao, A. Al Saedi, Y. Lee, S. F. Vieira, S. Bhosle, K. May, C. Herranz-Ors, S. J. Walker, R. Shenje, C. Dincer, F. Gibson, R. Banerjee, Z. Hewitson, T. Werner, J. E. Cottom, Y. Peng, N. Deng, Y. Zhang, E. N. Nartey, L. Nickels, P. Landis, D. Conticelli, K. McCarten, J. Bush, M. Sharma, H. Lightfoot, D. House, E. Milford, E. K. Grant, M. P. Glogowski, C. D. Wagner, M. Bantscheff, A. Rutkowska-Klute, F. Zappacosta, J. Pettinger, S. Barthorpe, H. C. Eberl, B. T. Jones, J. L. Schneck, D. J. Murphy, E. E. Voest, J. P. Taygerly, M. P. DeMartino, M. A. Coelho, J. Houseley, G. Sharma, B. Schwartz and M. J. Garnett, Novel WRN Helicase Inhibitors Selectively Target Microsatellite-Unstable Cancer Cells, *Cancer Discov*, 2024, **14**, 1457–1475.

15 N. Zinn, T. Werner, C. Doce, T. Mathieson, C. Boecker, G. Sweetman, C. Fufezan and M. Bantscheff, Improved Proteomics-Based Drug Mechanism-of-Action Studies Using 16-Plex Isobaric Mass Tags, *J Proteome Res*, 2021, **20**, 1792–1801.

16 M. M. Savitski, T. Mathieson, N. Zinn, G. Sweetman, C. Doce, I. Becher, F. Pachl, B. Kuster and M. Bantscheff, Measuring and managing ratio compression for accurate iTRAQ/TMT quantification, *J Proteome Res*, 2013, **12**, 3586–3598.

17 G. Winter, J. Beilsten-Edmands, N. Devenish, M. Gerstel, R. J. Gildea, D. McDonagh, E. Pascal, D. G. Waterman, B. H. Williams and G. Evans, DIALS as a toolkit, *Protein Science*, 2022, **31**, 232–250.

18 P. R. Evans and G. N. Murshudov, How good are my data and what is the resolution?, *Acta Crystallogr D Biol Crystallogr*, 2013, **69**, 1204–1214.

19 P. D. Adams, P. V. Afonine, G. Bunkóczi, V. B. Chen, I. W. Davis, N. Echols, J. J. Headd, L. W. Hung, G. J. Kapral, R. W. Grosse-Kunstleve, A. J. McCoy, N. W. Moriarty, R. Oeffner, R. J. Read, D. C. Richardson, J. S. Richardson, T. C. Terwilliger and P. H. Zwart, PHENIX: A comprehensive Python-based system for macromolecular structure solution, *Acta Crystallogr D Biol Crystallogr*, 2010, **66**, 213–221.

20 P. Emsley and K. Cowtan, Coot: Model-building tools for molecular graphics, *Acta Crystallogr D Biol Crystallogr*, 2004, **60**, 2126–2132.

21 G. N. Murshudov, A. A. Vagin and E. J. Dodson, Refinement of macromolecular structures by the maximum-likelihood method, *Acta Crystallogr D Biol Crystallogr*, 1997, **53**, 240–255.

22 D. Franke, M. V. Petoukhov, P. V. Konarev, A. Panjkovich, A. Tuukkanen, H. D. T. Mertens, A. G. Kikhney, N. R. Hajizadeh, J. M. Franklin, C. M. Jeffries and D. I. Svergun, ATSAS 2.8: A comprehensive data analysis suite for small-angle scattering from macromolecular solutions, *J Appl Crystallogr*, 2017, **50**, 1212–1225.

23 R. P. Rambo and J. A. Tainer, Characterizing flexible and intrinsically unstructured biological macromolecules by SAS using the Porod-Debye law, *Biopolymers*, 2011, **95**, 559–571.

24 D. Lu, C. A. Foley, S. V. Birla, A. J. Hepperla, J. M. Simon, L. I. James and N. A. Hathaway, Bioorthogonal Chemical Epigenetic Modifiers Enable Dose-Dependent CRISPR Targeted Gene Activation in Mammalian Cells, *ACS Synth Biol*, 2022, **11**, 1397–1407.

25 J. M. Chalker, C. S. C. Wood and B. G. Davis, A convenient catalyst for aqueous and protein Suzuki-Miyaura cross-coupling, *J Am Chem Soc*, 2009, **131**, 16346–16347.
